# Design and Pharmacological Characterization of α_4_β_1_ Integrin Cyclopeptide Agonists: Computational Investigation of Ligand Determinants for Agonism versus Antagonism

**DOI:** 10.1021/acs.jmedchem.2c02098

**Published:** 2023-03-28

**Authors:** Michele Anselmi, Monica Baiula, Santi Spampinato, Roberto Artali, Tingting He, Luca Gentilucci

**Affiliations:** † Department of Chemistry “G. Ciamician”, University of Bologna, Via Selmi 2, 40126 Bologna, Italy; ¥ Health Sciences & Technologies (HST) CIRI, University of Bologna, Via Tolara di Sopra 41/E, 40064 Ozzano Emilia, Italy; ‡ Department of Pharmacy and Biotechnology, University of Bologna, Via Irnerio 48, 40126, Bologna, Italy; § Scientia Advice, 20832 Desio, Italy

## Abstract

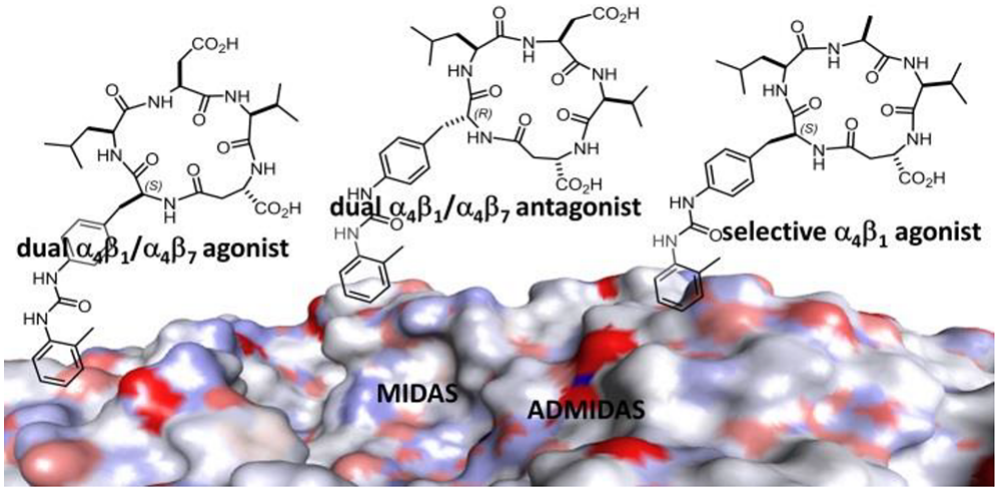

α_4_β_1_ integrin is a cell adhesion receptor deeply involved in the migration and accumulation of leukocytes. Therefore, integrin antagonists that inhibit leukocytes recruitment are currently regarded as a therapeutic opportunity for the treatment of inflammatory disorder, including leukocyte-related autoimmune diseases. Recently, it has been suggested that integrin agonists capable to prevent the release of adherent leukocytes might serve as therapeutic agents as well. However, very few α_4_β_1_ integrin agonists have been discovered so far, thus precluding the investigation of their potential therapeutic efficacy. In this perspective, we synthesized cyclopeptides containing the LDV recognition motif found in the native ligand fibronectin. This approach led to the discovery of potent agonists capable to increase the adhesion of α_4_ integrin-expressing cells. Conformational and quantum mechanics computations predicted distinct ligand–receptor interactions for antagonists or agonists, plausibly referable to receptor inhibition or activation.

## Introduction

α_4_β_1_ integrin, also known as very late antigen-4 (VLA-4), is a heterodimeric cell surface receptor expressed on most leukocytes, fundamental to their homing, trafficking, differentiation, activation, and survival. The natural ligands of this receptor are the protein of the extracellular matrix (ECM), fibronectin (FN), and the vascular cell adhesion molecule-1 (VCAM-1) expressed on endothelial cells.^1,2^ The binding sequence in FN is the tripeptide Leu-Asp-Val (LDV) found in the alternatively spliced connecting segment 1 (CS-1) region, while VCAM-1 is recognized through the fragment Ile-Asp-Ser (IDS).^3^ The α_4_ subunit can couple also with the β_7_ subunit; the natural ligand of the resulting α_4_β_7_ dimer is the mucosal vascular addressin cell adhesion molecule-1 (MAdCAM-1), whose peptidic recognition motif is Leu-Asp-Thr (LDT).^3^

α_4_β_1_ integrin is involved in the development and sustainment of inflammation, in several inflammation-related diseases, and in cancer development, metastasis, and stem cell mobilization or retention.^4,5^ This receptor is also involved in T cell migration across the blood–brain barrier (BBB) in autoimmune encephalitis (AE).^4,5^ In multiple sclerosis (MS), autoreactive T lymphocytes are recruited into the CNS through the interaction between α_4_β_1_ integrin and VCAM-1, and the released pro-inflammatory cytokines produce an inflammatory reaction that leads to neurodegeneration.^4,5^ In allergic conjunctivitis, α_4_β_1_ integrin mediates long-term infiltration of neutrophils, eosinophils, and T lymphocytes in the conjunctiva. This receptor participates in the pathogenesis of asthma and sarcoidosis, a disorder characterized by lymphocyte accumulation in the lung.^4,5^ Finally, several types of tumor cells express α_4_β_1_ integrin, and the interaction with VCAM-1 increases transendothelial migration and contributes to metastasis to distant organs.^4,5^ As for the related α_4_β_7_ integrin, its interaction with MAdCAM-1 is responsible for T lymphocytes homing to the gut.^4^

Consequently, targeting α_4_ integrins represents an opportunity for the treatment of inflammatory disorders,^1,2,4−6^ including allergic conjunctivitis,^7^ dry eye disease,^8^ AE,^9^ dry age-related macular degeneration,^10^ MS, and inflammatory bowel diseases, such as ulcerative colitis and Crohn’s disease.^11^

**Figure 1:**
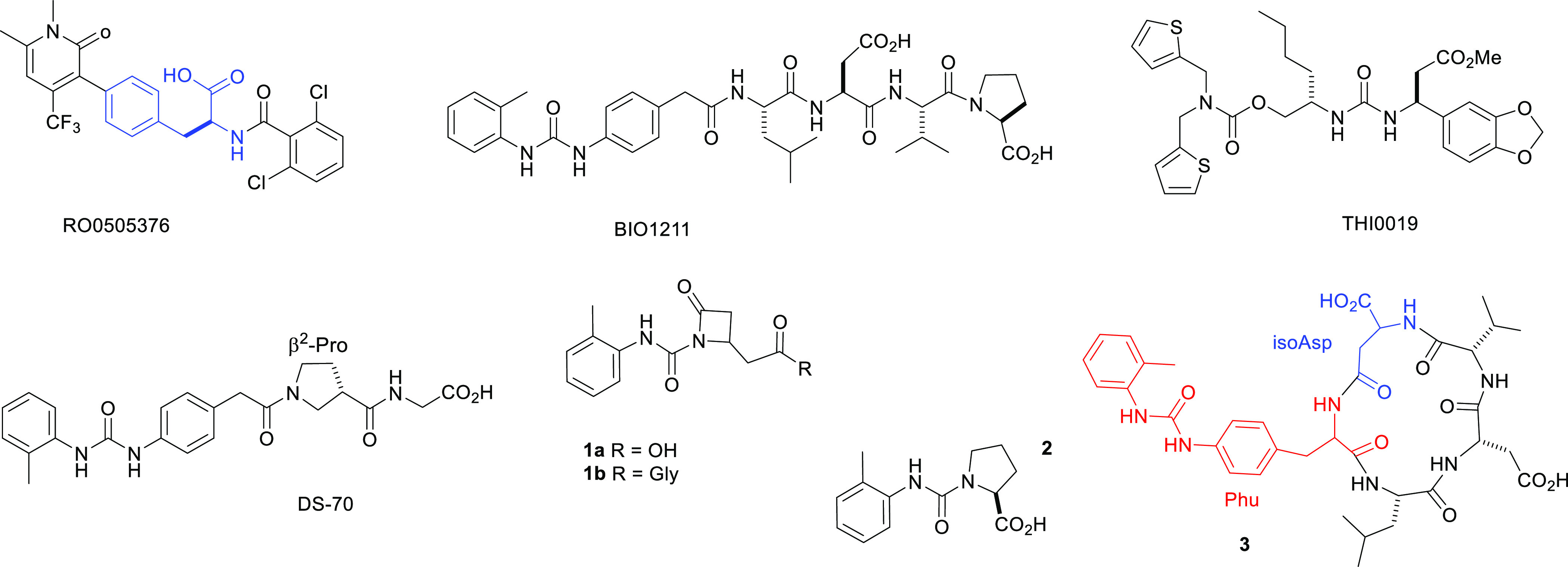
Structures of α_4_β_1_ integrin antagonists discussed in this paper: RO0505376, containing the phenylalanine nucleus (in blue); the LDV peptide-urea BIO1211; the mimetic DS-70. Structure of integrin agonists: the urea THI0019; the small urea derivatives **1** and **2**. Cyclic analogues of BIO1211 **3a**–**d**, including LDV and the phenylalanine-urea (Phu) residue (in red).

The small molecule antagonists of α_4_β_1_ integrin reported to date can be divided in two main classes, i.e. the *N*-acylphenylalanine derivatives, such as the compound RO0505376 (Figure 1), and the peptides derived from the LDV or IDS recognition motifs.^1,2^ Lin et al. found BIO1211 (Figure 1, Table 1), a LDVP peptide N-capped with the α_4_-targeting *o-*methylphenylureaphenylacetic acid (MPUPA) moiety,^12^ which inhibited antigen-induced airway hyperresponsiveness in allergic animals.^13^ Unfortunately, this peptide was found to be very unstable in heparinized blood, plasma, and rat liver, lung, and intestinal homogenates^14,15^ and to undergo rapid clearance in vivo.^16^

**Table 1:** Effect the LDV CPPs **3a**–**d**, **12a**, and BIO1211, on Integrin-Mediated Cell Adhesion (data are presented as IC_50_ for antagonists and as EC_50_ for agonists (nM))^a^

compd	sequence	purity (%)^b^	FN/Jurkat E6.1 α_4_β_1_	VCAM-1/Jurkat E6.1 α_4_β_1_	MAdCAM-1/RPMI8866 α_4_β_7_	Fg/HL60 α_M_β_2_	ICAM-1/Jurkat E6.1 α_L_β_2_	FN/K562 α_5_β_1_
BIO1211	MPUPA-LDVP-OH	-	5.5 ± 4.0^c^	4.6 ± 3.0^c^	nd	nd	8.4 ± 4.3^d^	>5000
			antagonist	antagonist			antagonist	
**3a**	c[(*S*)-Phu-LDV-(*S*)-isoAsp]	98	50.5 ± 7.8	35.0 ± 5.9	31.8 ± 5.5	>5000	98.2 ± 9.8	>5000
			agonist	agonist	agonist		agonist	
**3b**	c[(*S*)-Phu-LDV-(*R*)-isoAsp]	97	156 ± 33	81.8 ± 9.7	32.1 ± 5.3	>5000	1110 ± 340	>5000
			agonist	agonist	agonist		antagonist	
**3c**	c[(*R*)-Phu-LDV-(*S*)-isoAsp]	97	726 ± 28	177 ± 57	495 ± 89	>5000	710 ± 65	1950 ± 290
			antagonist	antagonist	antagonist		antagonist	agonist
**3d**	c[(*R*)-Phu-LDV-(*R*)-isoAsp]	99	40.9 ± 4.3	190 ± 30	>5000	353 ± 32	53.9 ± 5.1	>5000
			agonist	agonist		antagonist	antagonist	
**12a**	c[(*S*)-Phu-LAV-(*S*)-isoAsp]	95	55.6 ± 2.9	1.78 ± 0.32	>5000	53.4 ± 5.4	>5000	168 ± 61
			agonist	agonist		antagonist		agonist
**1a**^e^	nonpeptide, Figure 1	97	15.6 ± 1.5	13.0 ± 0.8	>5000	>5000	>5000	>5000
			agonist	agonist				
**1b**^e^	nonpeptide, Figure 1	96	>5000	>5000	>5000	>5000	>5000	9.7 ± 0.5
								agonist

a α_4_β_1_ integrin-mediated cell adhesion was evaluated by assaying Jurkat E6.1 cell adhesion to FN or to VCAM-1; for α_L_β_2_ integrin, Jurkat E6.1 cells to ICAM-1; α_5_β_1_ integrin, K562 cells to FN; α_M_β_2_ integrin, HL60 cells to fibrinogen (Fg); α_4_β_7_ integrin, RPMI8866 cells to MAdCAM-1. Values represent the mean ± SD of three independent experiments carried out in quadruplicate.

b Determined by RP HPLC performed on a C_18_ column 100 × 3 mm, 3 μm, 110 Å, mobile phase from 9:1 H_2_O/CH_3_CN/0.1% HCOOH to 2:8 H_2_O/CH_3_CN/0.1% HCOOH in 20 min, flow rate of 1.0 mL min^–1^ (General Methods).

c Reference (7).

d Reference (6).

e Compounds previously characterized as integrin agonists; see ref (34). nd: not determined.

To improve stability and bioavailability, effort was dedicated to design peptidomimetic analogues, in particular α/β hybrid peptides,^17−21^ also associated with the retrosequence strategy.^22^ For instance, the presence of a β^2^-Pro core in the antagonist DS-70 (Figure 1)^7^ conferred higher stability in mouse serum.

Besides the therapeutic applications, the use of selective integrin ligands was also exploited for diagnostic purposes.^23^ For example, nanostructured surfaces coated with LDV peptides^24^ or α/β hybrid peptides^25^ were able to reproduce the high-density multivalency binding between the integrin clusters and VCAM-1, showing high selectivity for α_4_β_1_ integrin-expressing Jurkat cells.

In contrast to the blockade of integrin functions, the activation of α_4_β_1_ integrin might represent an alternative strategy to perturb the progression of cell migration. Following integrin activation, deactivation is indispensable to allow leukocytes to roll on the endothelial surface. Hence, agonists can be utilized to prevent the release of adherent cells.^26^

The activation of α_4_β_1_ integrin could represent a promising therapeutic strategy in specific pathological conditions. It has been described that a small molecule α_4_β_1_ integrin agonist was able to improve cell retention and engraftment in stem cell-based therapies.^27^ In a mouse model of colon adenocarcinoma, a tumor-protective role of α_4_β_1_ was hypothesized: accelerated tumor growth was observed after α_4_β_1_ depletion, suggesting the possible use of small molecule agonists to therapeutically manipulate α_4_β_1_ expression level in cancer.^28^ Furthermore, both α_4_β_1_ and α_L_β_2_ have been implicated in the recruitment of anticancer CD8+ effector T cells to the tumor microenvironment; thus, a small agonist of both α_4_β_1_ and α_L_β_2_ increased the localization of cancer-specific T cells to the tumor, improving their antitumor action. This effect was further enhanced by coadministration of an anti-CTLA-4 therapy.^29^ The same small molecule α_4_β_1_ and α_L_β_2_ agonist 7HP349 has been proposed as an adjuvant of a DNA vaccine in a model of Chagas disease. This compound was able to enhance both prophylactic and therapeutic vaccine efficacy, showing the possibility to use an integrin agonist as an adjuvant to augment T cell-mediated immune response to different types of vaccines.^30^

As for other integrins expressed on leukocytes, recent findings established that α_M_ (CD11b) integrin plays a major role in modulating proinflammatory signaling pathways and it can represent an innovative therapeutic target. Accordingly, α_M_ allosteric agonists promoting the anti-inflammatory functions of α_M_ integrin, could be useful in the treatment of lupus nephritis, a debilitating and severe complication of systemic lupus erythematosus characterized by infiltration of immune cells to the kidneys.^31^ Moreover, α_M_β_2_ integrin agonists have also been suggested for the therapy of osteoarthritis; given that this integrin is involved in preventing chondrocyte hypertrophy and chondrocyte mineralization, activation of α_M_β_2_ with agonists could lead to reduced inflammatory response.^32^

Unfortunately, very few potent and selective α_4_β_1_ integrin agonists are currently available. The compound TBC3486, a selective integrin antagonist, was converted into the agonist THI0019 (Figure 1), a urea derivative which promoted cell retention and engraftment.^27^ The small ureas **1** and **2** are α_4_β_1_ integrin ligands and showed agonistic behavior (Figure 1).^33−35^ Very recently, a cyclic LDV peptide containing 4-amino-l-proline (Amp) and MPUPA was found to increase the adhesion of α_4_β_1_ integrin-expressing cells.^36^ As for other related integrins, Faridi et al. identified small agonists of integrin α_M_β_2_, an adhesive receptor expressed on many of the same leukocyte populations,^37−39^ while Yang et al. described the first small molecule agonist of the leukocyte integrin α_L_β_2_.^40^

In this context, we conceived a minilibrary of LDV α/β hybrid cyclopentapeptides (CPPs) **3** and related sequences (Figures 1 and 3). This approach yielded integrin agonists with diverse affinity for α_4_ integrins. Also, the CPPs were utilized as 3D probes for investigating the preferred bioactive conformations and to analyze α_4_β_1_ integrin binding. Indeed, because the three-dimensional structure of the integrin α_4_β_1_ is not yet available, at present the ligand’s structural determinants for agonism versus antagonism are not fully understood.

## Results and Discussion

### Synthesis of CPPs **3a**–**d**

**Scheme 1:**
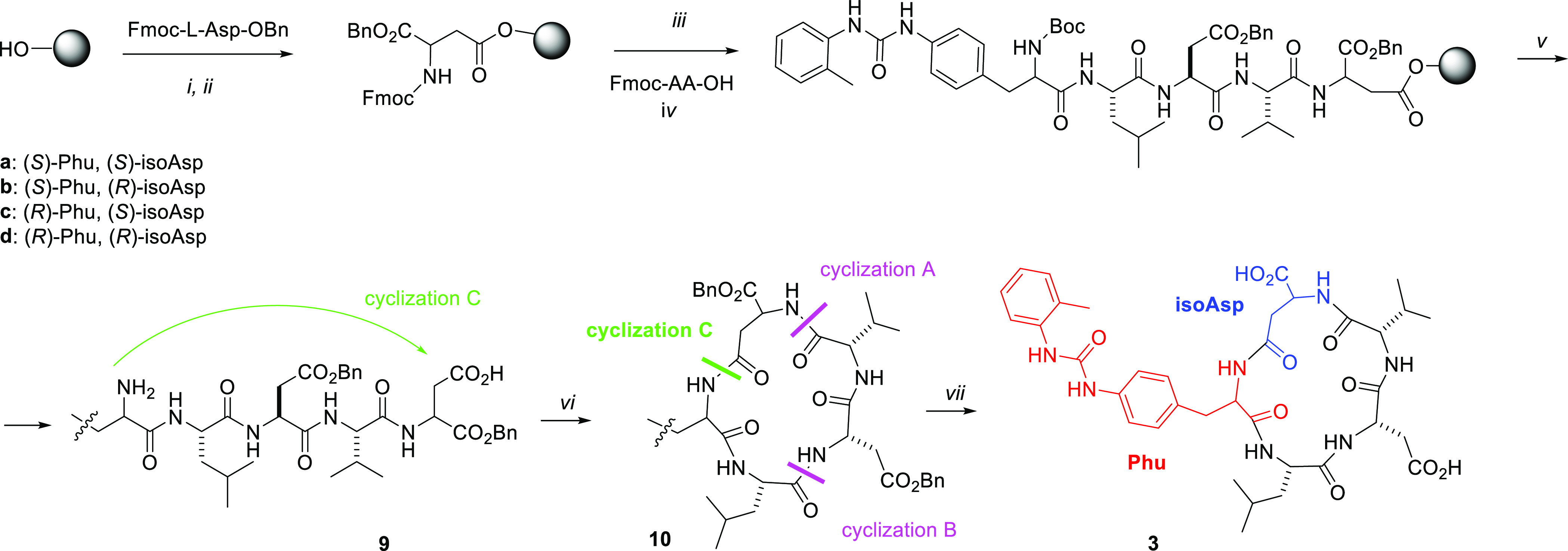
Synthesis of CPPs **3a**–**d** via Macrolactamization C Reagents and conditions: (i) Fmoc-l-Asp-OBn (3.0 equiv), DCC (3.0 equiv), HOBt (3.0 equiv), DMAP (0.1 equiv), DMF, RT, 3 h; (ii) capping: Ac_2_O (20 equiv), pyridine (20 equiv), RT, 30 min; (iii) 20% piperidine, DMF, RT, 10 min; (iv) Fmoc-AA-OH (2.0 equiv), DCC (2.0 equiv), HOBt (2.0 equiv), DMF, RT, 3 h; the last introduced residue was Boc-Phu-OH. (v) TFA/H_2_O/TIS (95/2.5/2.5), RT, 2.5 h; (vi) HBTU (3.0 equiv), HOBt (3.0 equiv), DIPEA (6.0 equiv), pseudo-high dilution in DMF, RT, 18 h; (vii) H_2_, Pd/C, RT, 12 h. Alternative routes A and B are also shown. For simplicity, part of Phu can be omitted.

To obtain LDV CPPs equipped with the MPUPA moiety, the diphenylurea moiety was anchored at the 4-position of (*S*)- or (*R*)-Phe, giving (*S*)- or (*R*)-*p*[3-(*o*-tolyl)urea]phenylalanine (Phu).^41^ The sequence was complemented with the β-amino acid (*S*)- or (*R*)-isoaspartate (isoAsp), to allow macrolactamization while maintaining a second carboxylic group, as at the C-terminus of BIO1211. In detail, isoAsp was introduced as (*S*)- or (*R*)-Fmoc-l-Asp-OBn, (*S*)- or (*R*)-**4**, and (*S*)- or (*R*)-Boc-Phu-OH (**8**) was prepared in-house (Supporting Information, Scheme S1).^42^ The CPPs of general structure c[(*S*/*R*)-Phu-Leu-Asp-Val-(*S*/*R*)-isoAsp] (**3a**–**d**) were prepared from linear precursors, obtained in turn by standard SPPS on Wang resin, with Fmoc-protected amino acids (Scheme 1).

The identification of the strategic amide bond for final head-to-tail cyclization was not trivial.^43^ Initially, we opted for the convenient cyclization A between the residues isoAsp and Val (Scheme 1). Hence, we prepared the all-l-configured H-isoAsp(OBn)-Phu-Leu-Asp(OBn)-Val-OH. Consistent with the results reported by Kessler and Marinelli for the cyclization of isoDGR peptides,^44^ the reaction gave poor yields of **10a** (Table S1). These authors observed that the presence of isoAsp strongly influenced the conformation of linear peptide precursors and recommended that cyclization could only be achieved if isoAsp was located in the middle of the sequence.

In contrast to the expectations,^44^ the cyclization between Asp and Leu (Scheme 1, cyclization B) gave a negligible yield (Table S1). Much better results were obtained for the ring-forming reaction between Phu and isoAsp (Scheme 1, ring closure C).

Hence, the sequences **9a**–**d** were prepared by standard Fmoc chemistry on a Wang resin (Scheme 1, Table S1). The crude **9a**–**d** (75–85% pure) were utilized for the macrolactamization step under pseudo-high-dilution conditions,^45^ giving **10a**–**d** (>95% pure after semipreparative RP HLPC). Final deprotection proceeded quantitatively affording the CPPs **3a**–**d** (96–98% pure, Table 1). The structures were confirmed by ESI-MS, ^1^H, ^13^C NMR, and 2D gCOSY spectroscopy.

### Integrin-Mediated Cell Adhesion Assay and Competitive Solid-Phase Binding Assay on Purified Integrins

In vitro experiments were carried out to detect any effects of **3a**–**d** on α_4_β_1_-mediated cell adhesion, and their selectivity toward α_4_β_7_, α_L_β_2_, and α_M_β_2_ integrins. Although not expressed on leukocytes, α_5_β_1_ integrin was also chosen, as it shares the β_1_ subunit with the heterodimer α_4_β_1_. Cells were seeded in 96-well plates coated with the specific natural human recombinant ligands (Table 1) and allowed to adhere in the presence of increasing concentrations (10^–10^ to 10^–4^ M) of the synthesized CPPs before the determination of the number of adherent cells (as described in Experimental Section).

**Figure 2:**
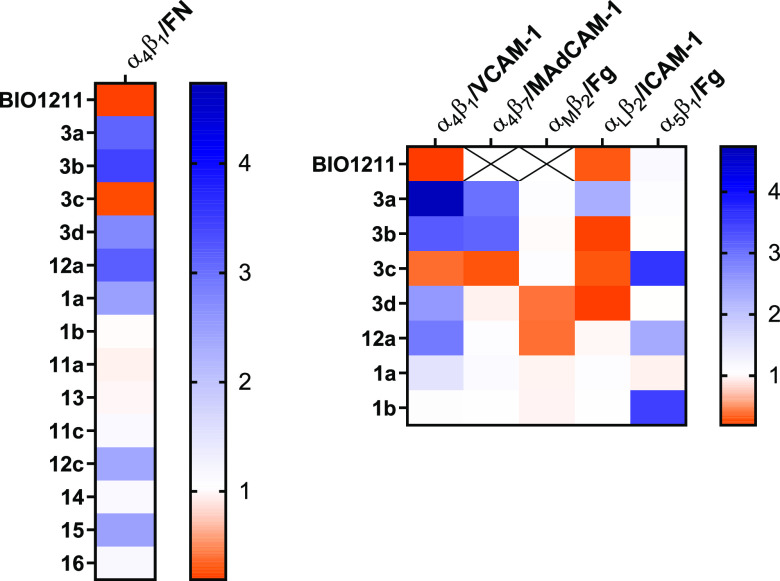
Heatmaps of adhesion index: agonist compounds are shown in shades of blue whereas antagonists are displayed in shades of orange. The adhesion index is calculated as the ratio between the number of adhered cells in the presence of the highest CPP concentration (10^–4^ M) and the number of adhered vehicle-treated cells. X: not determined.

The results of cell adhesion assays are summarized in Table 1 and Figure 2; the latter reports the heatmaps of adhesion index, a convenient illustration of agonistic or antagonistic behavior of the new synthesized compounds. On the basis of this parameter, an agonist is defined by adhesion index >1 (displayed in shades of blue), an antagonist by adhesion index <1 (displayed in shades of orange), and integrin ligands not significantly altering cell adhesion by adhesion index approximately = 1. In addition, concentration–response curves are provided in Supporting Information (Figures S2–S7).

To better characterize integrin–ligand interaction, competitive solid-phase ligand binding assays were performed on purified α_4_β_1_, α_4_β_7_, α_M_β_2_, α_L_β_2_, and α_5_β_1_ integrins, using receptor-specific ligands (Table 2) in the presence of increasing concentrations (10^–10^ to 10^–4^ M) of the CPPs.^34^

**Table 2:** Binding Affinities (IC_50_ values, nM)^a^ of LDV CPPs and BIO1211 on Purified Integrins

CPP	α_4_β_1_/FN	α_4_β_1_/VCAM-1	α_4_β_7_/MAdCAM-1	α_M_β_2_/Fg	α_L_β_2_/ICAM-1	α_5_β_1_/FN
BIO1211	8.6 ± 5.1^b^	8.9 ± 3.1	>1000	>1000	5.2 ± 2.1	>1000
**3a**	43.5 ± 3.5	33.5 ± 4.4	41 ± 7	>1000	83.3 ± 8.7	>1000
**3b**	133 ± 45	101 ± 35	22.7 ± 6.1	>1000	897 ± 230	>1000
**3c**	602 ± 32	707 ± 75	183 ± 22	>1000	652 ± 47	193 ± 65
**3d**	38.2 ± 8.1	28.5 ± 3.1	>1000	244 ± 71	46.7 ± 7.9	>1000
**12a**	46.1 ± 4.7	41.1 ± 3.1	>1000	47.2 ± 3.1	>1000	203 ± 43
**12c**	1567 ± 344	>5000	>5000	>5000	>5000	>5000
**15**	976 ± 168	899 ± 198	>5000	>5000	>5000	>5000
**1a**^c^	13.3 ± 6.3	10.1 ± 4.9	>5000	>5000	>5000	>5000
**1b**^c^	>5000	>5000	>5000	>5000	>5000	49 ± 7

a IC_50_ values for α_4_β_1_, α_4_β_7_, α_M_β_2_, α_L_β_2_ and α_5_β_1_ integrins were determined by a competitive solid-phase binding assay to specific ligand (FN for α_5_β_1_, VCAM-1 or FN for α_4_β_1_, fibrinogen for α_M_β_2_, MAdCAM-1 for α_4_β_7_ and ICAM-1 for α_L_β_2_).

b Mean ± SD of three independent experiments carried out in triplicate.

c Compounds previously characterized as integrin agonists, ref (34).

In the cell adhesion experiments, no significant cell adhesion was observed for bovine serum albumin (BSA)-coated plates (negative control). The reference antagonist BIO1211 inhibited the adhesion of α_4_β_1_ integrin-expressing Jurkat E6.1 cells to FN and VCAM-1 (IC_50_ 5.5 nM and 4.6 nM, respectively, Table 1). In the competitive binding assay on purified α_4_β_1_ integrin, BIO1211 confirmed a low nanomolar affinity as reported in the literature (Table 2).^7^

Moreover, previously synthesized and characterized integrin agonists **1a** and **1b** were employed as reference ligands; these compounds were able to increase α_4_β_1_- or α_5_β_1_-mediated cell adhesion, respectively.^34^ As expected, only **1a** increased the adhesion of Jurkat E6.1 cells (Table 1), with high affinity toward the isolated integrin (Table 2), while **1b** was completely ineffective. Regarding the CPPs, cell adhesion experiments revealed compounds capable to reduce the number of adherent cells promoted by the natural ligands, referred to as antagonists, whereas other ligands increased cell adhesion and therefore were considered to be agonists (Figure 2).

The CPPs **3a**, **3b**, and **3d** were able to increase cell adhesion in a concentration-dependent manner (Table 1). Remarkably, **3a** showed potency in the nanomolar range (EC_50_/VCAM-1 35 × 10^–9^ M, IC_50_/FN 50.5 × 10^–9^ M), while **3b** and **3d** displayed a comparatively lower activity (**3b**, EC_50_/VCAM-1 81.8 × 10^–9^ M, EC_50_/FN 156 × 10^–9^ M; **3d**, EC_50_/VCAM-1 190 × 10^–9^ M, EC_50_/FN 40.9 × 10^–9^ M), Notably, **3c** was found to be an antagonist with moderate potency (IC_50_/VCAM-1 177 × 10^–9^ M, and IC_50_/FN 726 × 10^–9^ M).

These results were confirmed by α_4_β_1_ affinity evaluation in competitive solid-phase ligand binding: **3a** and **3d** displayed nanomolar IC_50_ values whereas **3b** and **3c** showed a lower affinity for α_4_β_1_ (Table 2). Binding curves are provided in Supporting Information (Figures S8–S12).

To determine the extent to which experimentally determined binding affinity of CPPs correlates with their potency in modulating integrin-mediated cell adhesion, the Pearson (*r*_P_) correlation coefficient was calculated. As regards to α_4_β_1_, there was a high positive correlation between binding affinity and FN-mediated cell adhesion potency for all compounds tested (*r*_P_ = 0.9990, Figure S13), meaning that the highest is the affinity for α_4_β_1_ and the highest is the potency in cell adhesion assays.

Notably, regarding the correlation between binding affinity and VCAM-1-mediated cell adhesion potency, a quite low correlation coefficient was determined (*r*_P_ = 0.5920, Figure S13); for most compounds a correlation was found, but some exceptions were identified as those CPPs with the lowest potency for α_4_β_1_/VCAM-1 (**3c** and **3d**).

Cell adhesion assays on different integrin-expressing cell lines were also performed to determine compound selectivity (Table 1 and Figure 2). Nanomolar agonist activity was observed in adhesion experiments with RPMI8866 cells expressing α_4_β_7_ integrin to the ligand MAdCAM**-**1 for the compounds **3a** (EC_50_ 31.8 × 10^–9^ M) and **3b** (EC_50_ 32.1 × 10^–9^ M). Ligand binding assays on purified α_4_β_7_ integrin confirmed excellent affinity of **3a** and **3b** (Table 2). Therefore, they were considered α_4_β_1_/α_4_β_7_ integrin dual agonists. On the other hand, **3c** was found to be a dual, moderate antagonist of α_4_β_1_/α_4_β_7_ integrins (for α_4_β_7_ integrin, IC_50_ 4.95 × 10^–7^ M) with a lower affinity (Table 2). The reference compounds BIO1211 and **1a** were found to be inactive in the same assays (Table 1, and 2), as reported.

In the tests for α_L_β_2_ integrin, the reference BIO1211 and the CPPs **3b**, **3c**, and **3d**, behaved as antagonists with diverse potency in cell adhesion experiments, the most potent among the CPPs being **3d** (IC_50_ 53.9 × 10^–9^ M) (Table 1). In contrast, **3a** was identified as a potent α_L_β_2_ integrin agonist (EC_50_ 98.2 × 10^–9^ M, Table 1). Regarding affinities for isolated α_L_β_2_ integrin, BIO1211, **3a**, and **3c** showed excellent affinity values, while **3b** and **3d** were able to bind α_L_β_2_ integrin with modest affinity (Table 2). As described for α_4_β_1_, a very high positive correlation between ligand binding affinity and cell adhesion potency was observed also for α_L_β_2_, the correlation index being 0.9969 (Figure S13). This means that compounds with a low potency toward α_L_β_2_ are able to bind it with a low affinity and vice versa. Concerning α_M_β_2_ integrin, the only modestly active compound able to bind to α_M_β_2_ was the antagonist **3d** (IC_50_ 3.53 × 10^–7^ M, Tables 1 and 2).

Finally, while **3c** showed a scarce but measurable agonistic activity toward α_5_β_1_ integrin (EC_50_ 1.95 × 10^–6^ M), BIO1211, **3a**, **3d**, and **3b** were found to be inactive (Table 1) and not able to bind to isolated α_5_β_1_ integrin (Table 2). Not unexpectedly, **1b** was a potent agonist of this integrin with nanomolar affinity (Tables 1 and 2).

### Synthesis of CPPs **11a**,**c**, **12a**,**c**, and **13**–**16**

**Figure 3:**
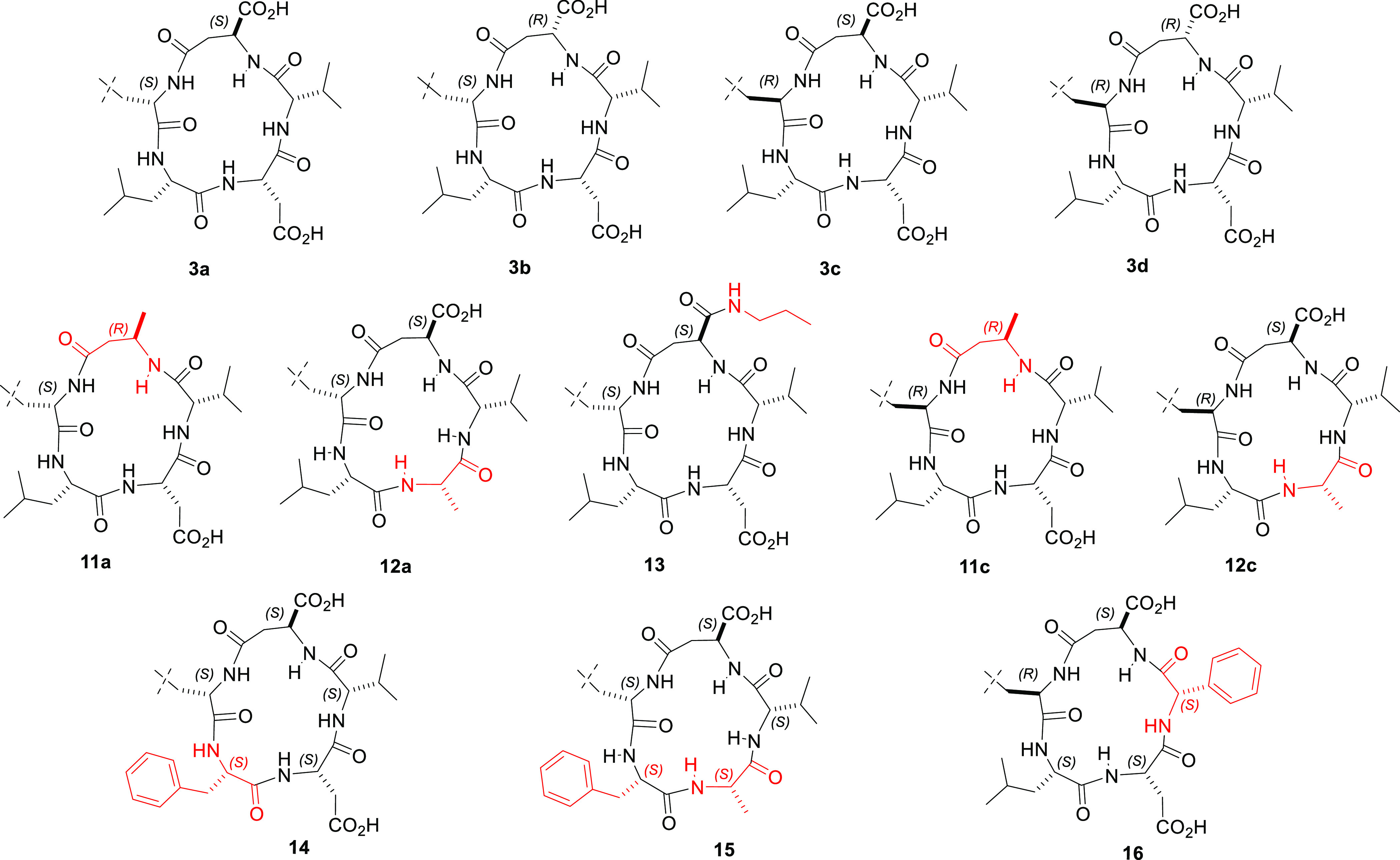
Sketches of the CPPs **3a**–**d**, and the related **11a**,**c**, **12a**,**c**, and **13**–**16**; part of the Phu residue has been omitted. The CPPs **11a**, **12a**, and **13**–**15** maintain the same topology of **3a**, i.e. the same 3D display of each residue’s side chain, while **11c**, **12c**, and **16** maintain the topology of **3c**; the mutated residues are shown in red. For simplicity, part of Phu is omitted.

To better distinguish the pharmacodynamic role of the two carboxylate groups and of some relevant side chains in receptor binding and in determining agonism or antagonism behavior, the most potent agonist **3a** and the antagonist **3c** were selected for modifications. CPP **3a** was modified either by replacing isoAsp^5^ with (*R*)-β^3^-homoAla, giving c[(*S*)-Phu-LDV-(*R*)-βAla^5^] (**11a**), or by replacing Asp^3^ with Ala, giving the peptide c[(*S*)-Phu-LAV-(*S*)-isoAsp^5^] (**12a**). Topologically, the (*R*) configuration of β^3^-homoAla corresponds to the (*S*) configuration of isoAsp (Figure 3). Alternatively, the isoAsp^5^ carboxylate side chain in **3a** was derivatized to the corresponding propylamide, giving **13**, or the Leu^2^ in **3a** was replaced with aromatic Phe, yielding **14**. Peptide **15** was further modified based on **12a** by replacing Leu^2^ with Phe.

Similarly, the structure of **3c** was modified by replacing isoAsp^5^ with (*R*)-β^3^-homoAla, giving c[(*R*)-Phu-LDV-(*R*)-βAla^5^] (**11c**), or Asp^3^ was replaced with Ala, giving c[(*R*)-Phu-LAV-(*S*)-isoAsp] (**12c**). The (*R*) configuration of β^3^-homoAla corresponds to the (*S*) configuration of isoAsp (Figure 3). Alternatively, the Val^4^ in parent **3c** was substituted with an aromatic Phenylglycine (Phg), yielding **16**.

The CPPs were prepared from the linear precursors **9e**–**l** (Table S1) as reported for **3a**–**d**. To this purpose, Fmoc-(*R*)-β^3^homoAla-OH **20** was synthesized by adapting a procedure reported in the literature (Supporting Information);^46^ Fmoc-Asp-propylamide **21** was readily prepared from Fmoc-(*R*)-Asp(OtBu)-OH and *n*-propylamine (Scheme S2). Cyclization under pseudo-high dilution conditions afforded **10d**–**l** (Table S1); the CPPs **11a**,**c**, **12a**,**c**, and **13**–**16** were obtained after final deprotection (>95% pure, Table 3).

**Table 3:** Effect of Cyclic Peptides **11a**,**c**, **12a**,**c**, and **13**–**16** on Jurkat E6.1 Cell Adhesion to FN, Presented as IC_50_ for Antagonists and as EC_50_ for Agonists (nM)^a^

CPP	sequence	purity (%)^b^	FN/Jurkat E6.1 α_4_β_1_
**11a**	c[(*S*)-Phu-LDV-(*R*)-β^3^Ala]	97	>5000
**11c**	c[(*R*)-Phu-LDV-(*R*)-β^3^Ala]	98	>5000
**12a**	c[(*S*)-Phu-LAV-(*S*)-isoAsp]	95	55.6 ± 2.9
			agonist
**12c**	c[(*R*)-Phu-LAV-(*S*)-isoAsp]	97	1720 ± 556
			agonist
**13**	c[(*S*)-Phu-LDV-(*S*)-isoAsp(NH*Pr*)]	98	>5000
**14**	c[(*S*)-Phu-FDV-(*S*)-isoAsp]	97	>5000
**15**	c[(*S*)-Phu-FAV-(*S*)-isoAsp]	98	1061 ± 134
			agonist
**16**	c[(*R*)-Phu-LD-Phg-(*S*)-isoAsp]	96	>5000

a Mean ± SD of three independent experiments carried out in quadruplicate.

b Determined by analytical RP HPLC performed on a C18 column (see footnote to Table 1 and General Methods).

### α_4_β_1_ Integrin-Mediated Cell Adhesion Assay of **11a**,**c**, **12a**,**c**, and **13**–**16** and Competitive Binding Assay on Purified Integrins

The effects of the new CPPs derived from **3a** and **3c** on the adhesion of α_4_β_1_ integrin-expressing Jurkat E6.1 cells to the ligand FN were assayed as discussed above (Table 3 and Figure 2). Apparently, the replacement of the isoAsp^5^ with β^3^-homoAla in both **3a** and **3c** was not tolerated for activity toward α_4_β_1_ integrins, because **11a** and **11c** became inactive in the Jurkat E6.1 cell adhesion assay (IC_50_ > 5000 nM, Table 3). In a similar way, the derivatization of isoAsp^5^ carboxylate into the amide in peptide **13** led to a complete loss of activity (Table 3).

In contrast, the substitution of Asp^3^ by introduction of Ala to give **12a**,**c** was much better tolerated, albeit **12c** showed a decrease of activity as compared to the parent **3c** (EC_50_ 1.72 × 10^–6^ M vs 7.26 × 10^–7^ M). Similar results were confirmed by binding affinity toward purified α_4_β_1_ integrin (Table 2). Furthermore, the moderate antagonist behavior of **3c** was converted to agonism in **12c** (see also Computational Studies and Supporting Information). Intriguingly, CPP **12a** maintained the nanomolar agonist activity of the parent **3a** (EC_50_ 55.6 × 10^–9^ M) and excellent binding affinity (Table 2).

The CPPs **14**–**16** showed very modest or null activity in the cell adhesion assay (Table 3), with only **15** giving a measurable increase of cell adhesion (EC_50_ 1.72 μM) and micromolar affinity for the isolated receptor (Table 2), confirming the importance of Leu and Val.

Further cell adhesion assays using cell lines expressing different integrins and competitive solid-phase binding assays on purified integrins were performed to better characterize the activity of **12a** (Table 1), while the other CPPs were neglected, for the scarce to null activity toward α_4_β_1_ integrins. CPP **12a** showed significantly improved potency as compared to **3a** in the adhesion of Jurkat E6.1 cells to VCAM-1, with an outstanding EC_50_ 1.78 × 10^–9^ M (Table 1). Notably, while **3a** was a dual agonist of α_4_β_1_/α_4_β_7_ integrins with similar potency (Table 1), **12a** completely lost activity and binding ability for α_4_β_7_ integrin (Tables 1 and 2, see also Computational Studies and Supporting Information, Figure S18). On the other hand, **12a** was inactive toward α_L_β_2_ integrin (Tables 1 and 2), while becoming a modest agonist for α_5_β_1_ integrin (EC_50_ 1.68 × 10^–7^ M, Table 1), with affinity in the submicromolar range for the isolated integrin (Table 2). Finally, **12a** was able to bind to and activate α_M_β_2_ integrin as an antagonist, with an interesting IC_50_ in the nanomolar range (IC_50_ 53.4 × 10^–9^ M, Table 1) and noteworthy nanomolar affinity (Table 2).

### Effects of the CPPs on Integrin-Mediated Intracellular Signaling

To confirm the agonist or antagonist behavior, the effect of the reference compound BIO1211, **3a**–**d**, and **12a** on phosphorylation of ERK1/2 in Jurkat E6.1 cells was determined. Intracellular signaling generated by the interaction of ECM components with α_4_β_1_ integrin produces an increase in the phosphorylation of cytoplasmatic second messengers such as ERK1/2 that contribute to α_4_ integrin-mediated cell functions.

**Figure 4:**
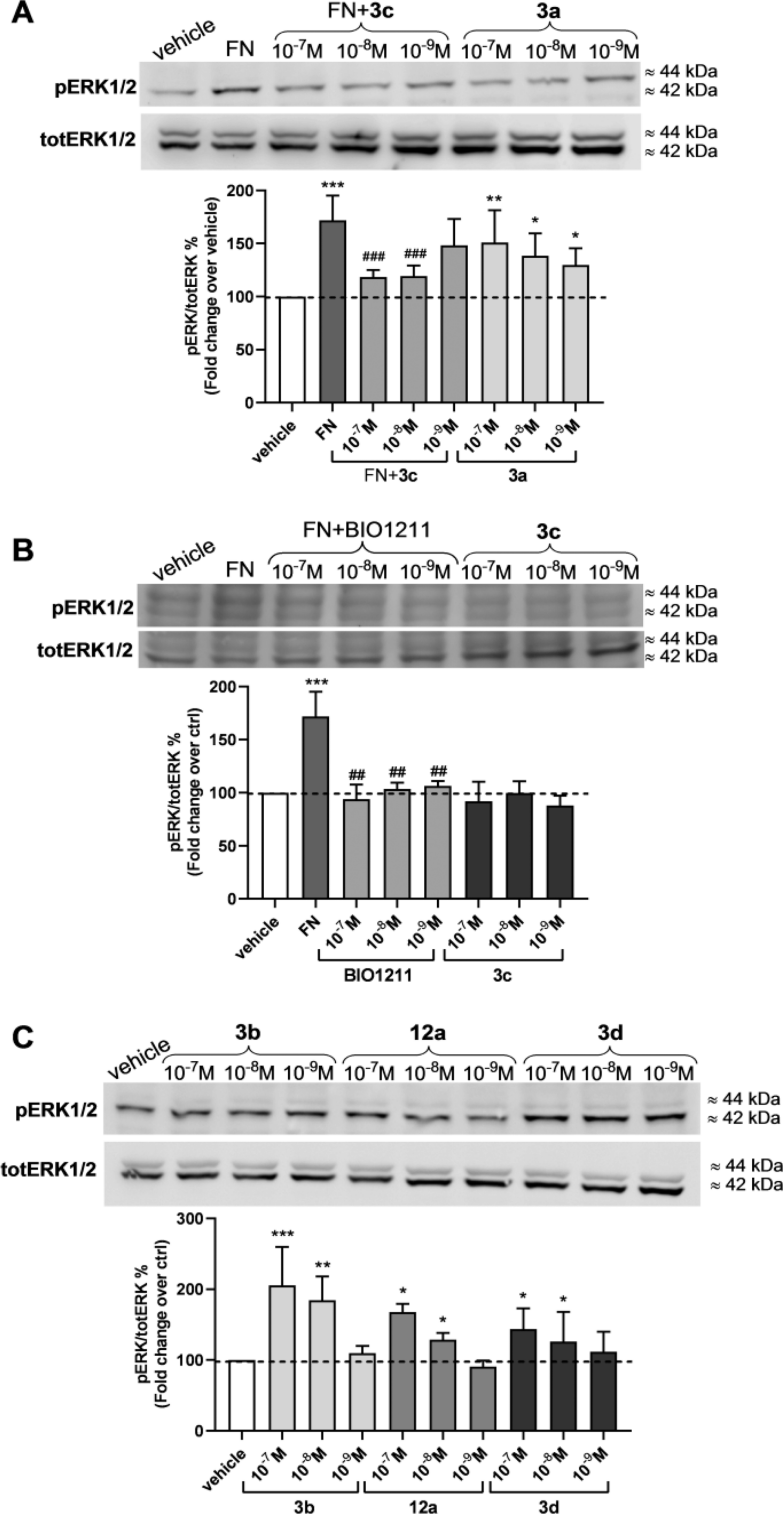
Effects of FN (10 μg/mL), the reference compound BIO1211, **3a**–**d**, and **12a** (10^–7^ to 10^–9^ M) on ERK1/2 phosphorylation mediated by α_4_β_1_ integrin expressed on Jurkat E6.1 cells. (A, B) The antagonists BIO1211 and **3c** were able to prevent ERK1/2 phosphorylation induced by FN. The antagonist **3c**, administered alone to Jurkat E6.1 cells, did not modify phosphorylation levels of ERK1/2. On the contrary, the agonists **3a** (A) and **3b**, **3d**, and **12a** (C) induced ERK1/2 activation in a concentration-dependent manner. Representative Western blot shows that Jurkat E6.1 cells plated on FN had a signal for pERK1/2 stronger than that for vehicle-treated cells (vehicle). The graphs represent densitometric analysis of the bands (mean ± SD; three independent experiments); the amount of pERK1/2 is normalized to that of totERK1/2. *p < 0.05, **p < 0.01, ***p < 0.001 vs vehicle; ^##^*p* < 0.01, ^###^*p* < 0.001 vs FN (Newman–Keuls test after ANOVA).

The endogenous ligand FN (10 μg/μL), employed as positive control, induced a significant increment of ERK1/2 phosphorylation in comparison to vehicle-treated Jurkat E6.1 cells (Figure 4A). The reference compound BIO1211 (10^–7^ to 10^–9^ M), which is defined as an α_4_ integrin antagonist, significantly prevented ERK1/2 activation induced by FN (Figure 4B).

Similarly to BIO1211, the CPP **3c** (10^–7^ to 10^–9^ M) significantly reduced FN-induced intracellular signaling activation, confirming action as an antagonist (Figure 4A). To further confirm the antagonist behavior, **3c** was administered alone to Jurkat E6.1 cells. In this experimental setting, **3c** did not influence ERK1/2 activation (Figure 4B), thus probably binding to α_4_β_1_ without inducing its activation and the resulting downstream intracellular signaling. In contrast, a significant concentration-dependent increase of ERK1/2 phosphorylation was produced by the α_4_β_1_ agonists **3a** (Figure 4A) and **3b**, **3d**, and **12a** (Figure 4C), confirming their ability to bind the receptor and to induce its activation.

### In Vitro Enzymatic Stability of **3a**,**c**

To estimate any increase in enzymatic stability conferred by the α/β hybrid cyclic structure,^18^ the representative **3a** and **3c** were incubated in mouse serum in comparison to the reference antagonist BIO1211 (Supporting Information, Figure S1). Consistent with other studies,^14,15^ BIO1211 was found to be poorly stable when added to mouse serum, being almost completely hydrolyzed after 2 h, as determined by RP HPLC analysis. In contrast, **3a** and **3c** appeared significantly more stable, and after 3 h the remaining amount was estimated at >85%.

### Conformational Analysis of the CPPs

Apparently, the LDV CPPs **3a**–**d** showed diverse integrin affinity and cell adhesion effects to ligand-coated plates, albeit differing only by the absolute configuration of the residues Phu and/or isoAsp. This suggested that the overall geometry exerts a clear impact on ligand–receptor interactions and binding. Hence, we analyzed the 3D conformations of **3a**–**d** in solution by NMR spectroscopy and molecular dynamics (MD) simulations.

The NMR analysis was conducted in 8:2 mixtures of DMSO-*d*_6_/H_2_O, a highly viscous solvent system recommended as an excellent biomimetic environment.^47,48^ For each peptide, ^1^H NMR spectra showed a single set of resonances, indicating conformational homogeneity or a rapid interconversion between the conformers. gCOSY analyses allowed the unambiguous assignment of the resonances. Variable temperature (VT) ^1^H NMR experiments were used to determine if the amide protons were plausibly involved in intramolecular hydrogen bonding or were solvent exposed (Table S2).^49^

**Figure 5:**
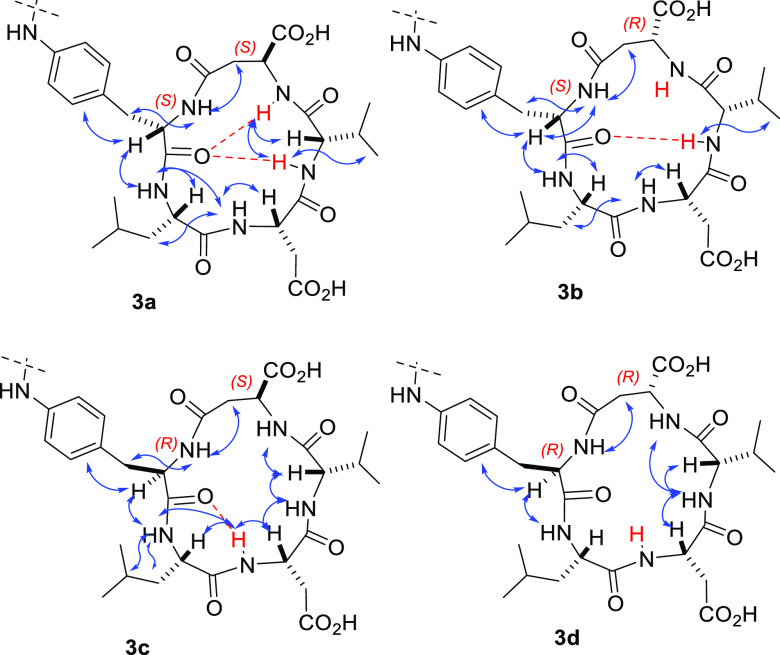
Sketches of the structures of the cyclic LDV peptides **3a**–**d** showing meaningful proton–proton correlations indicated by arrows, as determined by 2D ROESY in DMSO-*d*_6_/H_2_O. The amide protons characterized by low |Δδ/Δ*T*| values (Table S2) are shown in red; predicted hydrogen bonds are shown as red dashed lines. For clarity, part of the diphenylurea moiety has been omitted.

The analyses of the experimental Δδ/Δ*T* (ppb K^–1^) parameters (Supporting Information, Table S2) suggest the occurrence of strong hydrogen bonds for Val^4^NH and isoAsp^5^NH in **3a**, while a strong hydrogen bond was supposed for Asp^3^NH in **3c** and **3d** (Figure 5). Full details are given in Supporting Information.

2D ROESY analyses were performed in the same solvent system. Cross-peak intensities were ranked to infer plausible interproton distances (Figure 5, and Supporting Information, Tables S3–S7). The estimated distances were analyzed by simulated annealing and restrained MD simulations, using the AMBER force field^50^ in explicit water. In brief, random geometries of each peptide were sampled during a high-temperature unrestrained MD simulation in a box of TIP3P models of equilibrated water molecules.^51^ For each random structure, the interproton distances deduced by ROESY were introduced as constraints. As the absence of Hα(*i*)-Hα(*i*+1) cross-peaks reasonably excludes the occurrence of cis*-*peptide bonds, the amide bonds angles (ω) were set at 180°.

The structures were subjected to restrained high-temperature simulation with a scaled force field, followed by a period with full restraints, and then the system was slowly cooled. The resulting structures were minimized, and the backbones of the structures were clustered by rmsd analysis. For all compounds, this procedure gave one major cluster comprising the large majority of the structures.

**Figure 6:**
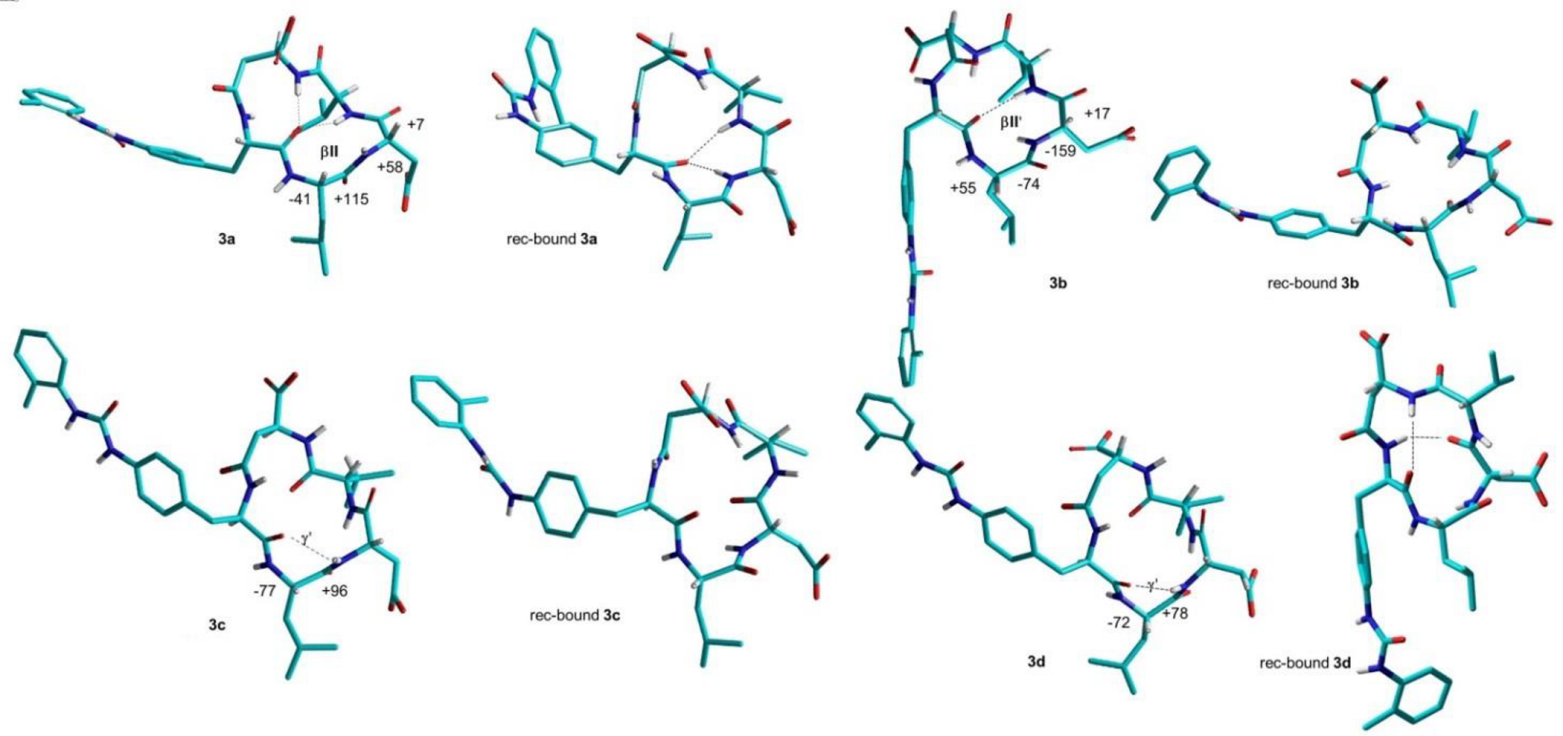
Representative lowest energy structures for the cyclic LDV peptides **3a**–**d**, calculated by ROESY-restrained MD in a 30 × 30 × 30 Å box of standard TIP3P water molecules. The receptor-bound poses as predicted by molecular docking (see next paragraph) are also shown for comparison. Hydrogen bonds are shown as dotted lines.

The representative structures with the lowest energy and the least number of restraint violations were selected and analyzed. The ROESY-derived structures of **3a** and **3b** (Figure 6) show explicit hydrogen bonds as predicted by VT-NMR analysis. Peptide **3a** is characterized by a clear type II β-turn (βII) centered on Leu^2^-Asp^3^. In **3b**, Leu^2^-Asp^3^ appeared to be embedded within an inverse type II β-turn (βII′), plausibly due to the reversal of stereochemistry of the β-residue^5^. The structures of **3c** and **3d** show similar overall geometries, each showing an inverse γ-turn (γ′) centered on Leu^2^.

To investigate the dynamic behavior of the LDV CPPs, the structures were analyzed by unrestrained MD simulations at 298 K in a box of explicit TIP3P equilibrated water molecules. During the simulations, the structures of the backbones were maintained, indicating that these conformations plausibly represented stable minima (not shown).

The secondary structure elements observed for the α/β hybrid **3a**–**d** were foreseeable; indeed, β-amino acids are well-known to favor defined secondary structures when introduced in CPPs.^52^ These residues exert a significant conformational bias on backbone conformations and preferably adopt a pseudo-γ-turn at the central position and tend to stabilize γ-turn secondary structures at the opposite side of the macrocycle.^53^

As for the other related CPPs, a comparison of the ^1^H NMR spectra supports that **11a** and **13**–**15** maintain conformations similar to that of the parent compound **3a**, because the chemical shifts of the resonances for the unaltered residues were practically the same (Supporting Information, Figures S22 and S33). Similarly, the compounds **11c**, **12c**, and **16** showed NMR spectra comparable to that of the parent compound **3c**. VT-NMR analysis showed for all CPPs the same trends of Δδ/Δ*T* parameters, suggesting that the hydrogen-bonding patterns and secondary structure elements were maintained (Table S2).

**Figure 7:**
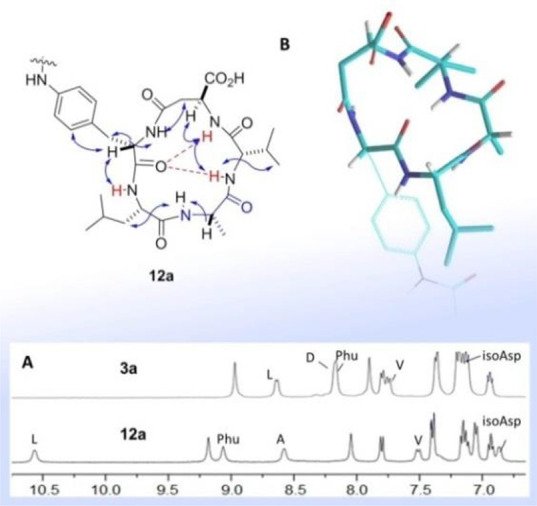
Conformational analysis of **12a**. Meaningful proton–proton ROESY correlations are indicated by arrows; the amide protons characterized by low |Δδ/Δ*T*| values are shown in red; hydrogen bonds are shown as red dashed lines. (A) Comparison of the amide-NH regions for **3a** and **12a**. (B) Representative lowest energy structure for **12a**, calculated by ROESY-restrained MD in a 30 × 30 × 30 Å box of standard TIP3P water molecules; the MPUPA moiety is rendered in sticks for clarity.

Unexpectedly, **12a** displayed differences with respect to **3a** in the ^1^H NMR spectra relative to the resonances of Leu and Phu (δ = 9.1 and 10.6 ppm, respectively). In particular, Phu^1^NH and Leu^2^NH in **12a** appeared downfield (δ = 9.1 and 10.6 ppm, respectively), as compared to the parent peptide **3a**. (Figure 7A). Furthermore, VT-NMR analysis (Table S2) showed for Leu^2^NH an atypical positive Δδ/Δ*T* (+1.6 ppb K^–1^). As a consequence of the NMR evidence, the structures of **11a**,**c**, **12c**, and **13**–**16** were not investigated further, while the 3D structure of **12a** in solution was analyzed by 2D ROESY analysis and restrained MD, as reported for **3a**–**d**. Eventually, this procedure confirmed that **12a** still maintains the same conformation of **3a** (Figure 6 vs Figure 7B). Possibly, the diverse chemical fields for Phu-Leu resonances might be due to peculiar deshielding effects exerted, e.g. by the urea group, rather than to the occurrence of different overall 3D geometries.

### Computational Studies

The mechanism by which an agonist such as **3a** is able to increase, while the antagonist **3c** decreases the adhesion of the receptor to the native ligands, appears particularly puzzling. Very few studies have been dedicated to leukocyte integrin agonists.^36^ Previously, Faridi et al. analyzed the interaction of small α_M_β_2_ agonists by molecular docking. The simulations suggested that the ligands recognize a hydrophobic cleft next to the ligand-binding site, implying an allosteric mechanism.^37^

Another agonist analyzed by molecular docking was the urea THI0019, capable to enhance the adhesion of cultured cell lines expressing α_4_β_1_ integrin to the ligands VCAM-1 and the CS-1 region of FN. Docking of this agonist into the available α_4_β_7_ crystal structure indicated that the ligand binds at a site that overlaps the ligand binding pocket.^27^ Thus, the authors hypothesized that the compound would have to be displaced from this site upon natural ligand binding. While such a ligand swap makes sense for a low affinity agonist such as THI0019 (IC_50_ in the 1–2 mM range), for the agonist **3a**, which shows a nanomolar IC_50_, another model must be considered.

To investigate the structural elements at the basis of the agonist or antagonist behavior, molecular modeling of the prototypic **3a** and **3c** was performed with Autodock 4.0.^54^ In addition, the analysis was extended to the stereoisomers **3b** and **3d** and to the derivatives **12a**,**c** and **15**. These CPPs have been selected for their at least measurable affinity for isolated integrin and clear effects on the adhesion of Jurkat E6.1 cells to the natural ligand (Tables 1–3).

Simulations of α_4_β_1_ integrin are particularly challenging because the precise structure of this integrin is not yet available. In addition, molecular mechanics force fields generally utilized to analyze ligand–receptor interactions are lacking in descriptions of the highly directional nature of metal coordination. For this reason, the region containing the ligand MIDAS and the receptor residues in the binding site were treated by hybrid density-functional theory (DFT) combined QM/MM calculation. The α_4_β_1_ integrin receptor model (Supporting Information, Figure S16) was obtained by combining the crystal structures of the α_4_ subunit (PDB ID: 3V4V, crystal structure of α_4_β_7_ headpiece complexed with Fab ACT-1 and RO0505376)^55^ and of the β_1_ subunit (PDB ID: 4WK4, metal ion and ligand binding of integrin).^56^

The receptor is expected to coordinate a carboxylic group of the ligands through the Mg^2+^ ion of the metal ion-dependent adhesion site (MIDAS) in the αI and βI domains.^55,56^ Other metal ion binding sites close to MIDAS are present, i.e. Ca^2+^ ions coordinated by residues in the adjacent to MIDAS site (ADMIDAS), and a synergistic metal ion binding site (SyMBS), in the βI domains. SyMBS and ADMIDAS have important roles in regulating ligand binding affinity. In β_1_ integrins, the ADMIDAS seems to be a negative regulatory site responsible for integrin inhibition by high concentration of Ca^2+^ and for activation by Mn^2+^.^56^

In the resulting model of α_4_β_1_ integrin, the plausible ligand-binding pocket appears characterized by a long binding groove at the α/β interface, as reported for α_4_β_7_ integrin (Figure S14).^55^ This shape is clearly different from that of the Arg-Gly-Asp (RGD)-binding integrins α_v_β_3_^57^ or α_IIb_β_3_.^58^ In particular, the α_4_ subunit is completely lacking in the cavity deputed to hosts Arg. Furthermore, the comparison between the β_1_ and β_3_ subunits reveals that the former contributes to expand the binding pocket of α_4_β_1_ integrin on the β subunit side, because the residues Arg^214^ and Arg^216^ in β_3_ subunit are replaced with Gly^217^ and Leu^219^ in β_1_.

**Figure 8:**
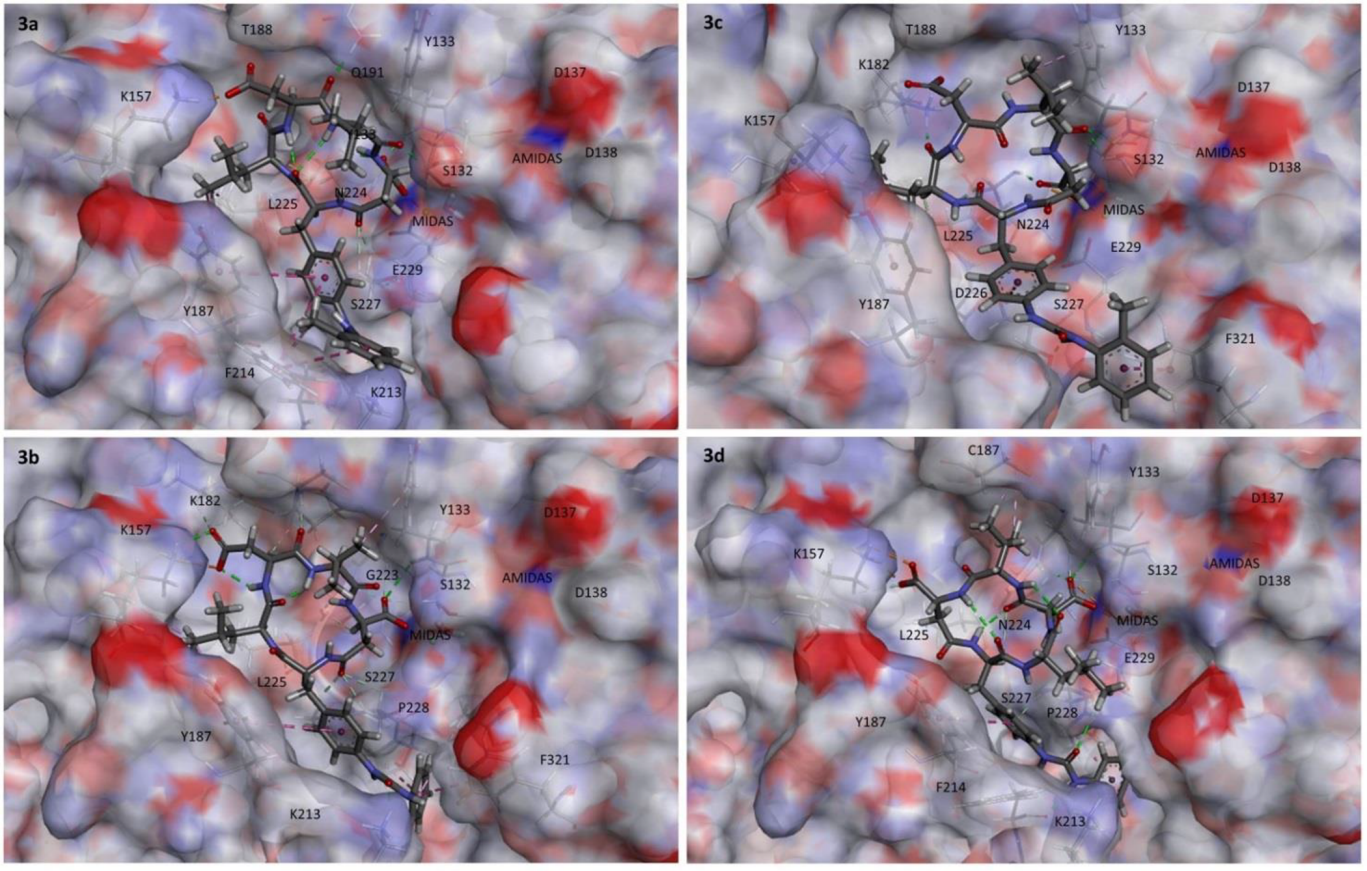
Calculated binding conformations of **3a**–**d** (right) within the α_4_β_1_ integrin binding site. Ligands are rendered in stick and colored by atoms. The integrin binding site is represented by its partially transparent, solid solvent-accessible surface, colored by the atomic interpolated charge. Key receptor residues are represented in tiny sticks, and nonbonding interactions are indicated as dashed lines. Images were obtained using BIOVIA DSV2021.

The best binding conformations of **3a** and **3c** are shown in Figure 8, along with **3b** and **3d**, for comparison. The interactions have been analyzed with BIOVIA DSV2021 and with PacVIEW tool in PacDOCK web server.^59^ For brevity, herein only the most relevant features of the complexes are discussed; all specific stabilizing interactions and alternative views are discussed in Supporting Information. The calculated poses of **12a**, **12c**, and **15** (Figures S18–S21), and the detail of RO0505376 (Figure S14) into the binding site of the α_4_β_7_ headpiece (PDB 3 V4 V), are shown in Supporting Information. The calculated Δ*G*_bind_ nicely fit the experimental affinities for the isolated integrin: (kcal mol^–1^) **3a**, −15.81; **3b**, −14.08; **3c**, −15.08; **3d**, −14.72; **12a**, −15.28; **12c**, −12.84; **15**, −13.53.

All CPPs appear to occupy the same location into the crevice between the subunits, in proximity of the MIDAS center. With the only exception of **3d**, within the binding site the Phu^1^-LDV-isoAsp^5^ sequence of all CPPs can be read in a clockwise direction (Figure 8). Interestingly enough, for all CPPs but **3d** the coordination to Mg^2+^ in the MIDAS of the β_1_ subunit involves the carboxylate side chain of isoAsp^5^. For the prototypic **3a** and **3c**, this is in line with the experimental observation described above that isoAsp^5^ carboxylate rather than that of Asp^3^ was strictly necessary for receptor binding (**3a** vs **11a**, **12a**, **13**; **3c** vs **11c**, **12c**; Figure 3; Tables 1–3).

As anticipated, in the docked pose of the agonist **3a**, c[(*S*)-Phu^1^-LD^3^V-(*S*)-isoAsp^5^], isoAsp^5^COO^–^ is coordinated to Mg^2+^ in MIDAS, while Asp^3^COO^–^ interacts with Lys^157^NHζ^+^ (α_4_ subunit) by a salt bridge. The large majority of the stabilizing interactions of **3a** involve residues of the α subunit (Figure 8 and Figure S17). The aryl rings of Phu^1^ lean against the residues Tyr^187^ and Phe^214^ (α_4_), and the urea C=O is hydrogen-bonded to Lys^213^NH. The branched isopropylmethyl side chain of Leu^2^ finds a place in the upper hydrophobic pocket of the α/β-groove, delimited by Leu^225^, Tyr^187^, and Lys^157^, all belonging to the α_4_ subunit, a cavity which is not utilized by RO0505376 (Figure S14).^55^ Val^4^ adopts a pseudoaxial disposition, perpendicular to the macrocycle plane, making no relevant interactions.

The antagonist **3c**, c[(*R*)-Phu^1^-LD^3^V-(*S*)-isoAsp^5^], the diastereoisomer of **3a** for the reversal of configuration at Phu^1^, shows fewer interactions with the α subunit, compensated by tight interactions with residues of the β subunit (Figure 8 and Figure S17). As for **3a**, isoAsp^5^COO^–^ is coordinated to Mg^2+^ in MIDAS. Of particular interest is the ionic bond of Asp^3^COO^–^ with Lys^182^NHζ^+^ (β_1_), an interaction which pulls the CPP scaffold against the β_1_ subunit (see for comparison **12c**, c[(*R*)-Phu-LAV-(*S*)-isoAsp], Figure S20). This is in sharp contrast to **3a**, in which Asp^3^ interacts with Lys^157^ NHζ^+^ (α_4_). The pose of Phu^1^ is stabilized by interactions with Phe^321^ (π–π staking), Ser^227^, and Asp^227^ (β_1_). Interestingly, Val^4^ is in contact with Tyr^133^ (β_1_).

The CPP **3b**, c[(*S*)-Phu^1^-LDV-(*R*)-isoAsp], differs from **3a** for the inversion of the stereochemistry of isoAsp^5^, thus producing a rearrangement of the interactions around MIDAS. Clearly, **3b** shows more balanced interactions with both subunits (Figure 8). As for **3a**, isoAsp^5^COO^–^ is coordinated to Mg^2+^ (MIDAS), and Asp^3^COO^–^ forms a salt bridge with Lys^157^NHζ^+^ (α_4_). Val^4^ isopropyl makes some contacts with Ser^134^ (β_1_) and Tyr^133^ (β_1_). Phu^1^ interacts with residues of the α_4_ subunit, i.e. Tyr^187^ and Lys^213^, as well as residues of the β_1_ subunit, Ala^260^, Phe^321^, and Pro^228^.

As for **3b**, **3d** also seems to lean against residues of both subunits alike (Figure 8). Albeit the docked structure of **3d** occupies the same cleft, the pentapeptide ring appears turned over as compared to the other CPPs. The c[(*R*)-Phu^1^-LD^3^V-(*R*)-isoAsp^5^] sequence can be read in anticlockwise direction within the binding site, upon 180° rotation along an axis passing through Val^4^ and Phu^1^, so that these residues maintain the same positions. However, because of the rotation, Val^4^ adopts a pseudoequatorial position, in tight contact with Cys^187^ (β_1_). The rotation also produces the swap between isoAsp^5^ and Asp^3^; therefore, Mg^2+^ in MIDAS is coordinated to the carboxylate of Asp^3^, while isoAsp^5^ carboxylate forms a salt bridge with Lys^157^NHζ^+^ (α_4_). Plausibly, this alternative disposition of the macrolactam ring is dictated by the reversal of configuration at both Phu^1^ and isoAsp^5^ residues. As for Phu^1^, this residue is in contact with Tyr^187^, Phe^214^, and Lys^213^ of the α_4_ subunit, and with Pro^228^ and Ser^227^ of the β_1_ subunit.

Concerning the calculated poses of **12a**,**c** and **15**, these appear similar to those of the parent peptides **3a** and **3c** (Supporting Information, Figures S18–S21). Also for these derivatives, the trend of theoretical binding Δ*G*s is nicely consistent with the experimental binding affinities (see above).

The in-solution and bioactive conformations of **3a**–**d** are presented in Figure 6. The inspection of the structures supports the utility of the α/β hybrid CPP scaffolds as conformationally stable probes for investigating integrin binding in the absence of the crystal structure of the receptor.^60^ Indeed, the overall geometries are generally maintained at the receptor, with minor differences. For instance, the receptor-bound structure of **3a** shows the intramolecular hydrogen bond between Phu^1^C=O and Val^4^NH as observed in solution and a second hydrogen bond between Phu^1^C=O and Asp^3^NH (Figure 6). More pronounced differences can be perceived for **3d**.

For all CPPs, in the bioactive conformation the diphenylurea moiety resides in the lower side of the longitudinal cleft between the α and β subunits, consistent with the specificity of MPUPA for α_4_ integrins.^12^ Previous docking computations conducted for MPUPA-containing structures with molecular mechanics force fields gave alternative results,^36^ plausibly a consequence of the quantum mechanics approach.

Very recently, da Silva et al. docked BIO1211 into a homology model of the α_4_β_1_ integrin. These authors predicted the interaction of AspCOO^–^ with the divalent cation in MIDAS.^61^ In the calculated pose, the peptide adopts a reverse S-shape, spanning across the interface between the α_4_ and β_1_ subunits. The C-terminal Pro is positioned on top of the groove, while the N-terminal MPUPA is allocated within the lower side of the α/β groove, as observed for the CPPs. The LDVP sequence presents itself in anticlockwise direction. Val occupies the same position as seen for the CPPs, but its position is pseudoequatorial, so that the branched isopropyl points against the β_1_ subunit. As said, AspCOO^–^ is coordinated to the Mg^2+^ ion in the MIDAS. The side chain of Leu is directed toward the β_1_ subunit. Albeit this study is also the result of a homology modeling procedure, so that any correlation is purely indicative, this geometry of BIO1211 seems to have something in common with the docked pose of **3d**, rather than those of **3a**–**c** and the other CPPs.

With all due caution, the computations with our homology receptor model aroused some structural speculations. Despite a certain similarity, the predicted receptor-bound poses of the most potent agonist **3a** and the antagonist **3c** show some differences, possibly responsible for the alternative behavior of the two compounds in the integrin-mediated cell adhesion to the natural ligands.

In summary, the macrocycle of **3c** appears flattened into the binding site within the propeller and the βI-domain on the integrin head, making many contacts with the β_1_ subunit. The computations support the role of the ionic bond Asp^3^COO^–^-Lys^182^NHζ^+^ (β_1_), an interaction which pulls the CPP scaffold against the β_1_ subunit, in determining antagonism (Figure S20). Indeed, the substitution of Asp^3^ for Ala transformed the antagonist **3c** into the modest agonist **12c** (Tables 1–3). Furthermore, the simulations highlight the role of the aryl rings of Phu^1^ in the interactions of **3c** with residues adjacent to Asp^229^, a key residue of the β_1_ subunit which belongs to the coordination sphere of both MIDAS and SyMBS.

The mechanism of extension and activation requires a specific reorganization of pre-existing interaction networks around Tyr^133^ in the β1-α1 loop of the β subunit, in the proximity of the ligand recognition site.^62,63^ In this perspective, antagonism by **3c** might be the result of the combined compacting effects of Phu^1^, that clings to elements of MIDAS and SyMBS, and the bulky isopropyl group of **3c**, that packs against the Tyr^133^, therefore freezing domain translocation and hinge opening. As a consequence, the transmission of the activation signal through α_7_-helix downward movement and relative hybrid domain swing out in β_1_ cannot occur.^64^

On the other hand, the opposite absolute configuration at Phu^1^ forces **3a** to log into the binding site lopsided (Supporting Information, Figure S15), making fewer contacts with elements of the β_1_ subunit. In particular, Asp^3^COO^–^ makes a salt bridge with Lys^157^NHζ^+^ (α_4_), and Phu^1^ is in contact only with residues of the α_4_ subunit. The Tyr^133^ aryl ring nor other residues of the β1-α1 loop are tightly packed against the ligand, giving room for the dislocation of the β1-α1 loop of the βI domain necessary for receptor activation.

Also **12a**, which shares the same stereochemistry array of **3a**, maintains a lopsided orientation within the receptor, therefore having few contacts with the β_1_ subunit (Supporting Information, Figures S18 and S19), and indeed proved itself to be a good promoter of cell adhesion (Tables 1 and 3). The other CPPs (Supporting Information) adopt bioactive conformations which are intermediate between the flat **3c** and the lopsided **3a**, in general making interactions with both subunits, plausibly accounting for their inferior agonist effects (Tables 1 and 3).

Interestingly, in the α_4_β_1_–**3a** complex, the distance between the cations at MIDAS and ADMIDAS appears slightly increased by around 0.8 Å as compared to the α_4_β_1_–**3c** complex. This seems in contrast to the crystallographic evidence for β_3_ integrin. In the inactive conformation, the latter shows an acutely bent conformation. During agonist-induced headpiece opening, movements occur mainly in the β_3_ subunit, and the distance between β1-α1 loop elements and the α subunit decreases.^63^ The interaction of the ligand’s carboxylate with MIDAS seems to be necessary for receptor activation, while pulling by the α subunit may not be fundamental.^65^ During the conformational transition, the ADMIDAS experiences a noteworthy movement of 3.9 Å toward the MIDAS.^63^

On the other hand, a moderate increase of the distance between MIDAS and ADMIDAS, as calculated for the agonist **3a**, might make more sense for β_1_ integrins. Unlike the resting structures of β_3_ integrins, α_5_β_1_ integrin exhibited only a half-bent conformation.^66,67^ In β_1_ integrins, Ca^2+^ in the ADMIDAS seems to be a negative regulatory site responsible for integrin inhibition.^56^ It has been supposed that during receptor activation of β_1_ integrins, Ca^2+^ at the ADMIDAS site becomes highly mobile and eventually is expelled from the site, whereas that of LIMBS and MIDAS remains unchanged. Consistent with this, the inspection of the solid, close water-accessible surface of the α_4_β_1_–**3a** complex (Figure 8) shows that Ca^2+^ of the ADMIDAS is more exposed with respect to the α_4_β_1_–**3c** complex.

Finally, there is evidence that in α_5_β_1_ integrin the binding of small peptide ligands is not sufficient for full integrin opening.^56^ The extended, open conformation is observed only when both Mn^2+^ and FN are present,^68^ while Ca^2+^ binding to the ADMIDAS seems to stabilize the closed conformation.

In this scenario, our data for α_4_β_1_ integrins seem to suggest that ligand binding and the overall integrin conformation are less tightly coupled than for other integrins. The small agonist **3a** alone at the binding site seems capable of activating intracellular signaling as an agonist. However, this interaction is not sufficient to induce full receptor opening.^56^ Nevertheless, this agonist might act as a promoter of protein–protein-interaction (PPI),^69^ being capable to predispose the receptor to adopt a semiactivated conformation and to facilitate Ca^2+^ depletion. The large reorganization of integrin structure would be possible only as a result of subsequent FN binding.

## Conclusion

The CPPs described herein were proposed as potential ligands of α_4_ integrin. In particular, the CPP **3c**, c[(*R*)-Phu-LDV-(*S*)-isoAsp], was an antagonist of α_4_ integrins with moderate potency, while **3a**, c[(*S*)-Phu-LDV-(*S*)-isoAsp], appeared to be a potent agonist capable to increase both α_4_β_1_ and α_4_β_7_ integrin-mediated cell adhesion. In addition, **12a**, c[(*R*)-Phu-LAV-(*S*)-isoAsp], was an agonist which selectively promoted the adhesion of α_4_β_1_ with low nanomolar potency but not that of α_4_β_7_ integrin-expressing cells.

Recently, the agonists of α_4_β_1_ integrin garnered some interest for their potential in preventing the recruitment of circulating leukocytes by steadily blocking their rolling onto the endothelial surface, preventing them from reaching the sites of inflammation. Further developments might stem from potential applications of the agonist ligands in diagnostics or theranostics. These CPPs might serve as equivalents of the well-known integrin ligand c[RGDfK] which found a wide range of applications for targeting cancer cells, for cell growth, for regenerative medicine, etc.^23,70,71^

Finally, the constrained cyclic LDV peptides may represent suitable probes to explore the structural requirements with respect to the 3D arrangement of the pharmacophoric groups and the interactions with α_4_β_1_ integrin. To this purpose, we assembled a homology model of the receptor and we performed quantum mechanics computations to predicted ligand conformations within the receptor. It must be emphasized that the validation of the hybrid receptor model relies only on the docking of the ligands found in the parent crystallographic structure 3V4V, and the purely indicative comparison between the poses calculated with our receptor model and the binding pose of BIO1211 described in the literature, because also the latter is the result of homology modeling.

The binding geometries of **3a** and **3c** showed modest differences, despite significantly different functions. Plausibly, exhaustive MD studies might better differentiate the interactions on the basis of agonist and antagonist. Practical difficulties of performing long MD simulations clearly reverberate throughout the soundness of the discussion. Further studies are needed by pursuing MD simulations to ascertain if conformations sampled by **3a** and **3c** overlap to some extent, to confirm that interactions arising during the simulations are reasonably distinct and to verify if bioactive conformations are referable to that used in molecular modeling.

Albeit highly speculative, the simulations are suggestive of a possible role of the agonist **3a** as a small-molecule PPI stabilizer, capable of prearranging the receptor in a semiactivated conformation. While the inhibition of PPIs by means of small-molecule drugs that disrupt or prevent a binary protein complex represents a classic approach in pharmacology, the opposite strategy to stabilize PPIs with small molecules is still regarded as an “exotic” approach, scarcely explored in the integrin field.^72^

## Experimental Section

### General Procedures

Unless otherwise stated, standard chemicals and solvents were purchased from commercial sources and used as received without further purification. Target compounds were determined to be ≥95% pure by analytical HPLC analyses, performed on Agilent 1100 series apparatus, using a reverse-phase column Phenomenex mod. Gemini 3 μm C_18_ 110 Å 100 × 3.0 mm (no. 00D-4439-Y0); column description: stationary phase octadecyl-carbon-chain-bonded silica (C_18_) with trimethylsilyl end-cap, fully porous organosilica solid support, particle size 3 μm, pore size 110 Å, length 100 mm, internal diameter 3 mm; mobile phase for neutral compounds: from H_2_O/CH_3_CN (9:1) to H_2_O/CH_3_CN (2:8) in 20 min at a flow rate of 1.0 mL. min^–1^, followed by 10 min at the same composition; DAD (diode-array detection) 210 nm; mobile phase for ionizable peptides: from 9:1 H_2_O/CH_3_CN/0.1% HCOOH to 2:8 H_2_O/CH_3_CN/0.1% HCOOH in 20 min, flow rate of 1.0 mL min^–1^; DAD 254 nm. Semipreparative RP HPLC was carried out with an Agilent 1100 series apparatus, using reverse-phase column ZORBAX mod. Eclipse XDBC18 PrepHT cartridge 21.2 × 150 mm 7 μm (no. 977150-102); column description: stationary phase octadecyl-carbon-chain-bonded silica (C_18_), double end-capped, particle size 7 μm, pore size 80 Å, length 150 mm, internal diameter 21.2 mm; XSelect Peptide CSH C18 OBD column (Waters), 19 × 150 mm 5 μm (no. 186007021). column description: stationary phase octadecyl-carbon-chain-bonded silica (C_18_), double end-capped, particle size 5 μm, pore size 130 Å, length 150 mm, internal diameter 19 mm; DAD 210 nm, DAD 254 nm; gradient mobile phase from H_2_O/CH_3_CN (8:2) to CH_3_CN (100%) in 10 min at a flow rate of 12 mL. min^–1^, isocratic mobile phase 1:1 H_2_O/CH_3_CN/0.1% TFA in 8 min at a flow rate of 10 mL. min^–1^. Routine ESI MS analysis was carried out using an MS single quadrupole HP 1100 MSD detector, with a drying gas flow of 12.5 L min^–1^, nebulizer pressure 30 psig, drying gas temp 350 °C, capillary voltage 4500 (+) and 4000 (−), scan 50–2600 amu. High resolution mass spectrometry (HRMS) was performed with a Xevo G2XS QTof apparatus. NMR spectra were recorded on Varian Gemini apparatus (^1^H: 400 MHz, ^13^C: 100 MHz) or Bruker BioSpin GmbH (^1^H: 600 MHz, ^13^C: 150 MHz) at 298 K in 5 mm tubes, using 0.01 M peptide. Solvent suppression was carried out by the solvent presaturation procedure implemented in Varian (PRESAT). Chemical shifts are reported in ppm (δ) and referenced to the residual nondeuterated solvent signal as internal standard (CDCl_3_^1^H: 7.26 ppm, ^13^C: 77.16 ppm; (CD_3_)_2_SO: ^1^H: 2.50, ^13^C: 39.52 ppm). The unambiguous assignment of ^1^H NMR resonances was based on 2D gCOSY experiments. VT ^1^H NMR experiments were carried out over the range 298–348 K; temperature calibration was done with the ethylene glycol HO-CH_*n*_ chemical-shift separation method. Coupling constants (*J*) are reported in Hz. Solid-phase peptide synthesis was performed in polypropylene syringes fitted with a polyethylene porous disc. A dual-channel syringe pump (KD Scientific model 200) was used for slow reagent addition (cyclization in solution).

#### General Procedure for SPPS of Linear Peptides **9**

The linear peptides were assembled manually on Wang resin (0.3 g, 1.1 mmol/g loading capacity) using standard procedures. Prior to use, the resin was swollen in DMF (3 mL) for 15 min. In a separate vial, (*S*)- or (*R*)-Fmoc-Asp-OBn (0.3 mmol) and HOBT (0.3 mmol) were dissolved in DMF (4 mL). After 20 min, the mixture was added to the resin, followed by DCC (0.3 mmol) and a catalytic amount of DMAP, and the resin was gently shaken for 3 h at RT. Thereafter, a mixture of Ac_2_O (10 mmol) and pyridine (10 mmol) was added and shaken for additional 30 min to end-cap the unreacted 4-hydroxybenzyl alcohol linkers. The resin was filtered and washed alternatively with DMF, MeOH, and DCM (3 × 4 mL each).

Fmoc cleavage was carried out using 20% (*v/v*) piperidine in DMF (5 mL), while gently shaking at RT for 10 min. After washing with DMF and DCM (5 mL), the deprotection was repeated. The resin was then washed sequentially with DMF, MeOH, and DCM (3 × 4 mL each).

The subsequent coupling reactions were performed by dissolving in a separate vial Fmoc-protected amino acids (0.3 mmol) and HOBt (0.3 mmol) in DMF (4 mL) for 20 min. The last introduced residue was Boc-Phu-OH. The mixture was poured into the reactor followed by DCC (0.3 mmol), and the suspension was shaken for 3 h at RT. Coupling efficacy was monitored by the Kaiser test.

Cleavage from the resin and simultaneous removal of the Boc protecting group was performed by using a 95:2.5:2.5 *v/v/v* mixture of TFA/TIPS/H_2_O (10 mL) while shaking for 2.5 h at RT. The mixture was filtered and the resin washed twice with Et_2_O/DCM containing a small portion of TFA. The filtrates were collected and solvents were removed under reduced pressure, and ice-cold Et_2_O was added to precipitate the crude peptides as TFA salts, which were recovered by centrifuge and used for the cyclization without further purification (Supporting Information). Peptide identity was confirmed by ESI MS (Supporting Information).

#### General Procedure for Synthesis of the CPPs

The cyclization of the crude peptide was performed under pseudo-high dilution conditions. A solution of the linear peptides (0.15 mmol) in DMF (10 mL) was added over 16 h using a syringe pump, to a mixture of HBTU (0.45 mmol), HOBt (0.45 mmol), and DIPEA (0.9 mmol) in DMF at RT. Once the addition was complete, the reaction was stirred for additional 2 h. Then the solvent was distilled at reduced pressure, and the crude peptides were isolated by RP HPLC on a semipreparative C18 column (General Methods). Compound identity was confirmed by ESI MS (Supporting Information), in reasonable yield (Supporting Information).

Removal of benzyl protecting groups was performed by catalytic hydrogenation. A stirred suspension of the protected cyclopentapeptide **10** (0.1 mmol) and a catalytic amount of 10% *w/w* Pd/C in absolute EtOH (10 mL) was stirred under H_2_ atmosphere for 12 h at RT. Thereafter, the catalyst was filtered off over Celite and the solvent was distilled under reduced pressure, to afford the final products **3a**–**d** in quantitative yield. The purity (Table 1) and the identity of the products were determined to be >95% by RP HPLC coupled to ESI MS, by ^1^H and ^13^C NMR, and by 2D gCOSY experiments at 400 MHz in 8:2 DMSO-*d*_6_/H_2_O.

##### c[(*S*)-Phu-Leu-Asp-Val-(*S*)-isoAsp] **3a**

^1^H NMR (400 MHz, 8:2 DMSO-*d*_6_/H_2_O) δ 8.98 (s, 1H, PhuNHb), 8.65 (d, *J* = 6.0 Hz, 1H, LeuNH), 8.22–8.14 (m, 2H, AspNH + PhuNH), 7.90 (s, 1H, PhuNHa), 7.80 (d, *J* = 7.6 Hz, 1H, ArH_6_), 7.75 (d, *J* = 9.2 Hz, 1H, ValNH), 7.37 (d, *J* = 7.6 Hz, 2H, ArH_2′6′_), 7.22–7.09 (m, 5H, ArH_3′5′_+ArH_3,5_+isoAspNH), 6.94 (dd, *J* = 7.2, 6.8 Hz, 1H, ArH_4_), 4.60–4.53 (m, 1H, isoAspHα), 4.38–4.31 (m, 1H, PhuHα), 4.29–4.24 (m, 1H, AspHα), 4.16 (dd, *J* = 9.2, 4.0 Hz, 1H, ValHα), 3.79–3.68 (m, 1H, LeuHα), 2.97–2.89 (m, 2H, PhuCHβ+AspHβ), 2.87 (dd, *J* = 14.0, 7.6 Hz, 1H, AspHβ), 2.74 (dd, *J* = 14.0, 2.0 Hz, 1H, PhuCHβ), 2.69 (dd, *J* = 14.4, 2.4 Hz, 1H, isoAspHβ), 2.61 (dd, *J* = 14.4, 4.0 Hz, 1H, isoAspHβ), 2.34–2.25 (m, 1H, ValHβ), 2.23 (s, 3H, ArCH_3_), 1.73–1.62 (m, 1H, LeuHβ), 1.55–1.43 (m, 1H, LeuHβ), 1.40–1.30 (m, 1H, LeuHγ), 0.91–0.76 (m, 12H, ValCH_3_+LeuCH_3_); ^13^C NMR (100 MHz, DMSO-*d*_6_) δ 172.5, 172.1, 171.9, 171.2, 170.5, 170.4, 169.8, 152.7, 138.3, 137.5, 130.7, 130.2, 129.3, 127.5, 126.1, 122.6, 121.0, 117.8, 57.7, 55.5, 52.5, 51.8, 48.4, 37.9, 36.9, 36.0, 35.2, 29.3, 24.2, 23.4, 21.2, 19.8, 17.9, 17.6. HRMS-ESI/QTOF *m*/*z* calcd for [C_36_H_48_N_7_O_10_]^+^ 738.34627, found 738.34654 [M + H]^+^.

##### c[(*S*)-Phu-Leu-Asp-Val-(*R*)-isoAsp] **3b**

^1^H NMR (400 MHz, 8:2 DMSO-*d*_6_/H_2_O) δ 9.02 (s, 1H, PhuNHb), 8.49 (br d, 1H, AspNH), 8.38 (d, *J* = 8.4 Hz, 1H, LeuNH), 8.32 (d, *J* = 8.4 Hz, 1H, PhuNH), 8.20 (br d, 1H, ValNH), 8.01 (br d, 1H, isoAspNH), 7.94 (s, 1H, PhuNHa), 7.80 (d, *J* = 8.0 Hz, 1H, ArH_6_), 7.36 (d, *J* = 8.0 Hz, 2H, ArH_2′6′_), 7.18–7.11 (m, 4H, ArH_3′5′_+ArH_3,5_), 6.93 (dd, *J* = 7.6, 7.2 Hz, 1H, ArH_4_), 4.40–4.32 (m, 2H, AspHα+PhuHα), 4.27–4.17 (m, 2H, isoAspHα+LeuHα), 3.96 (dd, *J* = 9.2, 8.8 Hz, 1H, ValHα), 3.03 (dd, *J* = 14.2, 3.8 Hz, 1H, PhuHβ), 2.80–2.68 (m, 4H, PhuHβ+isoAspHβ+AspHβ), 2.38 (dd, *J* = 15.2, 1.6 Hz, 1H, isoAspHβ), 2.23 (s, 3H, ArCH_3_), 2.13–2.05 (m, 1H, ValHβ), 1.72–1.61 (m, 1H, LeuHβ), 1.50–1.48 (m, 2H, LeuHβ+LeuHγ), 0.87–0.85 (m, 12H, ValCH_3_+LeuCH_3_); ^13^C NMR (100 MHz, DMSO-*d*_6_) δ 171.3, 171.0, 170.5, 169.7, 169.2, 152.7, 138.3, 137.5, 130.2, 129.3, 127.5, 126.1, 122.6, 121.1, 118.5, 117.9, 58.1, 52.6, 51.6, 49.1, 48.0, 34.5, 34.3, 31.3, 28.7, 24.2, 22.9, 22.1, 21.6, 19.5, 17.9. HRMS-ESI/QTOF *m*/*z* calcd for [C_36_H_48_N_7_O_10_]^+^ 738.34627, found 738.34599 [M + H]^+^.

##### c[(*R*)-Phu-Leu-Asp-Val-(*S*)-isoAsp] **3c**

^1^H NMR (400 MHz, 8:2 DMSO-*d*_6_/H_2_O) δ 9.04 (s, 1H, PhuNHb), 8.30 (br d, 1H, PhuNH), 8.25–8.15 (m, 2H, isoAspNH+LeuNH), 8.02 (br d, 1H, ValNH), 7.93 (s, 1H, PhuNHa), 7.79 (d, *J* = 8.4 Hz, 1H, ArH_6_), 7.70 (d, *J* = 6.4 Hz, 1H, Asp-NH), 7.34 (d, *J* = 8.0 Hz, 2H, ArH_2′6′_), 7.17–7.11 (m, 2H, ArH_3,5_), 7.06 (d, *J* = 8.0 Hz, 2H, ArH_3′5′_), 6.93 (t, *J* = 7.2 Hz, 1H, ArH_4_), 4.48–4.42 (m, 1H, AspHα), 4.38–4.35 (m, 1H, PhuHα), 4.32–4.25 (m, 1H, isoAspHα), 4.02–3.92 (m, 1H, LeuHα), 3.58–3.49 (m, 1H, ValHα), 2.79–2.73 (m, 3H, PhuHβ+AspHβ), 2.68–2.55 (m, 3H, isoAspHβ+AspHβ), 2.36–2.26 (m, 1H, ValHβ), 2.22 (s, 3H, ArCH_3_), 1.38–1.28 (m, 2H, LeuHβ), 1.02–0.92 (m, 1H, LeuCHγ), 0.83 (d, *J* = 6.4 Hz, 3H, LeuCH_3_), 0.79 (d, *J* = 6.4 Hz, 3H, LeuCH_3_), 0.72 (d, *J* = 6.0 Hz, 3H, ValCH_3_), 0.64 (d, *J* = 6.4 Hz, 3H, ValCH_3_); ^13^C NMR (100 MHz, DMSO-*d*_6_) δ 172.3, 172.0, 171.8, 171.1, 170.1, 169.8, 152.7, 138.4, 137.5, 130.1, 130.0, 129.4, 127.5, 126.1, 122.5, 121.0, 117.6, 109.5, 61.3, 54.9, 51.5, 51.2, 49.6, 36.4, 35.9, 33.6, 31.3, 23.5, 23.0, 22.1, 21.0, 19.1, 17.9. HRMS-ESI/QTOF *m*/*z* calcd for [C_36_H_48_N_7_O_10_]^+^ 738.34627, found 738.34688 [M + H]^+^.

##### c[(*R*)-Phu-Leu-Asp-Val-(*R*)-isoAsp] **3d**

^1^H NMR (400 MHz, 8:2 DMSO-*d*_6_/H_2_O) δ 8.99 (s, 1H, PhuNHb), 8.55 (d, *J* = 6.4 Hz, 1H, ValNH), 8.51 (d, *J* = 4.8 Hz, 1H, PhuNH), 8.42 (d, *J* = 6.8 Hz, 1H, isoAspNH), 8.24 (d, *J* = 8.4 Hz, 1H, LeuNH), 7.90 (s, 1H, PhuNHa), 7.80 (d, *J* = 7.6 Hz, 1H, ArH6), 7.35 (d, *J* = 7.6 Hz, 3H, AspNH+ArH2′6′), 7.19–7.10 (m, 2H, ArH3,5), 7.09 (d, *J* = 8.0 Hz, 2H, ArH3′5′), 6.94 (dd, *J* = 7.6, 7.2, Hz, 1H, ArH4), 4.54 (dd, *J* = 12.4, 4.4 Hz, 1H, AspHα), 4.42–4.35 (m, 1H, isoAspHα), 4.29–4.21 (m, 1H, PhuHα), 3.97–3.89 (m, 1H, LeuHα), 3.24–3.17 (m, 1H, ValHα), 2.84–2.69 (m, 3H, PhuHβ+AspHβ), 2.682.54 (m, 3H, isoAspHβ+ValHβ), 2.46–2.42 (m, 1H, AspHβ), 2.23 (s, 3H, ArCH_3_), 1.40–1.31 (m, 1H, LeuHβ), 1.30–1.22 (m, 1H, LeuHβ), 0.84 (d, *J* = 6.4 Hz, 7H, ValCH_3_+LeuHγ) 0.68 (d, *J* = 6.4 Hz, 3H, LeuCH_3_), 0.58 (d, *J* = 5.6 Hz, 3H, LeuCH_3_); ^13^C NMR (100 MHz, DMSO-*d*_6_) δ 172.0, 171.9, 171.7, 171.3, 170.5, 170.3, 170.1, 152.6, 138.4, 137.4, 130.1, 129.7, 129.4, 127.5, 126.1, 122.6, 121.1, 117.6, 64.5, 55.4, 51.2, 50.3, 48.8, 36.1, 35.8, 34.9, 27.6, 23.3, 23.1, 20.8, 19.5, 19.2, 17.8. HRMS-ESI/QTOF *m*/*z* calcd for [C_36_H_48_N_7_O_10_]^+^ 738.34627, found 738.34701 [M + H]^+^.

##### c[(*S*)-Phu-Leu-Asp-Val-(*R*)-β^3^homoAla] **11a**

^1^H NMR (400 MHz, 8:2 DMSO-*d*_6_/H_2_O) δ 9.01 (s, 1H, PhuNHb), 8.71 (d, *J* = 6.8 Hz, 1H, LeuNH), 8.07 (d, *J* = 7.2 Hz, 1H, AspNH), 7.92 (s, 1H, PhuNHa), 7.81 (d, *J* = 8.4 Hz, 2H, PhuNH+ArH6), 7.59 (d, *J* = 9.6 Hz, 1H, ValNH), 7.37 (d, *J* = 8.8 Hz, 2H, ArH2′6′), 7.17 (d, *J* = 8.8 Hz, 3H, ArH3′5′+ArH3), 7.12 (d, *J* = 8.4 Hz, 1H, ArH5), 7.03 (d, *J* = 7.6 Hz, 1H, β^3^AlaNH), 6.93 (dd, *J* = 8.0, 7.2 Hz, 1H, ArH4), 4.41 (dd, *J* = 15.2, 7.2 Hz, 1H, PhuHα), 4.24 (dd, *J* = 12.8, 7.2 Hz, 1H, AspHα), 4.16–4.14 (m, 1H, β^3^AlaHβ), 4.11 (dd, *J* = 9.6, 5.6 Hz, 1H, ValHα), 3.66–3.60 (m, 1H, LeuHα), 2.91–2.84 (m, 3H, PuHβ+AspHβ), 2.76 (dd, *J* = 12.8, 8.0 Hz, 1H, PhuHβ), 2.50 (m, 1H, β^3^AlaHα), 2.23–2.20 (m, 4H, ArCH_3_+ValHβ), 2.01 (dd, *J* = 13.2, 6.4 Hz, 1H, β^3^AlaHα), 1.76–1.69 (m, 1H, LeuHβ), 1.501.43 (m, 1H, LeuHβ), 1.23–1.18 (m, 1H, LeuHγ), 1.11 (d, *J* = 6.4 Hz, 3H, β^3^AlaCH_3_), 0.86 (d, *J* = 7.2 Hz, 6H, ValCH_3_), 0.82 (d, *J* = 6.4 Hz, 3H, LeuCH_3_), 0.78 (d, *J* = 6.0 Hz, 3H, LeuCH_3_); ^13^C NMR (101 MHz, DMSO-*d*_6_) δ 172.5, 172.2, 171.5, 170.5, 170.4, 169.7, 152.7, 138.4, 137.5, 130.4, 130.2, 129.3, 127.5, 126.1, 122.6, 121.0, 117.8, 57.9, 54.6, 52.7, 52.3, 42.8, 41.3, 37.2, 36.3, 34.8, 24.1, 23.6, 21.1, 20.4, 19.8, 18.0, 17.8. HRMS-ESI/QTOF *m*/*z* calcd for [C_36_H_50_N_7_O_8_]^+^ 708.37209, found 708.37190 [M + H]^+^.

##### c[(*S*)-Phu-Leu-Ala-Val-(*S*)-isoAsp] **12a**

^1^H NMR (400 MHz, 8:2 DMSO-*d*_6_/H_2_O) δ 10.56 (br d, 1H, LeuNH), 9.18 (s, 1H, PhuNHb), 9.06 (br d, 1H, PhuNH), 8.58 (br d, 1H, AlaNH), 8.05 (s, 1H, PhuNHa), 7.80 (d, *J* = 7.6 Hz, 1H, ArH6), 7.51 (d, *J* = 9.6 Hz, 1H, ValNH), 7.40 (d, *J* = 8.4 Hz, 2H, ArH2′6′), 7.18–7.09 (m, 2H, ArH3,5), 7.05 (d, *J* = 8.0 Hz, 2H, ArH3′5′), 6.93 (dd, *J* = 7.6, 7.2, Hz, 1H, ArH4), 6.86 (d, *J* = 6.0 Hz, 1H, isoAspNH), 4.40 (d, *J* = 8.8 Hz, 1H, ValHα), 4.26 (dd, *J* = 7.2, 6.4 Hz, 1H, AlaHα), 4.12–4.05 (m, 1H, isoAspHα), 4.04–3.92 (m, 2H, PhuHα+LeuHα), 2.94–2.80 (m, 2H, PhuHβ+isoAspHβ), 2.69–2.56 (m, 2H, PhuCHβ+isoAspHβ), 2.56–2.49 (m, 1H, ValHβ), 2.24 (s, 3H, ArCH_3_), 1.87–1.76 (m, 1H, LeuHβ), 1.40 (d, *J* = 7.6 Hz, 3H, AlaCH_3_), 1.15–1.01 (m, 4H, LeuHβ+ValCH_3_), 0.92–0.79 (m, 4H, ValCH_3_+LeuCHγ), 0.73 (s, 3H, LeuCH_3_), 0.57 (s, 3H, LeuCH_3_); ^13^C NMR (100 MHz, DMSO-*d*_6_) δ 176.5, 175.9, 172.2, 171.7, 171.6, 168.6, 152.7, 138.7, 137.5, 130.1, 129.3, 127.6, 126.1, 122.6, 121.1, 117.7, 56.4, 55.9, 51.8, 51.0, 50.3, 36.2, 35.8, 30.8, 28.1, 23.9, 20.0, 19.5, 18.0, 17.1, 16.6. HRMS-ESI/QTOF *m*/*z* calcd for [C_35_H_48_N_7_O_8_]^+^ 694.35644, found 694.35596 [M + H]^+^.

##### c[(*S*)-PhU-Leu-Asp-Val-(*S*)-isoAsp(nPr)] **13**

^1^H NMR (400 MHz, 8:2 DMSO-*d*_6_/H_2_O) δ 9.08 (br s, 1H, PhuNHb), 8.73 (d, *J* = 7.2 Hz, 1H, LeuNH), 8.25 (d, *J* = 5.2 Hz, 1H, AspNH), 7.99 (br s, 1H, PhuNHa), 7.96 (br d, 1H, PhuNH), 7.82 (d, *J* = 8.0 Hz, 1H, ArH6), 7.71 (d, *J* = 8.8 Hz, 1H, ValNH), 7.46–7.41 (m, 1H, propyl-NH), 7.38 (d, *J* = 8.4 Hz, 2H, ArH2′6′), 7.22–7.10 (m, 5H, ArH3′5′+ArH3,5+isoAspNH), 6.93 (t, *J* = 7.2 Hz, 1H, ArH4), 4.44 (dd, *J* = 12.4, 7.6 Hz, 1H, isoAspHα), 4.32–4.25 (m, 2H, PhuHα+AspHα), 4.15 (dd, *J* = 8.8, 6.0 Hz, 1H, ValHα), 3.72–3.64 (m, 1H, LeuHα), 3.01–2.94 (m, 2H, propylCH_2_), 2.91–2.80 (m, 4H, PhuCHβ+AspHβ), 2.65 (dd, *J* = 14.0, 4.0 Hz, 1H, isoAspHα), 2.55–2.50 (m, 1H, isoAspHα), 2.34–2.25 (m, 1H, ValHβ), 2.24 (s, 3H, ArCH_3_), 1.72–1.63 (m, 1H, LeuHβ), 1.52–1.43 (m, 1H, LeuHβ), 1.42–1.32 (m, 3H, propylCH_2_+LeuHγ), 0.90–0.74 (m, 15H, ValCH_3_+LeuCH_3_ + propylCH_3_); ^13^C NMR (100 MHz, DMSO-*d*_6_) δ 172.5, 172.2, 171.4, 170.7, 170.4, 170.2, 170.0, 152.7, 138.5, 137.5, 130.3, 130.2, 129.3, 127.5, 126.1, 122.6, 121.0, 117.8, 58.0, 55.5, 52.5, 51.9, 50.5, 40.4, 37.5, 37.2, 36.0, 34.9, 31.3, 28.7, 24.1, 23.5, 22.2, 21.0, 20.0, 18.0, 17.9, 11.2. HRMS-ESI/QTOF *m*/*z* calcd for [C_39_H_55_N_8_O_9_]^+^ 779.40920, found 779.40883 [M + H]^+^.

##### c[(*R*)-Phu-Leu-Asp-Val-(*R*)-β^3^homoAla] **11c**

^1^H NMR (400 MHz, 8:2 DMSO-*d*_6_/H_2_O) δ 9.05 (s, 1H, PhuNHb), 8.39 (d, *J* = 4.8 Hz, 1H, PhuNH), 8.15 (d, *J* = 8.4 Hz, 1H, LeuNH), 7.93 (s, 1H, PhuNHa), 7.88 (d, *J* = 7.2 Hz, 1H, β^3^AlaNH), 7.85 (d, *J* = 8.4 Hz, 1H, ValNH), 7.82 (d, *J* = 8.0 Hz, 1H, AspNH), 7.79 (d, *J* = 8.0 Hz, 1H, ArH6), 7.36 (d, *J* = 8.4 Hz, 2H, ArH2′6′), 7.15 (t, *J* = 8.2 Hz, 1H, ArH5), 7.09 (d, *J* = 8.4 Hz, 3H, ArH3′5′+ArH3), 6.93 (t, *J* = 7.2 Hz, 1H, ArH4), 4.60 (dd, *J* = 15.2, 8.0 Hz, 1H, AspHα), 4.28 (dd, *J* = 13.6, 6.4 Hz, 1H, PhuHα), 4.01 (dd, *J* = 11.6, 8.0 Hz, 1H, LeuHα), 3.95–3.92 (m, 1H, β^3^AlaHβ), 3.63 (t, *J* = 7.2 Hz, 1H, ValHα), 2.91 (dd, *J* = 14.0, 8.4 Hz, 1H, AspHβ), 2.87 (dd, *J* = 12.0, 5.6 Hz, 1H, PhuCHβ), 2.76 (dd, *J* = 14.4, 8.8 Hz, 1H, PhuHβ), 2.55–2.50 (m, 1H, AspHβ), 2.39 (dd, *J* = 13.6 5.6 Hz, 1H, β^3^AlaHα), 2.28 (dd, *J* = , 14.0, 6.8 Hz, 1H, ValHβ), 2.23 (s, 3H, ArCH_3_), 2.16 (dd, *J* = 13.2, 3.6 Hz, 1H, β^3^AlaHα), 1.37 (dd, *J* = 19.2, 6.8 Hz, 2H, LeuHβ), 1.26–1.10 (m, 1H, LeuHγ), 1.05 (d, *J* = 6.8 Hz, 3H, β^3^AlaCH_3_), 0.84 (d, *J* = 6.8 Hz, 3H, ValCH_3_), 0.80 (d, *J* = 6.4 Hz, 3H, ValCH_3_), 0.70 (d, *J* = 6.0 Hz, 3H, LeuCH_3_), 0.62 (d, *J* = 5.6 Hz, 3H, Leu-CH_3_); ^13^C NMR (100 MHz, DMSO-*d*_6_) δ 171.8, 171.7, 171.3, 171.1, 169.5, 169.2, 152.7, 138.5, 137.5, 130.1, 129.7, 129.5, 127.5, 126.1, 122.6, 121.0, 117.6, 60.4, 55.7, 51.0, 50.7, 42.7, 41.9, 36.3, 36.0, 29.0, 28.5, 23.3, 23.1, 21.0, 19.6, 19.5, 18.7, 17.9. HRMS-ESI/QTOF *m*/*z* calcd for [C_36_H_50_N_7_O_8_]^+^ 708.37209, found 708.37287 [M + H]^+^.

##### c[(*R*)-Phu-Leu-Ala-Val-(*S*)-isoAsp] **12c**

^1^H NMR (400 MHz, 8:2 DMSO-*d*_6_/H_2_O) δ 9.09 (s, 1H, PhuNHb), 8.36 (br s, 1H, PhuNH), 8.12 (d, *J* = 6.8 Hz, 1H, LeuNH), 8.00 (s, 1H, PhuNHa), 7.93–7.88 (m, 2H, ValNH+isoAspNH), 7.80 (d, *J* = 8.0 Hz, 1H, ArH_6_), 7.76 (br d, 1H, AlaNH), 7.35 (d, *J* = 7.6 Hz, 2H, ArH_2′6′_), 7.18–7.09 (m, 2H, ArH_3,5_), 7.06 (d, *J* = 7.6 Hz, 2H, ArH_3′5′_), 6.93 (dd, *J* = 7.2, 6.8 Hz, 1H, ArH_4_), 4.41–4.30 (m, 2H, PhuHα+isoAspHα), 4.16 (dd, *J* = 7.2, 6.8 Hz, 1H, AlaHα), 4.00–3.92 (m, 1H, LeuHα), 3.71 (dd, *J* = 8.0, 6.4 Hz, 1H, ValHα), 2.78 (d, *J* = 7.2 Hz, 2H, PhuHβ), 2.62–2.52 (m, 2H, isoAspHβ), 2.33–2.24 (m, 1H, ValHβ), 2.23 (s, 3H, ArCH_3_), 1.39–1.31 (m, 2H, LeuHβ), 1.25 (d, *J* = 6.8 Hz, 3H, AlaCH_3_), 1.12–1.03 (m, 1H, LeuHγ), 0.85 (d, *J* = 6.4 Hz, 3H, ValCH_3_), 0.80 (d, *J* = 6.4 Hz, 3H, ValCH_3_), 0.76 (d, *J* = 6.4 Hz, 3H, LeuCH_3_), 0.68 (d, *J* = 6.0 Hz, 3H, LeuCH_3_); ^13^C NMR (100 MHz, DMSO-*d*_6_) δ 172.6, 171.7, 171.5, 171.1, 169.9, 169.8, 152.7, 138.4, 137.5, 130.2, 130.1, 129.5, 127.6, 126.1, 122.6, 121.1, 117.6, 66.4, 59.9, 54.9, 52.0, 50.1, 36.7, 28.9, 23.7, 23.1, 21.1, 19.2, 18.6, 17.9, 17.7. HRMS-ESI/QTOF *m*/*z* calcd for [C_35_H_48_N_7_O_8_]^+^ 694.35644, found 694.35665 [M + H]^+^.

##### c[(*S*)-Phu-Phe-Asp-Val-(*S*)-isoAsp] **14**

^1^H NMR (600 MHz, 8:2 DMSO-d_6_*/*H_2_O) δ 8.93 (s, 1H, PhuNHb), 8.72 (d, *J* = 7.2 Hz, 1H, PheNH), 8.21–8.16 (m, 2H, AspNH+PhuNH), 7.89 (s, 1H, PhuNHa), 7.82 (d, *J* = 7.8 Hz, 1H, ArH_6_), 7.70 (d, *J* = 9.5 Hz, 1H, ValNH), 7.32 (d, *J* = 8.5 Hz, 2H, ArH_2′6′_), 7.29 (t, *J* = 7.6 Hz, 2H, PheArH), 7.21–7.15 (m, 4H, PheArH+ArH_3_), 7.13 (t, *J* = 7.7 Hz, 1H, ArH_5_), 7.08 (d, *J* = 8.4 Hz, 2H, ArH_3′5′_), 7.06 (d, *J* = 8.2 Hz, 1H, isoAspNH), 6.93 (dd, *J* = 7.8, 7.2 Hz, 1H, ArH_4_), 4.57 (dt, *J* = 8.2, 5.5 Hz, 1H, isoAspHα), 4.31 (td, *J* = 7.6, 5.4 Hz, 1H, PhuHα), 4.26–4.19 (m, 2H, AspHα+ValHα), 4.02 (ddd, *J* = 11.3, 7.1, 4.6 Hz, 1H, PheHα), 3.22 (dd, *J* = 13.8, 4.6 Hz, 1H, PheHβ), 3.06–2.96 (m, 2H, PheHβ+PhuHβ), 2.86 (dd, *J* = 16.6, 7.8 Hz, 1H, PhuHβ), 2.69 (dd, *J* = 14.6, 5.1 Hz, 1H, isoAspHβ), 2.59–2.52 (m, 3H, isoAspHβ+AspHβ), 2.37–2.32 (m, 1H, ValHβ), 2.23 (s, 3H, ArCH3), 0.95–0.87 (m, 6H, ValCH_3_); ^13^C NMR (150 MHz, DMSO-*d*_6_) δ 172.6, 172.1, 170.6, 170.5, 170.3, 169.7, 152.6, 138.5, 138.2, 137.4, 133.5, 131.1, 130.2, 129.6, 129.3, 129.1, 128.2, 127.4, 126.3, 126.1, 122.6, 121.0, 117.9, 57.6, 55.7, 55.5, 51.8, 48.4, 40.1, 36.9, 35.8, 35.1, 34.7, 30.7, 29.2, 19.8, 17.9, 17.5. HRMS-ESI/QTOF *m*/*z* calcd for [C_39_H_46_N_7_O_10_]^+^ 772.33062, found 772.33004 [M + H]^+^.

##### c[(*S*)-Phu-Phe-Ala-Val-(*S*)-isoAsp] **15**

^1^H NMR (400 MHz, 8:2 DMSO-*d*_6_*/*H_2_O) δ 8.94 (s, 1H, PhuNHb), 8.51 (d, *J* = 7.2 Hz, 1H, PheNH), 8.21–8.09 (m, 2H, AlaNH+PhuNH), 7.90 (s, 1H, PhuNHa), 7.86–7.75 (m, 2H, ArH_6_+ValNH), 7.35–7.25 (m, 4H, ArH_2′6′_+PheArH), 7.23–7.17 (m, 3H, PheArH), 7.16 (d, *J* = 8.0 Hz, 1H, ArH_3_), 7.14–7.08 (m, 2H, ArH_5_+isoAspNH), 7.04 (d, *J* = 8.2 Hz, 2H, ArH_3′5′_), 6.93 (t, *J* = 7.4 Hz, 1H, ArH_4_), 4.60 (dt, *J* = 9.3, 5.0 Hz, 1H, isoAspHα), 4.22–4.12 (m, 2H, ValHα+PhuHα), 4.12–4.04 (m, 1H, PheHα), 3.98 (t, *J* = 7.1 Hz, 1H, AlaHα), 3.22–3.15 (m, 1H, PheHβ), 3.07–2.98 (m, 1H, PheHβ), 2.64 (t, *J* = 5.7 Hz, 2H, isoAspHβ), 2.57 (d, *J* = 7.5 Hz, 2H, PhuHβ), 2.38–2.30 (m, 1H, ValHβ), 2.23 (s, 3H, ArCH3), 1.40 (d, *J* = 7.1 Hz, 3H, AlaCH_3_), 0.90 (dd, *J* = 6.9 Hz, 6H, ValCH_3_); ^13^C NMR (100 MHz, DMSO-*d*_6_) δ 172.1, 172.02, 172.00, 170.6, 170.5, 169.9, 152.7, 138.4, 138.2, 137.4, 131.1, 130.2, 129.3, 129.1, 128.2, 127.4, 126.3, 126.1, 122.6, 121.0, 117.9, 57.5, 55.8, 55.7, 50.7, 48.1, 36.8, 36.0, 35.2, 29.2, 19.8, 17.9, 17.5, 16.7. HRMS-ESI/QTOF *m*/*z* calcd for [C_38_H_46_N_7_O_8_]^+^ 728.34079, found 728.34111 [M + H]^+^.

##### c[(*R*)-Phu-Leu-Asp-Phg-(*S*)-isoAsp] **16**

^1^H NMR (400 MHz, 8:2 DMSO-*d*_6_*/*H_2_O) δ 8.95 (s, 1H, PhuNHb), 8.57 (d, *J* = 8.3 Hz, 1H, PhuNH), 8.46 (d, *J* = 6.3 Hz, 1H, PhgNH), 8.14–8.09 (m, 2H, AspNH+LeuNH), 8.04 (d, *J* = 8.7 Hz, 1H, AspNH), 7.88 (s, 1H, PhuNHa), 7.81 (d, *J* = 8.2 Hz, 1H, ArH_6_), 7.35 (d, *J* = 8.1 Hz, 2H, ArH_2′6′_), 7.33–7.18 (m, 5H, PhgArH), 7.17–7.10 (m, 2H, ArH_3_+ArH_5_), 7.05 (d, *J* = 8.2 Hz, 2H, ArH_3′5′_), 6.94 (td, *J* = 7.4, 1.3 Hz, 1H, ArH_4_), 5.15 (d, *J* = 6.3 Hz, 1H, PhgHα), 4.56–4.47 (m, 2H, PhuHα+isoAspHα), 4.46–4.41 (m, 1H, AspHα), 3.97 (ddd, *J* = 10.4, 7.2, 4.9 Hz, 1H, LeuHα), 2.82 (d, *J* = 7.4 Hz, 2H, isoAspHβ), 2.76–2.69 (m, 2H, AspHβ), 2.64–2.52 (m, 2H, PhuHβ), 2.23 (s, 3H, ArCH3), 1.38–1.23 (m, 2H, LeuHβ), 1.21–1.13 (m, 1H, LeuHγ), 0.75 (dd, *J* = 29.4, 6.5 Hz, 6H, LeuCH3); ^13^C NMR (100 MHz, DMSO-*d*_6_) δ 172.3, 172.1, 171.6, 170.6, 169.7, 169.0, 168.7, 152.6, 138.33, 138.28, 137.5, 130.21, 130.18, 129.6, 129.5, 127.9, 127.5, 126.8, 126.1, 122.6, 121.0, 117.6, 57.7, 54.4, 52.3, 51.5, 49.7, 40.4, 37.3, 37.15, 37.14, 36.1, 23.7, 23.0, 21.3, 17.9. HRMS-ESI/QTOF *m*/*z* calcd for [C_39_H_46_N_7_O_10_]^+^ 772.33062, found 772.33102 [M + H]^+^.

#### Cell Adhesion Assays

For adhesion assays on Jurkat E6.1, RPMI8866, or HL60 cells, black 96-well plates were coated overnight at 4 °C with VCAM-1 or ICAM-1 or MAdCAM-1 (5 μg/mL) or Fg (10 μg/mL). The cells were counted, stained with CellTracker green CMFDA (12.5 μM, 30 min at 37 °C, Life Technologies), and after three washes, were preincubated with increasing concentrations of new CPP (10^–10^ to 10^–4^ M) or with the vehicle (methanol) for 30 min at 37 °C. Then cells were plated (500 000/well) on coated wells and incubated for 30 min at 37 °C. After three washes, adhered cells were lysed with 0.5% Triton X-100 in PBS (30 min at 4 °C) and fluorescence was measured (Ex485 nm/Em535 nm) in an EnSpire Multimode Plate Reader (PerkinElmer, Waltham, MA). For adhesion assays mediated by α_5_β_1_ integrin, 96-well plates were coated by passive adsorption with FN (10 μg/mL) overnight at 4 °C. K562 cells were counted and preincubated with various concentrations of the peptides or with the vehicle (methanol) for 30 min at RT. Afterward, the cells were plated (50 000 cells/well) and incubated at RT for 1 h. The wells were then washed with 1% BSA in PBS (phosphate-buffered saline) to take off nonadherent cells, and 50 μL of hexosaminidase substrate was added; after addition of 100 μL of stopping solution, the plates were read at 405 nm. In both types of adhesion assay, the number of adherent cells was determined by comparison with a standard curve made in the same plate. Experiments were carried out in quadruplicate and repeated at least three times. Data analysis and EC_50_ or IC_50_ values were calculated using GraphPad Prism 5.0 (GraphPad Software, San Diego, CA), and concentration–response curves are provided in SI Figures S2–S7. In addition, to depict agonistic or antagonistic behavior of the new synthesized compounds, we calculated the adhesion index (Figure 2), which is calculated as the ratio between the number of adhered cells in the presence of the highest CPP concentration (10^–4^ M) and the number of adhered vehicle-treated cells. On the basis of the adhesion index value, it is possible to distinguish between the following: agonist (adhesion index >1, cell adhesion in increased), antagonist (adhesion index <1, cell adhesion is decreased), compounds not significantly modifying integrin-mediated cell adhesion (adhesion index approximately = 1, cell adhesion is not significantly altered).

#### Competitive Binding Assay on Purified Integrins

Solid-phase ligand binding assays on purified integrin were conducted as previously described^34^ with the following modifications. Regarding α_5_β_1_ and α_L_β_2_ integrins, black 96-well plates were coated by passive adsorption with FN (0.5 μg/mL) for α_5_β_1_ or with ICAM-1 (10 μg/mL, R&D Systems) for α_L_β_2_ in carbonate buffer (15 mM Na_2_CO_3_, 35 mM NaHCO_3_, pH 9.6) overnight at 4 °C. The following day, wells were blocked with TSB buffer (20 mM Tris-HCl, 150 mM NaCl, 1 mM CaCl_2_, 1 mM MgCl_2_, 1 mM MnCl_2_, pH 7.5, 1% BSA) for 1 h at room temperature. Purified α_5_β_1_ (10 μg/mL) or α_L_β_2_ (7 μg/mL) was incubated with the new synthesized compounds, at different concentrations (10^–4^ to 10^–10^ M), in coated wells for 1 h at RT. Then, after three washes with PBST buffer, primary antibody (anti-α_5_β_1_, BD Bioscience, 1:100 dilution or anti-α_L_, Abcam, 1:200 dilution) was added for 1 h at RT. Then antirabbit AlexaFluor488-secondary antibody (ThermoFisher Scientific, 1:400 dilution) was added after three washes with PBST buffer and incubated 1 h at room temperature. After washing three times, fluorescence was measured (Ex485 nm/Em535 nm) in Multimode Plate Reader (PerkinElmer).

For the evaluation of binding affinity to purified α_4_β_1_, α_M_β_2_, and α_4_β_7_ integrins, competitive solid-phase ligand binding assays were performed as follows. Black 96-well plates were coated overnight at 4 °C with the following endogenous ligands: FN or VCAM-1 (10 μg/mL) for α_4_β_1_, MAdCAM-1 (2 μg/mL) for α_4_β_7_, and fibrinogen (10 μg/mL) for α_M_β_2_, in PBS+ 2 mM MgCl_2_ + 0.5% BSA. Afterward, each well was washed and blocked for 1 h at RT. Purified integrins (α_4_β_1_: 0.5 μg/mL; α_4_β_7_: 0.5 μg/mL; α_M_β_2_: 0.5 μg/mL; R&D Systems) were preincubated with serial dilutions of new compounds (10^–4^ – 10^–10^ M) for 30 min at RT and then plated into coated wells for 1 h at RT. After two washes, primary antibody (for α_4_β_1_ and α_4_β_7_: rabbit anti-α_4_, Abcam, 1:100 dilution; for α_M_β_2_: rabbit anti-α_M_, Abcam, 1:100 dilution) was added and incubated for 1 h at RT. The plate was washed twice and then was incubated with anti-rabbit AlexaFluor488 secondary antibody (1:400 dilution, ThermoFischer Scientific) for 1 h at RT. After washing three times, fluorescence was measured as described in the previous section.

Experiments were carried out in triplicate and repeated at least three times. Data analysis and IC_50_ affinity values were calculated using GraphPad Prism 9 (GraphPad Software), and binding curves are shown in Supporting Information, Figures S8–S12.

#### Western Blot Analysis

Western blot analysis was performed as previously described, with the following modifications. Jurkat E6.1 cells were cultured for 16/18 h in RPMI medium containing 1% FBS; then 4 × 10^6^ cells were incubated for 1 h with different concentrations of the most effective cyclic peptides (10^–7^, 10^–8^, 10^–9^ M), which were identified as agonists in cell adhesion assays mediated by α_4_β_1_ integrin. On the other hand, after 1 h incubation with integrin antagonists, the cells were then seeded on FN (10 μg/mL) coated plates for 1 h. Integrin agonists were not incubated with FN. At the end of the incubation time, Jurkat E6.1 cells were lysed on ice using a mammalian protein extraction reagent (M-PER; Pierce, Rockford, IL) supplemented with a phosphatase inhibitor cocktail. Protein extracts were quantified using a BCA protein assay kit (Pierce), separated by 12% SDS-PAGE gel, transferred onto nitrocellulose membranes, and immunoblotted with anti-phospho-ERK1/2 (1:1000) (Cell Signaling Technology, Danvers, MA) or anti-total ERK1/2 antibodies (1:2500) (Cell Signaling Technology). Protocols for digital image acquisition and analysis have been previously described.^73^ Densitometric analysis of the bands is reported (mean ± SD; *n* = 3); the amount of phosphorylated ERK1/2 (pERK1/2) is normalized to that of total ERK1/2 (totERK1/2). Experiments were replicated independently at least three times. Statistical analyses were performed using one-way ANOVA and the post hoc Newman–Keuls test.

#### In Vitro Enzymatic Stability

Enzymatic stability tests were carried out in triplicate and repeated three times using mouse serum (Sigma-Aldrich). Peptides was dissolved in Tris buffer pH 7.4 to a 10 mM concentration, and 10 mL aliquots were added to 190 mL of serum. Incubations were maintained at 37 °C, and 20 mL aliquots were sampled from the incubation mixtures at the indicated times of 0, 0.15, 0.5, 1.0, 2.0, and 3.0 h. Samples were diluted with 90 mL of CH_3_CN, and enzymatic activity was definitively stopped by adding 90 mL of 0.5% AcOH. After centrifugation (13 000*g* for 20 min), the supernatants were separated and the amount of remaining peptide was assessed by RP HPLC.

#### Conformational Analysis of CPPs

Peptide samples were dissolved in 8:2 DMSO-*d*_6_/H_2_O in 5 mm tubes to the final concentration of 0.01 M. At this concentration, the intramolecular aggregation in mixtures of DMSO-*d*_6_ and H_2_O is usually unimportant. Furthermore, self-association of the peptides was excluded based on the reproducibility of the chemical shift of nonexchangeable protons in the concentration range 0.01–0.04 M (not shown). Water suppression was achieved by the PRESAT procedure implemented in Varian. Proton resonance assignment was accomplished through gCOSY. VT ^1^H NMR experiments were recorded over the range of 298–348 K; temperature calibration was done with the ethylene glycol HO–CH_*n*_ chemical shift separation method. 2D ROESY experiments were done at RT, phase-sensitive mode, spin-locking field (γb2) = 2000 Hz, mixing time = 250 ms; spectra were processed in the hypercomplex approach; peaks were calibrated on the solvent. Only ROESY-derived constraints were included in the restrained molecular dynamics (MD). Cross-peak intensities were ranked and associated with the distances (Å): very strong = 2.3, strong = 2.6, medium = 3.0, weak = 5.0. The intensities of the cross-peaks arising from protons separated by known distances (e.g., geminal) were found to match with these associations but were discarded. For the absence of Hα(*i*), Hα (*i* + 1) ROESY cross-peaks, all of the ω bonds were set at 180° (*f* constant: 16 kcal mol^–1^ Å^–2^).

#### Molecular Dynamics Simulations

The restrained MD simulations were conducted at 300 K and 1 atm by using the AMBER force field in a 30 × 30 × 30 Å^3^ box of standard TIP3P models of equilibrated water, periodic boundary conditions dielectric scale factor = 1, and cutoff for the nonbonded interactions = 12 Å; all water molecules closer than 2.3 Å to a solute atom were eliminated, and 50 random structures were generated by a 100 ps simulation at 1200 K; these were subsequently subjected to restrained MD, 50 ps with a 50% scaled force field at 1200 K and then by 50 ps with full distance restraints, force constant = 7 kcal mol^–1^ Å^–2^, after which the system was cooled in 20 ps to 50 K. H-bond interactions were not included nor were torsion angle restraints. The resulting structures were minimized by 3000 cycles of steepest descent and 3000 cycles of conjugated gradient, and convergence = 0.01 kcal Å^–1^ mol^–1^. The backbones of the structures were clustered by the rmsd analysis. Unrestrained MD simulations were performed starting with the conformation derived from ROESY in the box of standard TIP3P water for 100 ns at 298 K using periodic boundary conditions, at constant temperature and pressure (Berendsen scheme, bath relaxation constant of 0.2). For 1–4 scale factors, van der Waals and electrostatic interactions are scaled in AMBER to half their nominal value. The integration time step was set to 0.1 fs. The system coordinates were collected every picosecond.

#### Molecular Modeling

The ligand molecules were obtained using a systematic conformational search followed by geometry optimization of the lowest energy structure with MOPAC7 (PM3Method, RMS gradient 0.01).^74^ Because the precise structure of the α4β1 integrin is not yet available, the α4β1 integrin receptor model was obtained by combining the crystallographic structures of the α4 subunit (PDB ID: 3V4V) and of the β1 subunit (PDB ID: 4WK4). This decision was made considering the better homology of the pair β1/β7 (52.80%) compared to α4/α5 (34.22%). The structural superposition was obtained using the “MatchMaker” procedure implemented in UCSF-Chimera.^75^ The pairwise sequence alignments of the protein fragments were achieved using the blocks substitution matrix 62 (BLOSUM-62) by the Needleman–Wunsch algorithm.^76^ The coordinates of the subunits were aligned using residue pairs from the sequence alignments. The superposition/alignment steps were iterated until convergence to perform one or more cycles of refitting of the structures using the sequence alignment and generating a new sequence alignment from the adjusted superposition. The residues at the α4β1 interface were checked and any clashes/overlaps were removed using the Dunbrack 2010 rotamer library, a backbone-dependent rotamer library composed of rotamer frequencies, mean dihedral angles, and variances as a function of the backbone dihedral angles.^77^ Hydrogen atoms were added with respect to the hydrogen bonding network by Reduce software,^78^ and the PROPKA program^79,80^ was employed to estimate the protonation states of the titratable residues. The final model (Supporting Information) was then validated using the ligands present in the two crystallographic structures used as models (RO0505376 in α4β7 and the cRGD peptide in α5β1). Even considering the obvious differences due to the new combination of the subunits, the complexes resulting from the molecular docking simulations are consistent with the conformations of the original complexes. Molecular docking experiments were performed with Autodock 4.0. We used the Lamarckian Genetic Algorithm which combines global search (Genetic Algorithm alone) to local search (Solis and Wets algorithm). Ligands and receptors were further processed using the Autodock Tools (ADT) software.^81^ Gasteiger PEOE^82^ charges were loaded on the ligands in ADT, and solvation parameters were added to the final structure using the Addsol utility of Autodock. Each docking run consisted of an initial population of 100 randomly placed individuals, a maximum number of 200 energy evaluations, a mutation rate of 0.02, a crossover rate of 0.80, and an elitism value of 1. For the local search, the so-called pseudo-Solis and Wets algorithm was applied using a maximum of 250 iterations per local search; 250 independent docking runs were carried out for each ligand. The grid maps representing the system in the actual docking process were calculated with Autogrid. The dimensions of the grids were 100 × 100 × 100, with a spacing of 0.1 Å between the grid points and the center close to the cavity left by the ligand after its removal. The simpler intermolecular energy function based on the Weiner force field in Autodock was used to score the docking results. Results differing by less than 1.0 Å in positional root-mean-square deviation (rmsd) were clustered together and were represented by the result with the most favorable free energy of binding. The poses thus obtained were equilibrated by a 5.0 ns of partially restrained MD simulation using the CUDA version of the GROMACS package^83^ with a modified version of the AMBER ff03 force field, a variant of the AMBER ff991 potential in which charges and main-chain torsion potentials have been derived based on QM+continuum solvent calculations and each amino acid is allowed unique main-chain charges. AmberTools^84,85^ was applied to generate the Generalized Amber Force Field (GAFF) files for the unusual residues. The GROMACS molecular topology files (*.gro and *.top) were obtained from the Amber files by Acpype.^86^ The MD consisted of 100 ps heating dynamics from 0 to 300 K, followed by equilibration dynamics performed for 5 ns. The MD simulation was performed at constant temperature and volume, with the application of constrained harmonic potentials for the metal ions. After the above-described MD simulations, a combined QM/MM calculation between the ligand and the protein environment was performed using the NWChem 6.1.1 package.^87^ The QM region contained the ligand atoms, the MIDAS, and the side chains of all major residues of the binding site. The theoretical level used for the QM region was the hybrid DFT of the B3LYP^88^ exchange-correlation functional with Grimme’s D3 dispersion correction (B3LYP-D3) and the 6-31G(d) basis sets while the MM atoms were subjected to an Amber ff99 force field (B3LYP-D3/6-31G(d) | Amber ff99). Hydrogen link atoms were used for the QM/MM boundary, and the nonbonded QM/MM interactions were calculated with a cutoff of 10 Å. Interactions of QM atoms with all MM charges were included in calculations.

## Supporting Information Available

Synthetic procedures, enzymatic stability in mouse serum, cell culture, concentration–response curves, VT-NMR experiments, ROESY cross peaks, α_4_β_1_ receptor model, views of PDB 3V4V (PDF)
PDB file of **3a** (PDB)
PDB file of **3b** (PDB)
PDB file of **3c** (PDB)
PDB file of **3d** (PDB)
PDB file of **12a** (PDB)
PDB file of **12c** (PDB)
PDB file of **15** (PDB)
CSV molecular formula strings (CSV)

## Supplementary Material

jm2c02098_si_001.pdf

jm2c02098_si_002.pdb

jm2c02098_si_003.pdb

jm2c02098_si_004.pdb

jm2c02098_si_005.pdb

jm2c02098_si_006.pdb

jm2c02098_si_007.pdb

jm2c02098_si_008.pdb

## Abbreviations Used

ADMIDAS: adjacent to MIDAS site
AE: autoimmune encephalitis
Amp: 4-amino-l-proline residue
BBB: blood–brain barrier
BSA: bovine serum albumin
CPP: cyclopentapeptide
CS1: connecting segment 1
DFT: density-functional theory
ECM: extra cellular matrix
Fg: fibrinogen
FN: fibronectin
IDS: Ile-Asp-Ser
LDT: Leu-Asp-Thr
LDV: Leu-Asp-Val
MAdCAM-1: mucosal vascular addressin cell adhesion molecule-1
MD: molecular dynamics
MIDAS: metal ion-dependent adhesion site
MPUPA: *o-*methylphenylureaphenylacetic acid
MS: multiple sclerosis
Phg: phenylglycine
Phu: phenylalanine-urea
PRESAT: solvent presaturation
SyMBS: synergistic metal ion binding site
VCAM-1: vascular cell adhesion molecule-1
VT: variable temperature
