# Supplementary material for: Design and Pharmacological Characterization of α4β1 Integrin Cyclopeptide Agonists: Computational Investigation of Ligand Determinants for Agonism versus Antagonism
Source: J Med Chem. 2023 Mar 28;66(7):5021–40. doi: 10.1021/acs.jmedchem.2c02098 (PMC10108353; doi:10.1021/acs.jmedchem.2c02098)
Supplement: Supplementary file 1 — jm2c02098_si_001.pdf [file jm2c02098_si_001.pdf]

Supporting Information

Design and pharmacological characterization of $\alpha_4\beta_1$ integrin cyclopeptide agonists: Computational investigation of ligand's determinants for agonism versus antagonism

Michele Anselmi,[†,//] Monica Baiula,[‡,//] Santi Spampinato,[‡] Roberto Artali,[§] Tingting He,[†] Luca Gentilucci,[†,¥,]*

[†] Department of Chemistry "G. Ciamician", University of Bologna, Via Selmi 2, 40126 Bologna, Italy

[¥] Health Sciences & Technologies (HST) CIRI, University of Bologna, Via Tolara di Sopra 41/E, 40064 Ozzano Emilia, Italy.

[‡] Department of Pharmacy and Biotechnology, University of Bologna, Via Irnerio 48, 40126, Bologna, Italy

[§] Scientia Advice, 20832 Desio, Monza and Brianza, Italy

*E-mail: luca.gentilucci@unibo.it

Table of contents

Chemistry pS2
Figure S1, enzymatic stability in mouse serum pS4
Table S1 .... pS5
Cell culture pS6
Figures S2-S7, cell adhesion concentration-response curves pS6
Figures S8-S12, solid-phase binding curves pS9
Figure S13, correlation between adhesion assay-determined potency and ligand binding affinity of LDV CPPs. pS12
VT NMR experiments pS12
Table S2, Δδ/Δt values pS13
Tables S3-S7, ROESY cross peaks pS13
Figure S14, views of PDB 3V4V pS18
Figures S15-S21, molecular modelling extras pS19
Figures S22-S33, ^{1}H and ^{13}C NMR spectra pS23
Figure S34, HPLC analyses pS35

Chemistry

(S)-, (R)-Fmoc-Asp-OBn, (S)-4, (R)-4. A mixture of either (S) or (R)-Fmoc-Asp(OtBu)-OH (1.0 mmol), tetra-butylammonium bromide (1.0 mmol), and anhydrous K_2CO_3 (1.1 mmol) in acetonitrile (5 mL) was stirred at RT for 20 min. A solution of benzyl bromide (1.1 mmol) in acetonitrile (4 mL) was then added dropwise under vigorous stirring. The mixture was stirred for 12 h at RT, then the precipitate was filtered off, and the filtrate was evaporated to dryness. The resulting crude material was dissolved in EtOAc, and the organic layer was washed three times with sat. $NaHCO_3$, H_2O and brine, dried over Na_2SO_4, and finally the solvent was evaporated at reduced pressure to afford the product as a white solid, used without further isolation. (*S*)-Fmoc-Asp(OtBu)-OBn (98%, 85% pure as determined by RP HPLC, General Methods). ESI MS *m/z* calcd. for $[C_{30}H_{31}NO_6Na]^+$ 524.2, found 524.2 $[M+Na]^+$. (*R*)-Fmoc-Asp(OtBu)-OBn (94%, 90% pure), ESI MS *m/z* calcd. for $[C_{30}H_{31}NO_6Na]^+$ 524.2, found 524.2 $[M+Na]^+$.

Either (*S*)- or (*R*)-Asp(OtBu)-OBn were treated with 25% TFA in DCM (4 mL) at 0°C under stirring for 3 h. The solvent was distilled at reduced pressure, then ice-cold water was added to the residue and the suspension was allowed to stir overnight. The resulting precipitate was collected by filtration, dried under high *vacuum* and used without further purifications. (*S*)-**4** (98%, 89% pure as determined by RP HPLC, General Methods), ESI MS *m/z* calcd. for $[C_{26}H_{24}NO_6]^+$ 446.2, found 446.2 $[M+H]^+$. (*R*)-**4** (95%, 92% pure), ESI MS *m/z* calcd. for $[C_{26}H_{24}NO_6]^+$ 446.2, found 446.3 $[M+H]^+$.

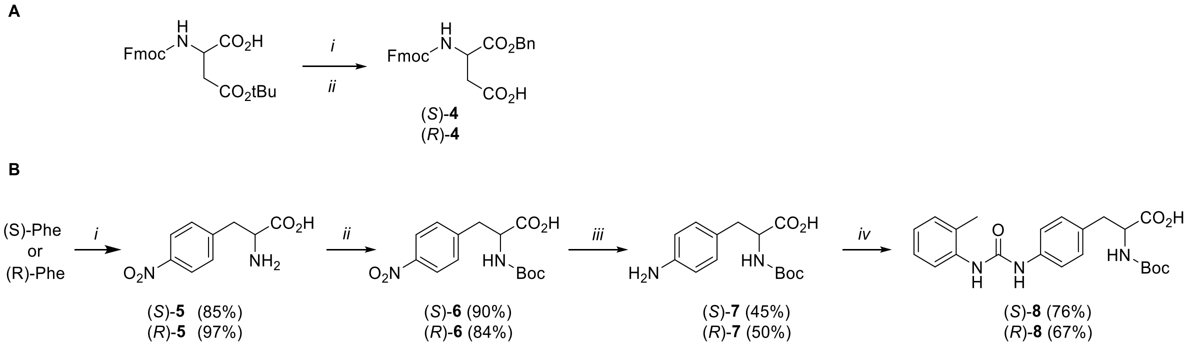

Scheme S1. (**A**) Preparation of (*S*)- or (*R*)-Fmoc-Asp-OBn, (S)-**4** or (*R*)-**4**. *Reagents and conditions: i*) benzyl-Br, Bu_4NBr, K_2CO_3; *ii*) TFA, DCM, RT. (**B**) Preparation of (*S*)- or (*R*)-Boc-Phu-OH (*S*)-**8** or (*R*)-**8**. *Reagents and conditions: i*) H_2SO_4, HNO_3, 0 °C, 1 h; *ii*) Boc_2O, Na_2CO_3, H_2O/dioxane, RT, 12 h; *iii*) H_2, Pd/C, MeOH, RT, 3 h; *iv) o*-tolyl isocyanate, DMF, RT, 3 h.

(S)-, (R)-4-NO$_2$-Phe, (S)-5, (R)-5. Phenylalanine (1.0 mmol) was added in small portion to conc. H_2SO_4 (6.0 mmol) until complete dissolution. The mixture was then cooled to 0°C, and conc. HNO_3 (0.65 mmol) was added dropwise. The reaction was stirred for 1 h at 0°C. Then, ice-cold H_2O (20 mL) was slowly added to the reaction mixture and stirred for additional 15 min. The mixture was heated to 100°C for 1 min and cooled to RT, then pH was corrected to 5-6 with 28% NH_4OH. The mixture was concentrated at reduced pressure and kept overnight for crystallization. The crystals were filtered, washed with water (5 mL) and dried under high *vacuum* to give (*S*)- or (*R*)-**5** as white solids. (*S*)-**5** (85%, 95% pure as determined by RP HPLC), ESI-MS *m/z* calcd. for $[C_9H_{11}N_2O_4]^+$ 211.1, found 211.2 $[M+H]^+$. (*R*)-**6** (97%, 94% pure), ESI-MS *m/z* calcd. for $[C_9H_{11}N_2O_4]^+$ 211.1, found 211.1 $[M+H]^+$.

(S)-, (R)-Boc-4-NO$_2$-Phe, (S)- 6, (R)-6. Boc_2O (1.2 mmol) was added to a suspension of (*S*)- or (*R*)-**5** (1.0 mmol) and Na_2CO_3 (2.0 mmol) in 1:1 H_2O/Dioxane (10 mL) at 0°C, and the mixture was stirred overnight at RT. Dioxane was distilled under reduced pressure, the alkaline aqueous layer was adjusted to pH 3-4 with 0.5 M HCl, then the mixture was extracted three times with EtOAc (10 mL). The combined organic phases were dried over Na_2SO_4, and the solvent was removed at reduced pressure, to afford (*S*)- or (*R*)-**6**, which were used without further purifications. (*S*)-**6** (90%, 90% pure as determined by RP HPLC, General

Methods), ESI-MS *m/z* calcd. for $[C_{14}H_{18}N_2O_6Na]^+$ 333.1, found 333.2 $[M+Na]^+$. (*R*)-**6** (84%, 90% pure), ESI-MS *m/z* calcd. for $[C_{14}H_{18}N_2O_6Na]^+$ 333.1, found 333.1 $[M+Na]^+$.

(S)-, (R)-Boc-4-NH$_2$-Phe, (S)-7, (R)-7. The nitro group of (*S*)- or (*R*)-**6** (1.0 mmol) was reduced by hydrogenation in MeOH in the presence of 10% *w/w* Pd/C while stirring for 3 h at RT. The mixture was filtered over a Celite® pad and the solvent was removed under reduced pressure. Then, Et_2O was added to the residue and the suspension was allowed to stir overnight. The resulting precipitate was collected by filtration, dried under high *vacuum* and used without further purifications: (*S*)-**7** (45%, 95% pure as determined by RP HPLC, General Methods), ESI MS *m/z* calcd. for $[C_{14}H_{21}N_2O_4]^+$ 281.1, found 281.1 $[M+H]^+$, 181.2 $[M\text{-}Boc+H]^+$. (*R*)-**7** (50%, 95% pure), ESI MS *m/z* calcd. for $[C_{14}H_{21}N_2O_4]^+$ 281.1, found 281.1 $[M+H]^+$.

*(S)-, (R)-Boc-Phu-OH, (S)-**8**, (R)-**8**.* *o*-Tolyl isocyanate (1.1 mmol) was added dropwise to a solution of either (*S*)-**7** or (*R*)-**7** (1.0 mmol) in DMF (3 mL) at RT under N_2 atmosphere. The mixture was stirred for 3 h, then ice-cold Et_2O (20 mL) was added and the precipitate (*S*)-**8** or (*R*)-**8** was collected as a brownish solid by filtration. (*S*)-**8** (76%, 95% pure as determined by RP HPLC, General Methods), ESI MS *m/z* calcd. for $[C_{22}H_{27}N_3O_5Na]^+$ 436.1, found 436.2 $[M+Na]^+$, 314.2 $[M\text{-}Boc+H]^+$. (*R*)-**8** (67%,95% pure), ESI MS *m/z* calcd. for $[C_{22}H_{27}N_3O_5Na]^+$ 436.1, found 436.2 $[M+Na]^+$, 314.1 $[M\text{-}Boc+H]^+$. ^{1}H NMR (400 MHz, DMSO*d$_6$*) σ 7.63 (d, 1H), 7.43 (d, 1H), 6.85-7.25 (m, 6H), 4.23 (m, 1H), 3.15 (dd, 1H), 2.9 (dd, 1H), 2.3 (s, 3H), 1.4 (s, 9H).

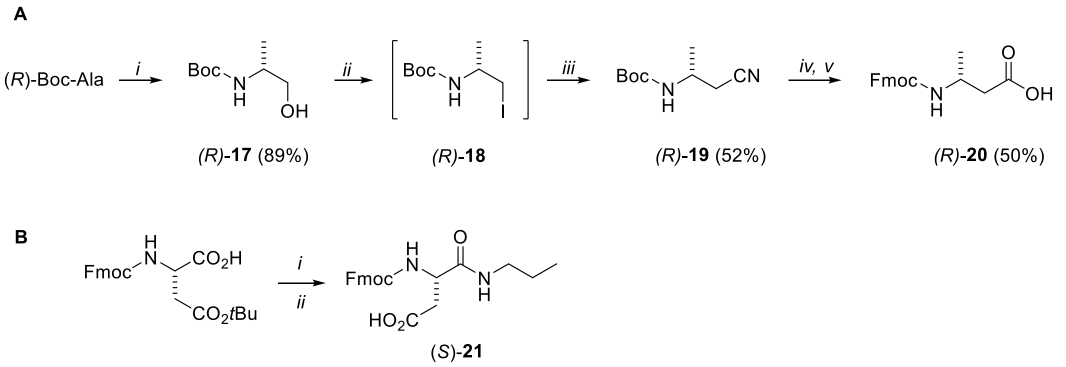

Scheme S2. (**A**) Preparation of (R)-*N*-Boc-β^3-homoAla **20**. *Reagents and conditions: i*) NMM, ethyl chloroformate, THF, 0°C - RT, 15 min, then $NaBH_4$, 0°C - RT, 10 min; *ii*) PPh_3, I_2, imidazole, DCM, reflux, 3 h; *iii*) KCN, DMSO, 60 °C, 4 h; *iv*) HCl 3M, reflux, 12 h; *v*) Fmoc-Cl, Na_2CO_3, in 1:1 H_2O/dioxane at RT. (**B**) Synthesis of Fmoc-(*R*)-Asp-propylamide. *Reagents and conditions: i*) *n*-propylamine, EDC·HCl, HOBt, TEA, DM1F/DCM, RT, 3 h; *ii*) TFA, DCM, RT, 1 h.

*(R)-tBu-(1-hydroxypropan-2-yl)carbamate, **17**.* To a stirred solution of NMM (1.1 mmol) and Boc-(*R*)-Ala-OH (1.0 mmol) in dry THF (5 mL), ethyl chloroformate (1.1 mmol) was added dropwise at 0 °C under inert atmosphere. After 15min, the solution was filtered and the precipitated was washed with THF (5 mL). The filtrates were collected, and a solution of $NaBH_4$ (1.25 mmol) in H_2O (5 mL) was then added dropwise at 0°C under stirring. The mixture was risen to RT, and after 10 min the solvent was distilled under reduced pressure. The residue was re-dissolved in EtOAc (30 mL) and the suspension was washed with H_2O and brine (5 mL each), then dried Na_2SO_4. The solvent was distilled under reduced pressure, giving **17** (89%) as a yellow oil, which was used without further purifications. ^{1}H-NMR (400 MHz, $CDCl_3$) δ 4.67 (br s, 1H, NH), 3.78-3.60 (m, 1H, CHα), 3.50 (dd, *J* = 13.8, 7.0 Hz, 1H, CHβ), 3.26 (dd, *J* = 12.4, 7.0 Hz, 1H, CHβ), 1.43 (s, 9H, tBu), 1.27 (d, *J* = 6.8 Hz, 3H, CH_3). ESI-MS *m/z* calcd. for $[C_8H_{18}NO_3]^+$ 176.1, found 176.2 $[M+H]^+$.

*(R)-tBu-(1-cyanopropan-2-yl)carbamate, **19**.* To a stirred solution of triphenylphosphine (1.25 mmol) in dry DCM (10 mL), I_2 (1.3 mmol) was added at RT under inert atmosphere. After 15 min, imidazole (2.5 mmol) was added and the mixture was stirred for additional 15 min. A solution of the crude **17** (1.0 mmol) in

dry DCM (5 mL), was added and the mixture was heated to reflux for 3 h. Then the mixture was cooled and diluted with DCM (40 mL), and washed with 10% aq $Na_2S_2O_5$ (20 mL) and brine (20 mL). The organic layer was dried over Na_2SO_4 and the solvent was evaporated at reduced pressure. The crude iodide **18** so obtained was then dissolved in dry DMSO (30 mL) and KCN (2.0 mmol) was added in one portion. The mixture was stirred under inert atmosphere at 60 °C for 4 h. The solution was then poured into water (10 mL), and the mixture was extracted twice with EtOAc (40 mL). The organic layer was washed with brine (20 mL) and dried over Na_2SO_4 and then evaporated at reduced pressure. The crude residue was purified by flash chromatography over silica gel (eluent cyclohexane/EtOAc 80:20) to give **19** (52 % over two steps).

*Fmoc-(R)-β^3-homoAla **20**.* The nitrile **19** (1.0 mmol) was dissolved in 6M HCl (10 mL) and heated to reflux for 12 h. The reaction mixture was then cooled at 0 °C and neutralized with 2M NaOH solution. The mixture was concentrated under reduced pressure to afford β^3-homoAla as HCl salt. The resulting salt was suspended in a mixture of 1:1 H_2O/dioxane (5 mL) and Na_2CO_3 (2.0 mol) and finally Fmoc-Cl (1.0 mmol) was added at 0 °C. The reaction was stirred at RT overnight, then the mixture was concentrated under reduced pressure, and the basic aqueous layer was adjusted to pH 3-4 with 0.5 M HCl, and the mixture was extracted three times with EtOAc (20 mL). The combined organic phases were dried over Na_2SO_4, and concentrated under reduced pressure. The residue was purified by flash chromatography over silica gel (eluent cyclohexane/EtOAc/AcOH 60:40:1) to afford **20** (55%). ESI-MS *m/z* calcd. for $[C_{19}H_{20}NO_4]^+$ 326.1, found 326.2 $[M+H]^+$.

*Fmoc-(R)-Asp-N-propylamide **21***. A mixture of Fmoc-(*R*)-Asp(OtBu)-OH (1.2 mmol), EDC·HCl (1.5 eq), HOBt (1.5 eq) and TEA (3.0 eq) in 3:1 DMF/DCM (5 mL) was stirred at RT for 10 min, then *n*-propylamine (1.5 mmol) was added, and the mixture was stirred under N_2 at RT for 3 h. Then the solvent was distilled at reduced pressure, and the residue was purified by flash chromatography over silica gel (eluent cyclohexane/EtOAc 70:30) to afford Fmoc-(R)-Asp(OtBu)-N-propylamide (69%). ^{1}H-NMR (400 MHz, $CDCl_3$) δ 7.78 (d, *J* = 7.5 Hz, 2H, ArH), 7.60 (d, *J* = 7.5 Hz, 2H, ArH), 7.42 (t, *J* = 7.5 Hz, 2H, ArH), 7.33 (t, *J* = 7.4 Hz, 2H, ArH), 6.47 (br.t, 1H, propylNH), 5.97 (d, *J* = 8.0 Hz, 1H, AspNH), 4.45 (d, *J* = 6.8 Hz, 2H, FmocCH_2), 4.23 (m, 1H, AspHα), 3.22 (q, *J* = 5.9 Hz, 2H, propylCH_2), 2.93 (dd, *J* = 17.0, 3.8 Hz, 1H, AspHβ), 2.60 (dd, *J* = 17.2, 6.8 Hz, 1H, AspHβ), 1.57-1.48 (m, 2H, propylCH_2), 1.46 (s, 9H, *t*-Bu), 0.91 (t, *J* = 7.4 Hz, 3H, propylCH_3). ESI-MS *m/z* calcd. for $[C_{26}H_{33}N_2O_5]^+$ 453.2, found 453.0 $[M+H]^+$. Removal of *t*Bu-protecting group was performed with 25% TFA in DCM (4 mL) at 0°C under stirring for 3 h. Thereafter the solvent was distilled at reduced pressure and the residue was triturated in Et_2O (10 mL), giving **21** in quantitative yield, directly used in the next step without further purifications. ESI-MS *m/z* calcd. for $[C_{22}H_{25}N_2O_5]^+$ 397.2, found 397.0 $[M+H]^+$.

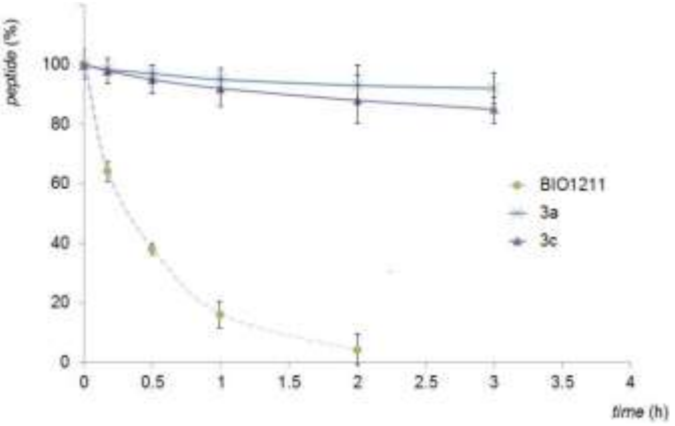

Figure S1

Degradation of BIO1211, **3a**, **3c** in mouse serum. Samples were collected from the incubation solution at the indicated times. Peptide stability was determined using an RP-HPLC ESI-MS analysis (described in the Methods). Values are presented as mean ± SD (n = 3).

Table S1. RP HPLC and ESI MS analyses of the linear precursors **9** and of the cyclopeptides **10**, and reaction yields.

**9**	Linear peptide sequence [a]	Yield (%) [b]	Purity (%) [c]	ESI MS $[M+1]^+$ found/calcd.[d]	**10**	Yield (%) [e]	Purity (%) [f]	ESI-MS $[M+1]^+$ found/calcd.[d]	CPP [g]
-	H-isoAsp(OBn)-Phu-Leu-Asp(OBn)-Val-OH	30	76	936.2/936.4 [h]	**a**	traces	nd	nd	**3a**
-	H-Asp(OBn)-Val-isoAsp(OBn)-Phu-Leu-OH	32	78	936.2/936.4 [h]	**a**	traces	nd	nd	**3a**
**a**	H-(*S*)-Phu-Leu-Asp(OBn)-Val-(*S*)-Asp-OBn	22	80	936.2/936.4 [h]	**a**	28	98	918.2/918.4 [i]	**3a**
**b**	H-(*S*)-Phu-Leu-Asp(OBn)-Val-(*R*)-Asp-OBn	24	81	936.2/936.4 [h]	**b**	19	97	918.2/918.4 [i]	**3b**
**c**	H-(*R*)-Phu-Leu-Asp(OBn)-Val-(*S*)-Asp-OBn	34	79	936.2/936.4 [h]	**c**	29	97	918.2/918.4[i]	**3c**
**d**	H-(*R*)-Phu-Leu-Asp(OBn)-Val-(*R*)-Asp-OBn	44	85	936.6/936.4 [h]	**d**	34	99	918.6/918.4 [i]	**3d**
**e**	H-(*S*)-Phu-Leu-Asp(OBn)-Val-(*R*)-β^3homoAla-OH	27	75	816.6/816.4 [j]	**e**	50	95	798.2/798.4 [k]	**11a**
**f**	H-(*S*)-Phu-Leu-Ala-Val-(*S*)-Asp-OBn	46	83	802.6/802.4 [l]	**f**	37	96	784.4/784.4 [m]	**12a**
**g**	H-(*S*)-Phu-Leu-Asp(OBn)-Val-(*S*)-Asp-nPr [a]	45	82	887.4/887.4 [n]	**g**	27	95	869.2/869.4 [o]	**13**
**h**	H-(*R*)-Phu-Leu-Asp(OBn)-Val-(*R*)-β^3homoAla-OH	47	78	816.6/816.4 [j]	**h**	33	98	798.2/798.4 [k]	**11c**
**i**	H-(*R*)-Phu-Leu-Ala-Val-(*S*)-Asp-OBn	40	79	802.6/802.4 [l]	**i**	15	97	784.2/784.4 [m]	**12c**
**j**	H-(*S*)-Phu-Phe-Asp(OBn)-Val-(*S*)-Asp-OBn	48	73	970.2/970.4 [p]	**j**	25	97	952.2/952.4 [q]	**14**
**k**	H-(*S*)-Phu-Phe-Ala-Val-(*S*)-Asp-OBn	36	85	836.2/836.4 [r]	**k**	48	98	818.2/818.4 [s]	**15**
**l**	H-(*R*)-Phu-Leu-Asp(OBn)-Phg-(*S*)-Asp-OBn	37	74	970.2/970.4 [p]	**l**	43	96	952.2/952.4 [q]	**16**

[a] isoAsp(OBn) corresponds to Asp-OBn; isoAsp(nPr) corresponds to Asp-nPr. [b] based on the estimated loading of the resin. [c] Determined by analytical RP HPLC (General methods) on a C18 RP column (100×3 mm, 3 µm, 110 Å), mobile phase from 9:1 H_2O/CH_3CN/0.1% HCOOH to 2:8 H_2O/CH_3CN/0.1% HCOOH in 20 min, flow rate of 1.0 mL min^{-1}. [d] MS single quadrupole HP 1100MSD detector. [e] Determined after semi-preparative RP HPLC (General methods) on a C18 RP column (21.2×150 mm, 7 µm 80 Å), mobile phase from 8:2 H_2O/CH_3CN to 100% CH_3CN in 10 min, flow rate 12 mL min^{-1} for CPPs **10a-i,** or on a C18 RP column (19×150 mm, 5 µm 130 Å), isocratic mobile phase 1:1 H_2O/CH_3CN/0.1% TFA in 8 min, followed by 100% CH_3CN in 5 min, flow rate 10 mL min^{-1} for CPPs **10j-l,** [f] Same stationary phase as for [c], mobile phase from 9:1 H_2O/CH_3CN to 2:8 H_2O/CH_3CN in 20 min, flow rate 1.0 mL min^{-1}. [g] Ester deprotection proceeded in nearly quantitative yield; purities are reported in Table 1 and 2. [h] Calcd for $[C_{50}H_{62}N_7O_{11}]^+$. [i] Calcd for $[C_{50}H_{60}N_7O_{10}]^+$. [j] Calcd for $[C_{43}H_{58}N_7O_9]^+$. [k] Calcd for $[C_{43}H_{56}N_7O_8]^+$. [l] Calcd for $[C_{42}H_{56}N_7O_9]^+$. [m] Calcd for $[C_{42}H_{54}N_7O_8]^+$. [n] Calcd for $[C_{46}H_{63}N_8O_{10}]^+$. [o] Calcd for $[C_{46}H_{61}N_8O_9]^+$. [p] Calcd for $[C_{53}H_{60}N_7O_{11}]^+$. [q] Calcd for $[C_{53}H_{58}N_7O_{10}]^+$. [r] Calcd for $[C_{45}H_{54}N_7O_9]^+$. [s] Calcd for $[C_{45}H_{52}N_7O_8]^+$. nd: not determined.

Cell culture. Jurkat E6.1 (expressing $\alpha_4\beta_1$ and $\alpha_L\beta_2$ integrin), K562 (expressing $\alpha_5\beta_1$ integrin) and HL60 (expressing $\alpha_M\beta_2$ integrin) cell lines were purchased from ATCC (Rockville, MD, USA); these cells were routinely cultured in RPMI-1640 (Life Technologies) supplemented with glutamine and 10% FBS. RPMI8866 cells were a kind gift from Prof. A. Santoni (Mayo Foundation for Medical Education and Research, Rochester, MN, USA); RPMI8866 cells were routinely grown in RPMI-1640 enriched with 10% FBS, 5 mM Hepes and 0.5 mM sodium pyruvate and usually kept in 50 mL of culture medium and allowed to form large clumps. Cells were grown at 37 °C under 5% CO_2 humidified atmosphere. 40 h before the adhesion assays, K562 and HL60 cells were treated with 25 nM or 40 nM phorbol 12-myristate 13-acetate (PMA; Sigma-Aldrich), respectively, to induced cell differentiation and to increased $\alpha_5\beta_1$ or $\alpha_M\beta_2$ integrin expression on cell surface (Baiula et al., 2016).

Cell adhesion assays. Concentration-response curves, obtained from cell adhesion assays performed in presence of increasing concentrations of LDV-CPPs, are shown in the following figures.

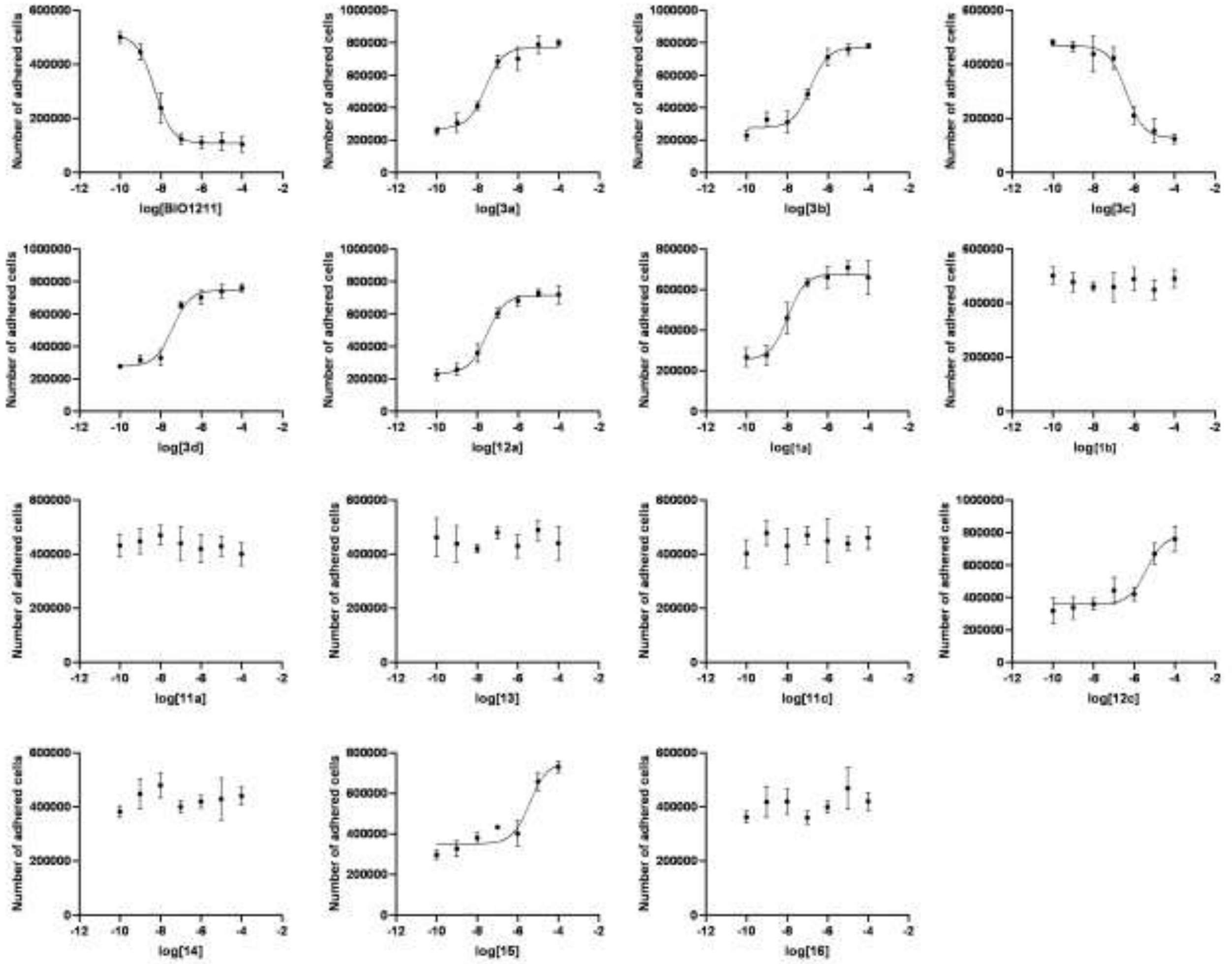

Figure S2 Concentration-response curves obtained from cell adhesion assays: evaluation of CPPs effects on $\alpha_4\beta_1$-mediated Jurkat cell adhesion to FN. Values represent the mean ± SD of three independent experiments carried out in quadruplicate.

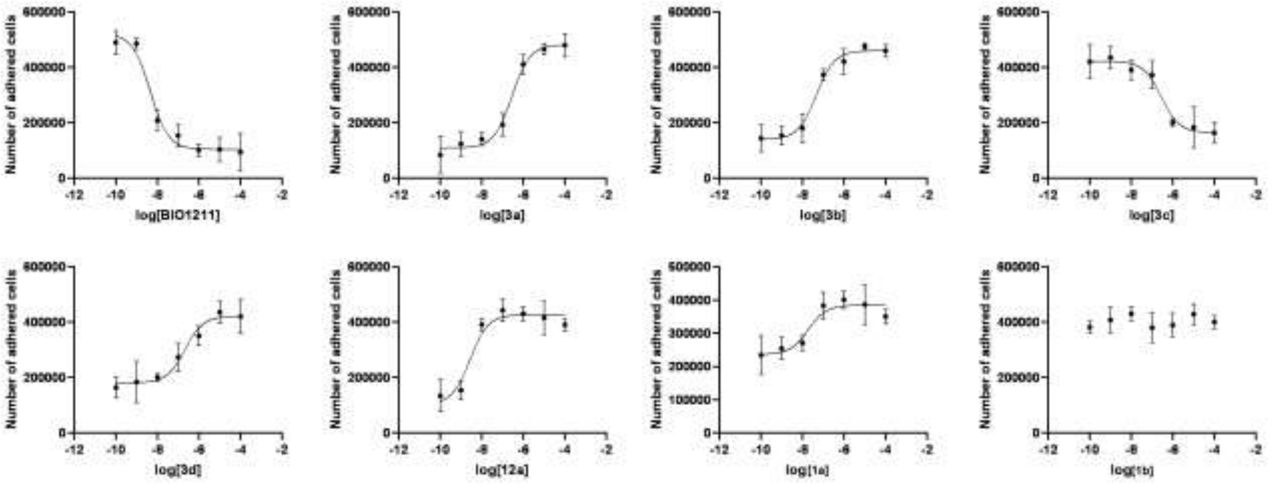

Figure S3 Concentration-response curves obtained from cell adhesion assays: evaluation of CPPs effects on $\alpha_4\beta_1$-mediated Jurkat cell adhesion to VCAM-1. Values represent the mean ± SD of three independent experiments carried out in quadruplicate.

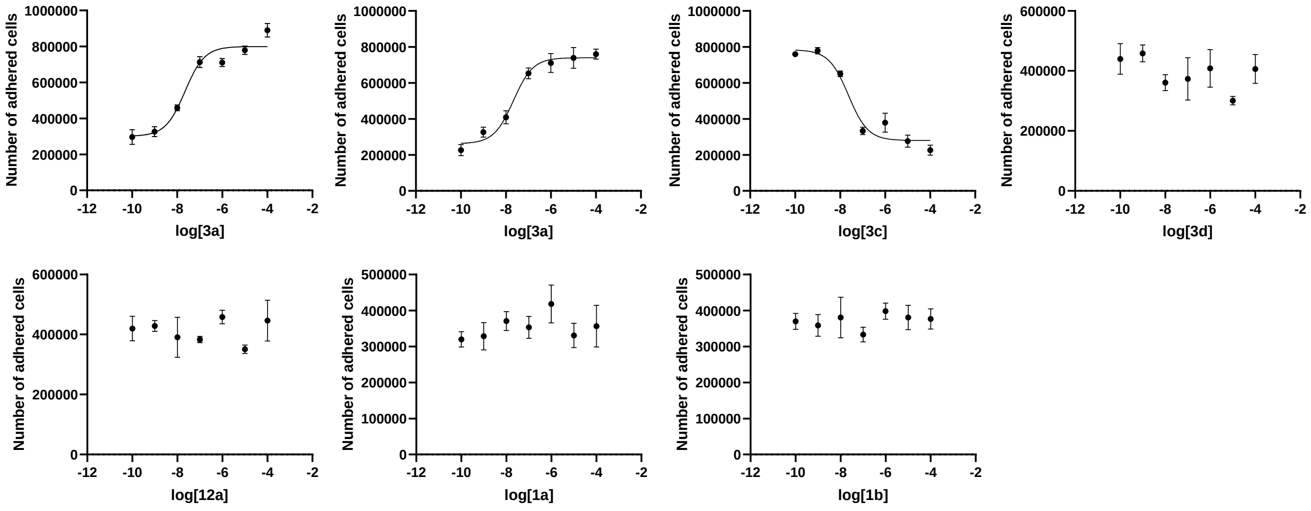

Figure S4 Concentration-response curves obtained from cell adhesion assays: evaluation of CPPs effects on $\alpha_4\beta_7$-mediated RPMI8866 cell adhesion to MAdCam-1. Values represent the mean ± SD of three independent experiments carried out in quadruplicate.

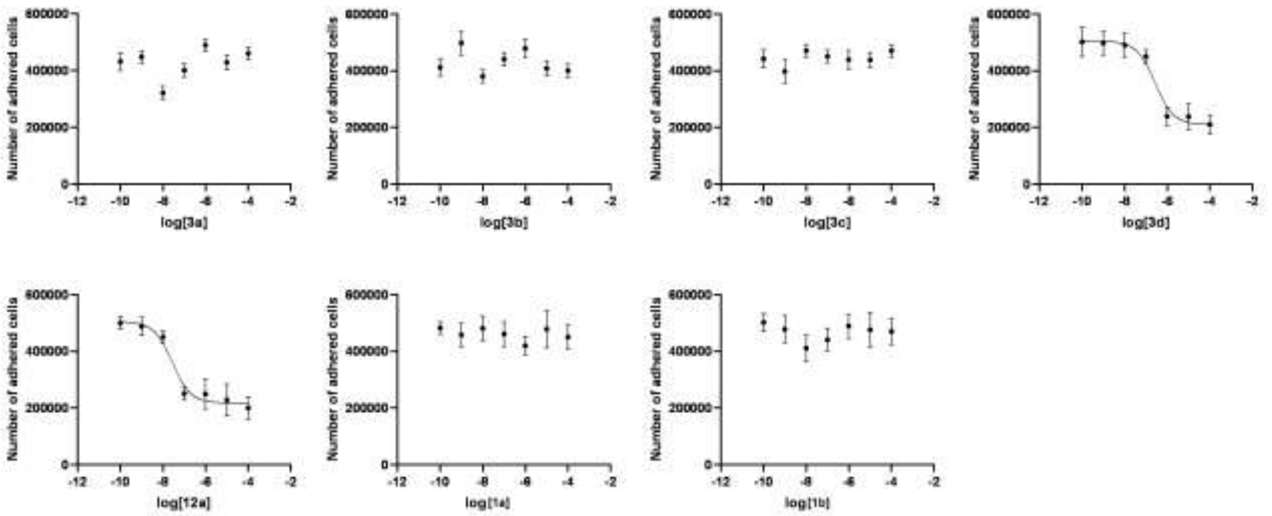

Figure S5 Concentration-response curves obtained from cell adhesion assays: evaluation of CPPs effects on $\alpha_M\beta_2$-mediated HL60 cell adhesion to FN. Values represent the mean ± SD of three independent experiments carried out in quadruplicate.

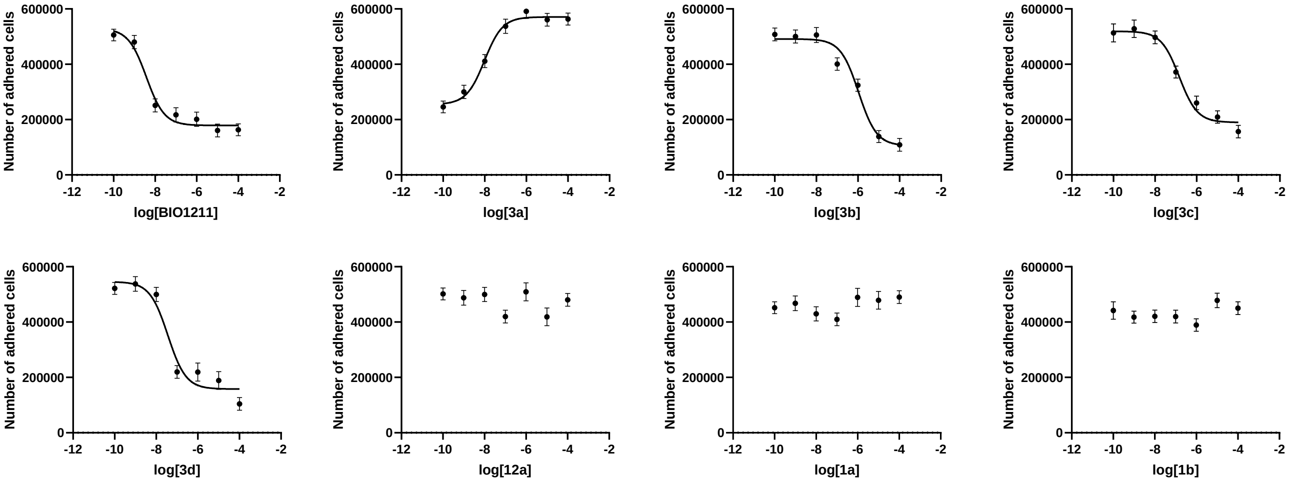

Figure S6 Concentration-response curves obtained from cell adhesion assays: evaluation of CPPs effects on $\alpha_L\beta_2$-mediated Jurkat cell adhesion to ICAM-1. Values represent the mean ± SD of three independent experiments carried out in quadruplicate.

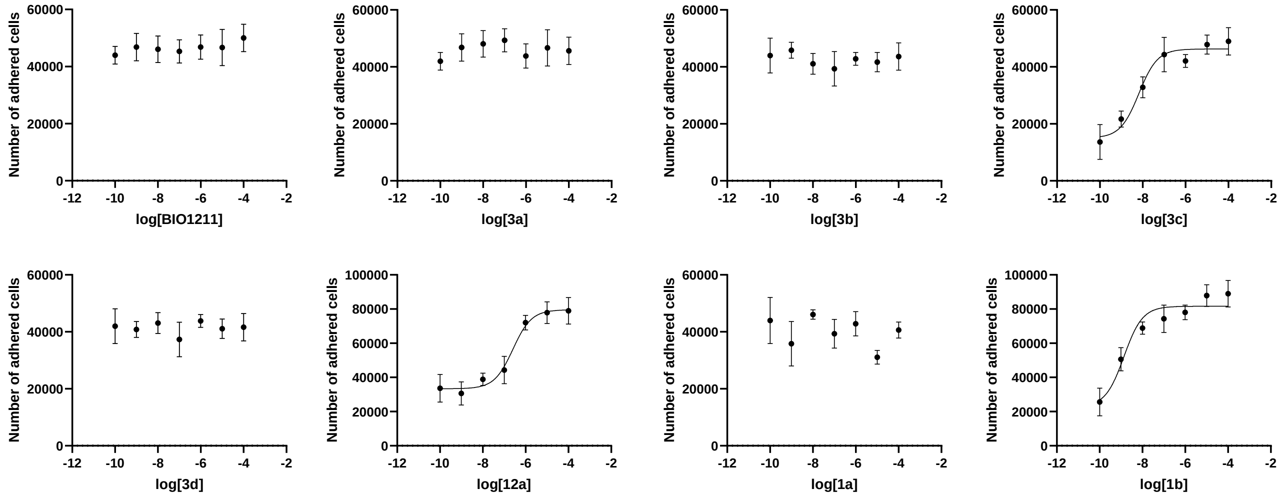

Figure S7 Concentration-response curves obtained from cell adhesion assays: evaluation of CPPs effects on $\alpha_5\beta_1$-mediated K562 cell adhesion to FN. Values represent the mean ± SD of three independent experiments carried out in quadruplicate.

Solid-Phase Binding Assays. Binding curves deriving from solid-phase binding assays are shown in the following figures.

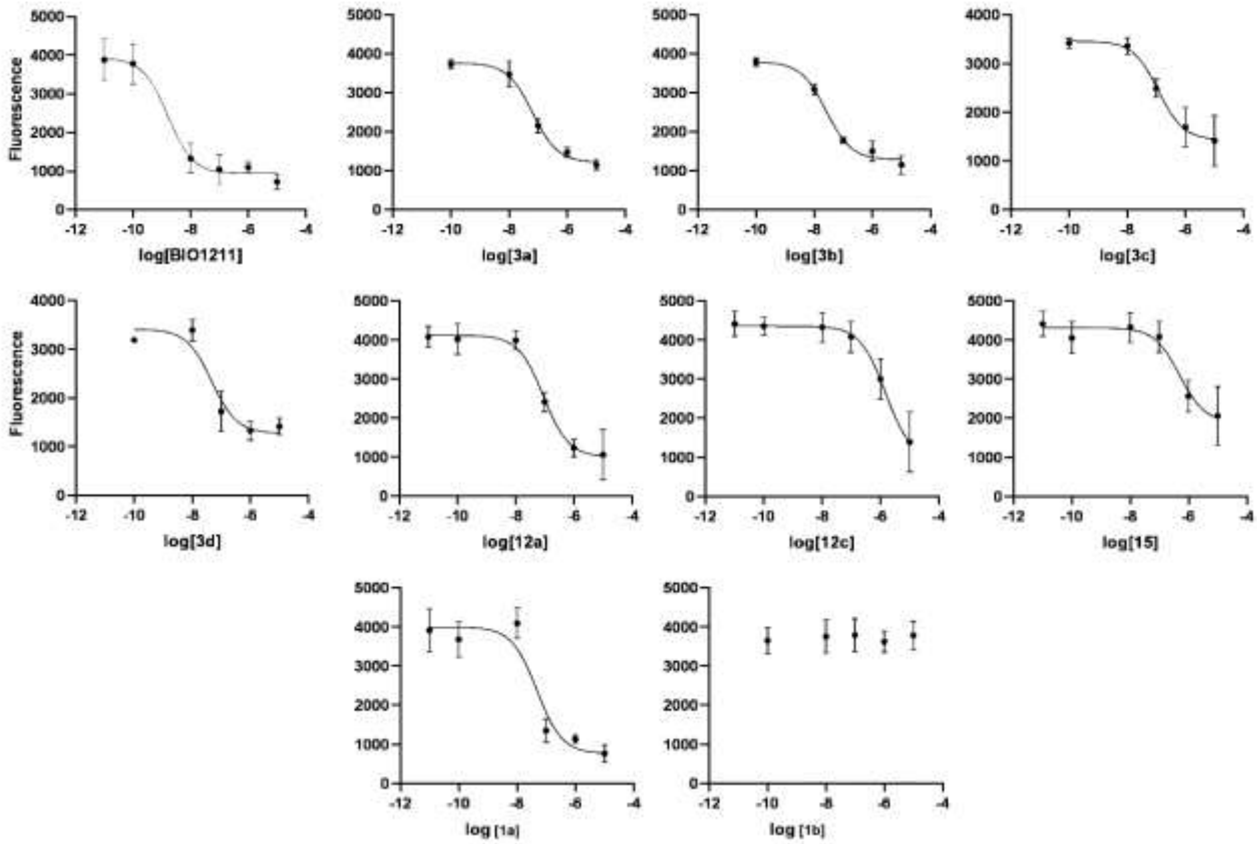

Figure S8 Binding assay curves for $\alpha_4\beta_1$/FN in presence of increasing concentrations of LDV CPPs. Values represent the mean ± SD of three independent experiments carried out in triplicate.

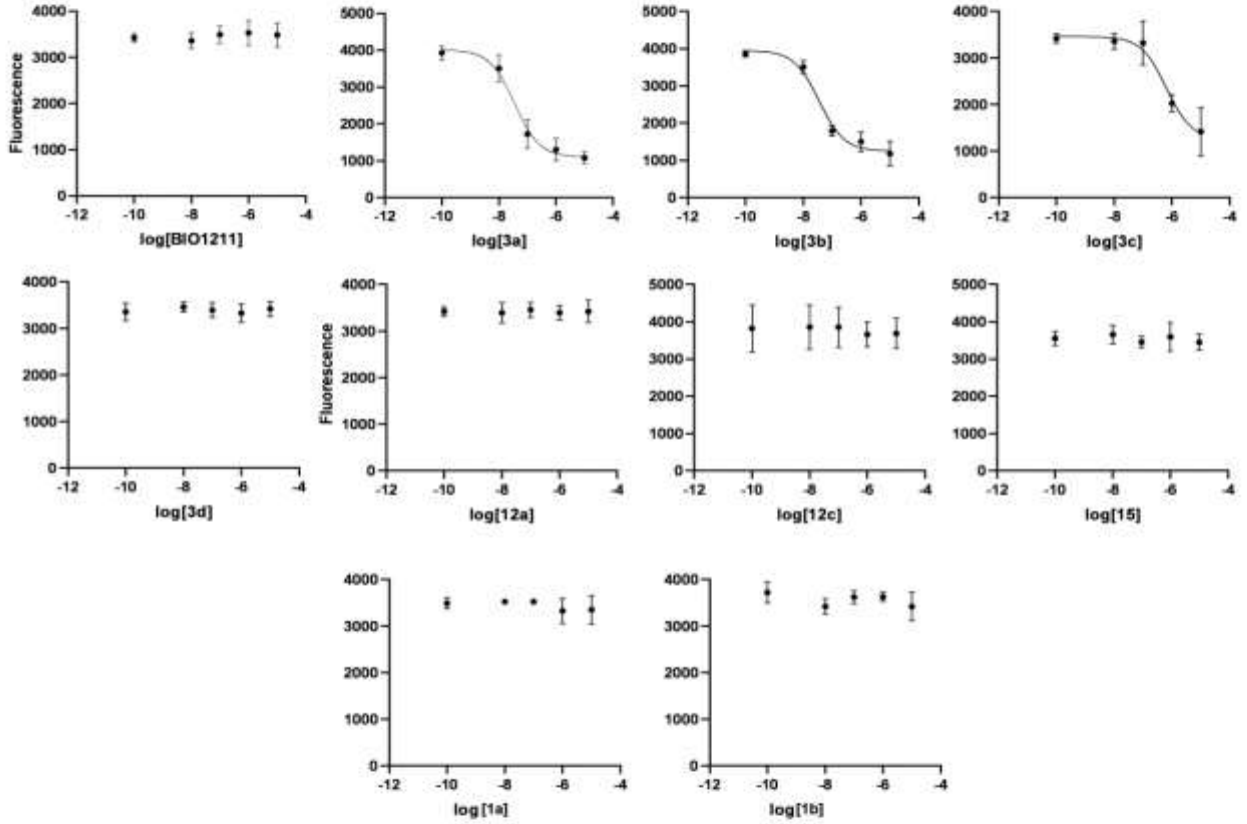

Figure S9 Binding assay curves for $\alpha_4\beta_7$/MAdCAM-1 in presence of increasing concentrations of LDV CPPs. Values represent the mean ± SD of three independent experiments carried out in triplicate.

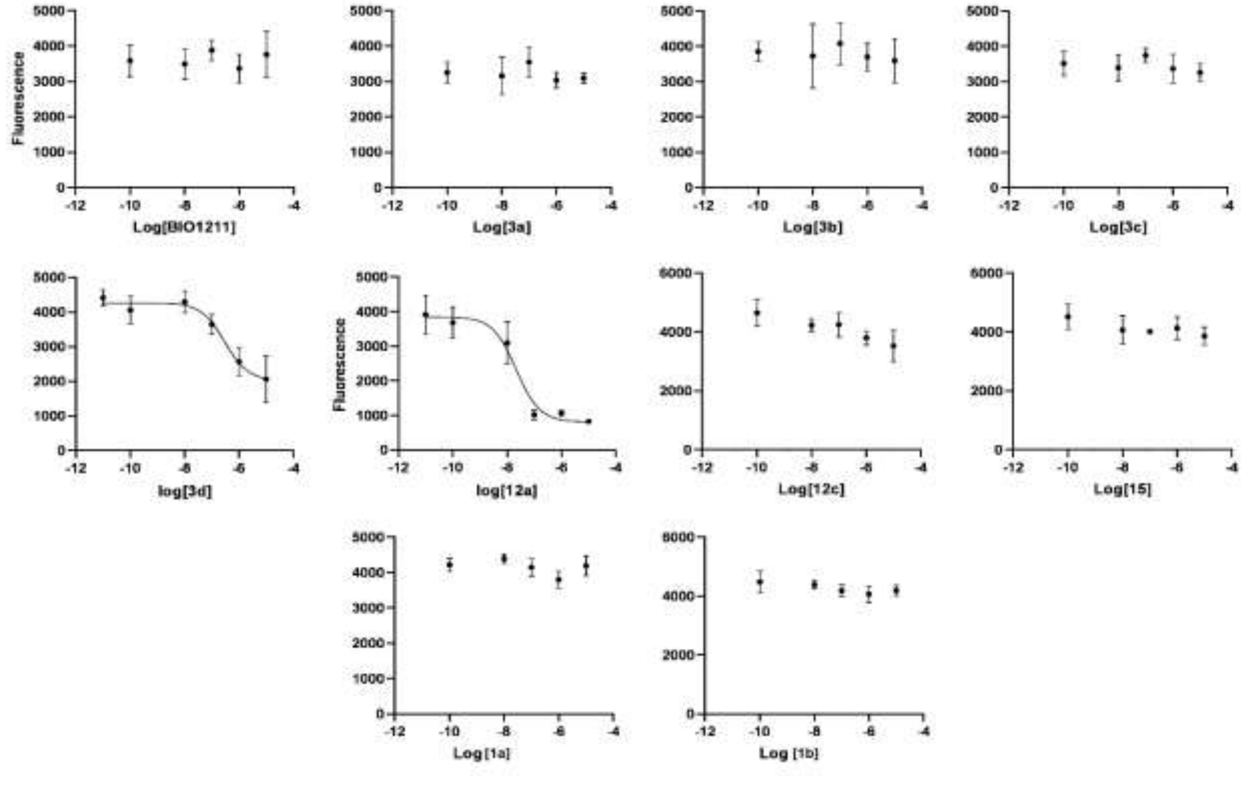

Figure S10 Binding assay curves for $\alpha_M\beta_2$/fibrinogen in presence of increasing concentrations of LDV CPPs. Values represent the mean ± SD of three independent experiments carried out in triplicate.

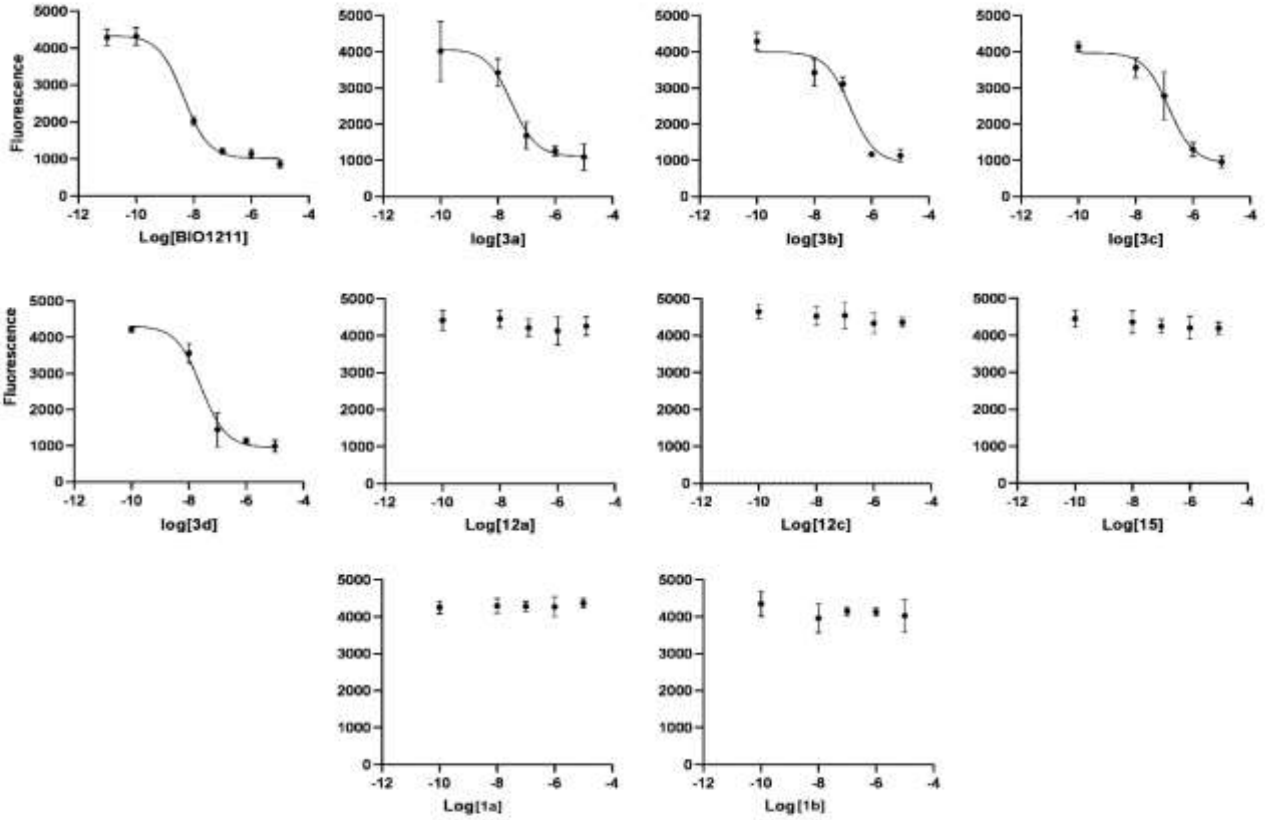

Figure S11 Binding assay curves for $\alpha_L\beta_2$/ICAM-1 in presence of increasing concentrations of LDV CPPs. Values represent the mean ± SD of three independent experiments carried out in triplicate.

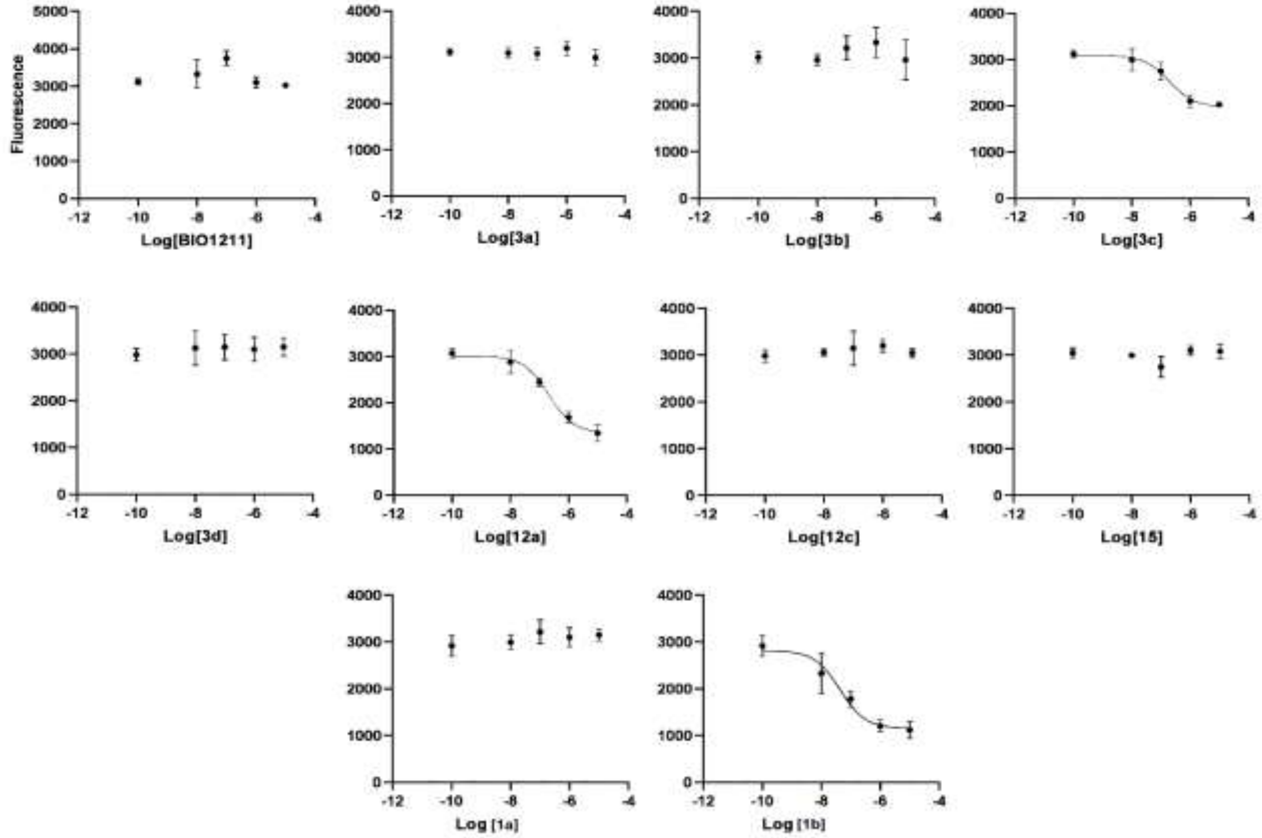

Figure S12 Binding assay curves for $\alpha_5\beta_1$/FN in presence of increasing concentrations of LDV CPPs. Values represent the mean ± SD of three independent experiments carried out in triplicate.

Correlation between adhesion assay-determined potency and ligand binding affinity of LDV CPPs.

The correlation was calculated on the basis of experimental data obtained for the LDV CPPs whose ligand binding affinities and potencies were obtained by solid-phase binding assays and cell adhesion assays, respectively. This evaluation was performed only for $\alpha_4\beta_1$/FN, $\alpha_4\beta_1$/VCAM-1 and $\alpha_L\beta_2$/ICAM-1 having sufficient active compounds to conduct a proper analysis.

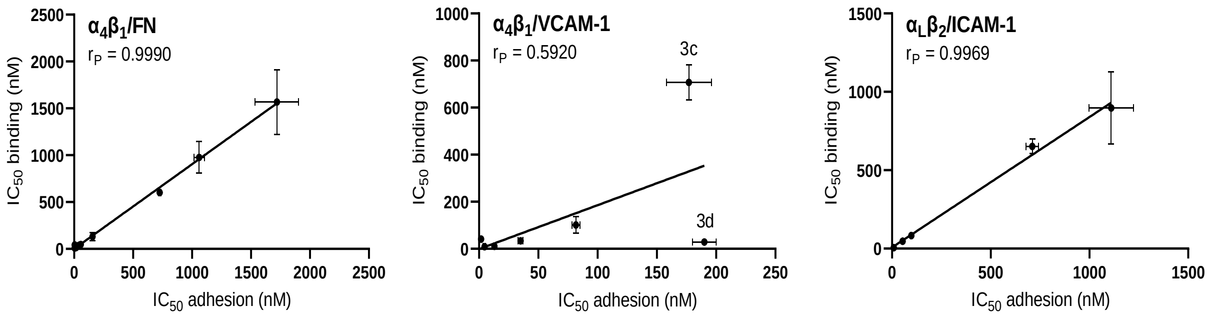

Figure S13 Relation between experimentally determined potencies (IC_{50}/EC_{50} deriving from cell adhesion assays) and ligand binding affinities (IC_{50} deriving from solid-phase binding assays) of the LDV CPPs. The IC_{50}/EC_{50} values ± S.E. are obtained by Tables 1-3. Correlation was measured using Pearson (r_P) correlation coefficient.

Variable temperature (VT) analysis of the CPPs. ^{1}H-NMR experiments in DMSO-d_6/H_2O (8:2) were recorded at diverse temperatures to determine if the amide protons were involved in intramolecular hydrogen bonding or were solvent exposed (Table S2). Generally, hydrogen bonded amide NH signals display comparatively lower Δδ/ΔT values, |Δδ/ΔT| < 2.0 ppb K^{-1}, as compared to solvent-exposed amide. Since the viscosity of the cryo-mixture is temperature-dependent, the conformational equilibrium could change at diverse temperatures, altering the resonance pattern. However, as showed by ^{1}H-NMR spectra, the CH resonances were perfectly maintained over the range of temperatures suggesting that the global conformations were not significantly altered. As reported in the Table, **3a** showed much lower |Δδ/ΔT| values for Val^{4}NH and isoAsp^{5}NH, suggesting that these amide protons could be involved in strong hydrogen bonds (|Δδ/ΔT| < 1 ppb K^{-1}). The analysis of **3b** (Table 2) supported the presence of weak hydrogen bonds on Val^{4}NH (Δδ/Δt = -1.9 ppb K^{-1}) and isoAsp^{5}NH (Δδ/Δt = -2.2 ppb K^{-1}). For **3c** (Table S2), the very scarce dependence of chemical shifts from temperature supported a strong hydrogen bond on Asp^{3}NH (Δδ/Δt = -0.3 ppb K^{-1}). Finally, the VT NMR parameters of **3d** led to predict a very strong hydrogen bond on Asp^{3}NH (Δδ/Δt = +0.4 ppb K^{-1}), and possibly a weaker hydrogen bonds for Phu^{1}NH and Leu^{2}NH (Δδ/Δt = -2.1 and -2.9 ppb K^{-1}, respectively).

The analysis of remaining CPPs showed the same trends of Δδ/Δt parameters as the parent peptides, suggesting that the hydrogen-bonding patterns and secondary structure elements were maintained. The CPPs **11a**, **12a** and **13**, showed comparatively lower |Δδ/ΔT| values for Val^{4}NH and for the amide proton of the β-amino acid at position 5, which is quite similar to the parent peptide **3a**. And the VT NMR parameters suggested the formation of extra hydrogen bonds involving Asp^{3}NH (Δδ/ΔT = -2.2 ppb K^{-1}), Ala^{3}NH (Δδ/ΔT = +1.6 ppb K^{-1}) and Phu^{1}NH (Δδ/ΔT = -1.7 ppb K^{-1}) in CPPs **11a**, **12a** and **13** respectively. The VT-NMR analyses of **11c** and **12c** confirmed the hydrogen bonding network as for the parent **3c**, albeit with slightly different Δδ/ΔT values (for **11c** Asp^{3}NH Δδ/ΔT = -0.7 ppb K^{-1}, for **12c** Ala^{3}NH Δδ/ΔT = -2.2 ppb K^{-1}). The VT-NMR analyses of CPPs **14** and **15** were consistent with parent peptide **3a**, supported again the presence of strong hydrogen bonds on Val^{4}NH and isoAsp^{5}NH. For peptide **16**, the VT-NMR results indicated the similar hydrogen bonding network on Asp^{3}NH as for the parent **3c**, albeit with a much weaker hydrogen bond (Δδ/ΔT = -2.6 ppb K^{-1}).

Table S2. Δδ/Δt values (ppb K^{-1}) of amide protons for LDV CPPs (CPPs) **3a-d**, and of the correlated **11a, 11c, 12a, 12c, 13-16** by VT NMR spectroscopy, determined at 400 MHz (**3a-d**, **11a, 11c, 12a, 12c, 13**) or at 600 MHz (**14-16**) in DMSO-d_6/H_2O (8:2) over the range 298-318 K.

CPP	Sequence	Phu^{1}NH	AA^{2}NH	AA^{3}NH	AA^{4}NH	isoAsp^{5}NH
**3a**	*c*[(*S*)-Phu-LDV-(*S*)-isoAsp]	-5.0	-7.0	-4.0	-0.8	-0.5
**3b**	*c*[(*S*)-Phu-LDV-(*R*)-isoAsp]	-4.9	-3.5	-3.7	-1.9	-2.2
**3c**	*c*[(*R*)-Phu-LDV-(*S*)-isoAsp]	-5.5	-5.3	-0.3	-7.9	-5.5
**3d**	*c*[(*R*)-Phu-LDV-(*R*)-isoAsp]	-2.1	-2.9	+0.4	-3.0	-3.5
**11a**	*c*[(*S*)-Phu-LDV-(*R*)-βAla^5]	-4.5	-4.1	-2.2	-0.6	-1.1
**12a**	*c*[(*S*)-Phu-LAV-(*S*)-isoAsp^5]	-5.4	+1.6	-4.4	-0.5	0.0
**13**	*c*[(*S*)-Phu-LDV-(*S*)-isoAsp(NHPr)]	-1.7	-4.8	-4.9	-0.9	-0.2
**11c**	*c*[(*R*)-Phu-LDV-(*R*)-βAla]	-6.0	-3.9	-0.7	-6.4	-2.5
**12c**	*c*[(*R*)-Phu-LAV-(*S*)-isoAsp]	-3.7	-4.2	-2.2	-6.0	-6.0
**14**	*c*[(*S*)-Phu-FDV-(*S*)-isoAsp]	-3.5	-4.8	-3.7	-0.8	0.0
**15**	*c*[(*S*)-Phu-FAV-(*S*)-isoAsp]	-4.2	-5.8	-3.8	-0.6	+0.6
**16**	*c*[(*R*)-Phu-LD-Phg-(*S*)-isoAsp]	-5.7	-3.9	-2.6	-4.6	-4.6

Table S3. ROESY cross peaks for **3a** in 8:2 DMSOd$_6$/H_2O; vs = very strong, s = strong, m = medium, w = weak.

Crosspeak	Intensity	Crosspeak	Intensity
PhuNHb-PhuMe	w	PhuArH2',6'-LeuHγ	w
PhuNHb-LeuHα	w	PhuArH2',6'-PhuMe	w
LeuNH-LeuMe	m	PhuArH2',6'-isoAspHβ$_{2.6}$	w
LeuNH-LeuHγ	m	PhuArH2',6'-PhuHβ$_{2.7}$	w
LeuNH-LeuHβ$_{1.5}$	w	PhuArH2',6'-PhuHβ$_{2.9}$	w
LeuNH-LeuHβ$_{1.7}$	m	PhuArH2',6'-PhuHα	w
LeuNH-PhuHβ$_{2.9}$	m	PhuArH3',5'-3,5-LeuMe	m
LeuNH-LeuHα	vs	PhuArH3',5'-3,5-LeuHγ	w
LeuNH-PhuHα	s	PhuArH3',5'-3,5-LeuHβ	w
LeuNH-PhuArH3',5'	m	PhuArH3',5'-3,5-PhuMe	w
LeuNH-AspNH	s	PhuArH3',5'-3,5-PhuHα	vs
AspNH-LeuMe	w	PhuArH3',5'-3,5-isoAspHβ$_{2.6}$	w
AspNH-LeuHγ	w	isoAspNH-ValMe	m
AspNH-LeuHβ$_{1.5}$	s	isoAspNH-PhuMe	w
AspNH-LeuHβ$_{1.7}$	s	isoAspNH-ValHβ	w
AspNH-AspHβ$_{2.9}$	w	isoAspNH-isoAspHβ$_{2.6}$	m
AspNH-AspHβ$_{3.0}$	w	isoAspNH-isoAspHβ$_{2.7}$	w
AspNH-LeuHα	s	isoAspNH-ValHα	s
AspNH-ValHα	w	isoAspNH-AspHα	w

AspNH-AspHα	vs	isoAspNH-isoAspHα	s
AspNH-ValNH	s	isoAspHα-ValMe	w
PhuNH-isoAspHβ$_{2.6}$	s	isoAspHα-isoAspHβ$_{2.6}$	m
PhuNH-isoAspHβ$_{2.7}$	s	isoAspHα-isoAspHβ$_{2.7}$	w
PhuNH-PhuHβ$_{2.7}$	vs	PhuHα-PhuHβ$_{2.7}$	m
PhuNH-PhuHβ$_{2.9}$	m	PhuHα-PhuHβ$_{2.9}$	s
PhuNH-PhuHα	m	AspHα-AspHβ$_{2.9}$	m
PhuNH-isoAspHα	m	AspHα-AspHβ$_{3.0}$	m
PhuNH-isoAspNH	m	ValHα-ValMe	vs
PhuNH-PhuArH3',5'	s	ValHα-ValHβ	s
PhuNHa-LeuMe	w	ValHα-AspHβ$_{2.9}$	w
PhuNHa-LeuHβ$_{1.7}$	w	LeuHα-LeuMe	vs
PhuNHa-LeuHα	w	LeuHα-LeuHγ	m
PhuNHa-PhuHα	w	LeuHα-LeuHβ$_{1.5}$	s
PhuNHa-PhuMe	vs	LeuHα-LeuHβ$_{1.7}$	m
PhuNHa-PhuNHb	vs	PhuHβ$_{3.0}$-LeuHγ	m
ValNH-ValMe	vs	PhuHβ$_{3.0}$-LeuHβ$_{1.5}$	w
ValNH-LeuHβ$_{1.7}$	w	AspHβ$_{2.8}$-LeuHβ$_{1.5}$	w
ValNH-ValHβ	m	AspHβ$_{2.9}$-LeuMe	m
ValNH-AspHβ$_{2.9}$	m	PhuHβ$_{2.7}$-LeuHγ	w
ValNH-ValHα	s	PhuMe-LeuMe	m
ValNH-AspHα	m	PhuMe-LeuHγ	w
ValNH-isoAspHα	w	PhuMe-LeuHβ$_{1.5}$	w
ValNH-isoAspNH	s	PhuMe-LeuHβ$_{1.7}$	w
PhuArH2',6'-LeuMe	m		

Table S4. ROESY cross peaks for **3b** in 8:2 DMSOd$_6$/H$_2$O; vs = very strong, s = strong, m = medium, w = weak.

Crosspeak	Intensity	Crosspeak	Intensity
NHb-LeuMe	w	isoAspNH-isoAspHα	m/s
NHb-PhuHα	w	PhuNHa-ValMe	w
NHb-PhuMe	w	PhuNHa-PhuMe	s/vs
AspNH-Val/LeuMe	w	PhuArH6-LeuMe	w
AspNH-LeuHγ/LeuHβ$_{1.5}$	s	PhuArH2',6'-LeuMe	w/m
AspNH-LeuHβ$_{1.7}$	m	PhuArH2',6'-LeuHγ/LeuHβ$_{1.7}$	w
AspNH-AspHβx2	m	PhuArH2',6'-LeuHβ$_{1.5}$	vw
AspNH-LeuHα	w	PhuArH2'6'-PhuHβ$_{2.7}$	w
AspNH-AspHα	vs	PhuArH2',6'-Phuβ$_{3.0}$	vw
AspNH-ValNH	w	PhuArH2',6'-Phuα	vw
AspNH-LeuNH	w	PhuArH3',5',3,5-LeuMe	m/w
LeuNH-LeuMe	m	PhuArH3',5',3,5-LeuHγ/LeuHβ$_{1.7}$	w
LeuNH-LeuHγ/LeuHβ$_{1.5}$	m/s	PhuArH3',5',3,5-LeuHβ$_{1.5}$	w

LeuNH-LeuHβ$_{1.7}$	m	PhuArH3',5',3,5-PhuHβ$_{2.7}$	s
LeuNH-PhuHβ$_{2.7}$	w	PhuArH3'5',3,5-PhuHβ$_{3.0}$	s
LeuNH-PhuHβ$_{3.0}$	w/m	PhuArH3'5',3,5-PhuHα	s
LeuNH-LeuHα	s	AspHα-LeuHγ/LeuHβ$_{1.7}$	w
LeuNH-PhuHα	m	AspHα-AspHβx2	vs
PhuNH-isoAspHβ$_{2.2}$	vw	AspHα-ValHα	w
PhuNH-PhuHβ$_{2.7}$	s/vs	PhuHα-PhuHβ$_{2.7}$	m
PhuNH-isoAspHβ$_{2.8}$	s/vs	PhuHα-PhuHβ$_{3.0}$	vs
PhuNH-PhuHβ$_{3.0}$	w	isoAspHα-isoAspHβ$_{2.4}$	vs
PhuNH-PhuHα	m	isoAspHα-isoAspHβ$_{2.8}$	w
PhuNH-PhuAr3',5'	m/s	isoAspHα-ValHα	w
ValNH-ValMe	s	LeuHα-LeuMe	vs
ValNH-LeuHγ/LeuHβ	w,w	LeuHα-LeuHβ$_{1.7}$/Hγ	s/vs
ValNH-ValHβ	m	LeuHα-LeuHβ$_{1.5}$	w/m
ValNH-AspHβ	m	ValHα-LeuMe	vs
ValNH-ValHα	m	ValHα-ValHβ	vs
ValNH-AspHα	m/w	PhuHβ$_{3.0}$-LeuHβ$_{1.7}$/Hγ	w
isoAspNH-ValMe	w	PhuHβ$_{3.0}$-LeuMe	w
isoAspNH-ValHβ	m/s	PhuHβ$_{2.7}$-LeuMe	w
isoAspNH-isoAspHβ$_{2.8}$	m/s	AspHβx2-LeuHβ$_{1.7}$/Hγ	w
isoAspNH-ValHα	w/m	AspHβx2-LeuHβ$_{1.5}$	w

Table S5. ROESY cross peaks for **3c** in 8:2 DMSOd$_6$/H_2O; vs = very strong, s = strong, m = medium, w = weak.

Crosspeak	Intensity	Crosspeak	Intensity
PhuNHb-LeuMe$_{0.6}$	w	PhuNHa-LeuMe$_{0.7}$	w
PhuNHb-LeuMe$_{0.7}$	m	PhuNHa-Phu(2-Me)	vs
PhuNHb-ValMe$_{0.8}$	w	PhuNHa-ValHα	w
PhuNHb-Phu(2-Me)	w	PhuArH6-LeuMe$_{0.6}$	w
PhuNHb-LeuNH	w	PhuArH6-LeuMe$_{0.7}$	w
PhuNHb-PhuNH	w	PhuArH6-Phu(2-Me)	w
PhuNHb-ValNH	w	AspNH-LeuHβ	m
PhuNH-ValMe$_{0.8}$	w	AspNH-AspHβ$_{2.6}$	m
PhuNH-isoAspHβ	vs	AspNH-AspHβ$_{2.8}$	m
PhuNH-PhuHβ	vs	AspNH-LeuHα	s
PhuNH-PhuHα	s	AspNH-PhuHα	w
PhuNH-isoAspHα	w	AspNH-AspHα	s
PhuNH-PhuArH3’,5’	m	PhuArH2’,6’-LeuMe$_{0.6}$	m
PhuNH-AspNH	w	PhuArH2’,6’-LeuMe$_{0.7}$	w
PhuNH-ValNH	w	PhuArH2’,6’-ValMe$_{0.8}$	w
isoAspNH-ValMe$_{0.8}$	w	PhuArH2’,6’-LeuHγ	m
isoAspNH-ValHβ	m	PhuArH2’,6’-PhuHα	w

isoAspNH-isoAspHβ	w	PhuArH3’,5’-LeuMe$_{0.6}$	m
isoAspNH-ValHα	s	PhuArH3’,5’-LeuMe$_{0.7}$	w
isoAspNH-isoAspHα	m	PhuArH3’,5’-ValMe$_{0.8}$	w
isoAspNH-AspHα	w	PhuArH3’,5’-LeuHγ	m
isoAspNH-LeuHα	w	PhuArH3’,5’-LeuHβ	w
LeuNH-LeuMe$_{0.6}$	w	PhuArH3’,5’-ValHβ	w
LeuNH-LeuMe$_{0.7}$	w	PhuArH3’,5’-isoAspHβ	w
LeuNH-LeuHγ	s	PhuArH3’,5’-LeuHα	w
LeuNH-LeuHβ	vs	PhuArH3’,5’-isoAspHα	w
LeuNH-AspHβ$_{2.6}$	m	PhuArH3’,5’-PhuHα	s
LeuNH-PhuHβ	m	AspHα-ValMe$_{0.7}$	w
LeuNH-ValHα	w	AspHα-AspHβ$_{2.6}$	s
LeuNH-LeuHα	s	AspHα-AspHβ$_{2.8}$	s
LeuNH-isoAspHα	w	AspHα-ValHα	w
LeuNH-PhuHα	vs	AspHα-LeuHα	w
LeuNH-PhuArH3’,5’	w	PhuHα-PhuHβ	vs
LeuNH-AspNH	s	isoAspHα-isoAspHβ	m
ValNH-ValMe$_{0.8}$	m	LeuHα-LeuMe$_{0.6}$	vs
ValNH-ValMe$_{0.9}$	w	LeuHα-LeuMe$_{0.7}$	m
ValNH-ValHβ	m	LeuHα-LeuHγ	m
ValNH-AspHβ$_{2.6}$	w	LeuHα-LeuHβ	vs
ValNH-AspHβ$_{2.8}$	m	ValHα-ValMe$_{0.7}$	s
ValNH-ValHα	vs	ValHα-ValMe$_{0.8}$	s
ValNH-AspHα	s	ValHα-ValHβ	m
ValNH-AspNH	w	PhuHβ-LeuHγ	w

Table S6. ROESY cross peaks for **3d** in 8:2 DMSOd_6/H_2O; vs = very strong, s = strong, m = medium, w = weak.

Crosspeak	Intensity	Crosspeak	Intensity
PhuNHb-LeuMe$_{0.6}$	w	PhuNHa-PhuMe	vs
PhuNHb-LeuMe$_{0.7}$	w	PhuNHa-PhuArH6	vs
ValNH-ValMe	s	AspNH-LeuMe$_{0.6}$	m
ValNH-ValHβ	w	AspNH-LeuMe$_{0.7}$	m
ValNH-AspHβ$_{2.8}$	w	AspNH-LeuHγ	m
ValNH-ValHα	s	AspNH-AspHβ$_{2.5}$	w
ValNH-AspHα	s	AspNH-AspHβ$_{2.8}$	w
ValNH-AspNH	w	AspNH-LeuHα	w
ValNH-isoAspNH	w	AspNH-AspHα	m
PhuNH-LeuHγ	w	PhuArH2',6'-PhuHα	w
PhuNH-isoAspHβ$_{2.7}$	s	PhuArH3',5'-LeuMe$_{0.6}$	w
PhuNH-PhuHβ$_{2.8}$	s	PhuArH3',5'-LeuHγ	w
PhuNH-PhuHα	m	PhuArH3',5'-LeuHα	w

PhuNH-PhuArH3',5'	w	PhuArH3',5'-PhuHα	s
PhuNH-PhuNHa	w	AspHα-LeuHγ	w
PhuNH-LeuNH	m	AspHα-AspHβ$_{2.5}$	s
PhuNH-isoAspNH	m/s	AspHα-AspHβ$_{2.8}$	m
isoAspNH-ValHβ	w	AspHα-LeuHα	w
isoAspNH-isoAspHβ$_{2.6}$	m	isoAspHα-isoAspHβ$_{2.6}$	m
isoAspNH-ValHα	w	isoAspHα-isoAspHβ$_{2.7}$	m
isoAspNH-isoAspHα	m	PhuHα-PhuArH3',5'	m
isoAspNH-ValMe	w	PhuHα-LeuNH	vs
isoAspNH-AspNH	w	LeuHα-LeuMe$_{0.6}$	s
isoAspNH-LeuNH	w	LeuHα-LeuMe$_{0.7}$	w
LeuNH-LeuMe$_{0.6}$	w	LeuHα-LeuHγ	w
LeuNH-LeuMe$_{0.7}$	w	LeuHα-LeuHβ$_{1.4}$	m
LeuNH-LeuHγ	m	LeuHα-ValHα	w
LeuNH-LeuHβ$_{1.3}$	m	ValHα-ValMe	vs
LeuNH-LeuHα	m	LeuHβ$_{1.4}$-LeuMe$_{0.6}$	w
LeuNH-PhuHα	vs	LeuHβ$_{1.4}$-LeuMe$_{0.7}$	w
LeuNH-AspNH	s	LeuHβ$_{1.3}$-LeuMe$_{0.6}$	w
PhuNHa-PhuNHb	vs		

Table S7. ROESY cross peaks for **12a** in 8:2 DMSOd$_6$/H_2O; vs = very strong, s = strong, m = medium, w = weak.

Crosspeak	Intensity	Crosspeak	Intensity
LeuNH-LeuMe$_{0.5}$	m	PhuArH3',5'- LeuHβ$_{1.8}$	w
LeuNH-LeuHα	s	PhuArH3',5'-PhuHβ$_{2.6}$	vs
LeuNH-PheHα	vs	PhuArH3',5'- PhuHβ$_{2.8}$	s
PhuNHb-LeuMe$_{0.7}$	w	PhuArH3',5'- PhuHα	vs
PhuNH-ValMe$_{1.1}$	w	isoAspNH-isoAspHα	s
PhuNH-isoAspHβ$_{2.6}$	m	isoAspNH-ValHα	m
PhuNH-PheHβ$_{2.8}$	s	ValHα-ValMe$_{0.9}$	s
PhuNH-isoAspHβ$_{2.9}$	s	ValHα-ValMe$_{1.1}$	m
PhuNH-PhuHα	m	ValHα-ValHβ	vs
AlaNH-LeuHβ$_{1.2}$	s	isoAspHα-isoAspHβ$_{2.6}$	m
AlaNH-AlaMe	s	isoAspHα-isoAspHβ$_{2.9}$	vs
AlaNH-LeuHβ$_{1.8}$	s	PheHα-PheHβ$_{2.6}$	m
AlaNH-AlaHα	m	PheHα-PheHβ$_{2.8}$	s
AnaNH-ValNH	m	LeuHα-LeuMe$_{0.5}$	vs
PhuNHa-Phu(2-Me)	vs	LeuHα-LeuMe$_{0.7}$	m
ValNH-ValMe$_{0.9}$	m	LeuHα-LeuHβ$_{1.2}$	vs
ValNH-ValMe$_{1.1}$	s	PhuHβ$_{2.8}$-LeuHβ$_{1.2}$	w
ValNH-AlaHα	m	LeuHβ$_{1.8}$-LeuMe$_{0.5}$	s
ValNH-ValHα	m	LeuHβ$_{1.8}$-LeuMe$_{0.7}$	s

ValNH-isoAspNH	vs	LeuH$\beta_{1.8}$-LeuHγ	w
PhuArH2’,6’-LeuMe$_{0.5}$	m	AlaMe-LeuMe$_{0.5}$	w
PhuArH2’,6’-LeuMe$_{0.7}$	s	AlaMe-LeuMe$_{0.7}$	w
PhuArH3’,5’-LeuMe$_{0.5}$	m	LeuH$\beta_{1.2}$-LeuMe$_{0.5}$	m
PhuArH3’,5’-LeuMe$_{0.7}$	w	LeuH$\beta_{1.2}$-LeuMe$_{0.7}$	s
PhuArH3’,5’-LeuH$\beta_{1.2}$	w	LeuH$\beta_{1.2}$-LeuHγ	m

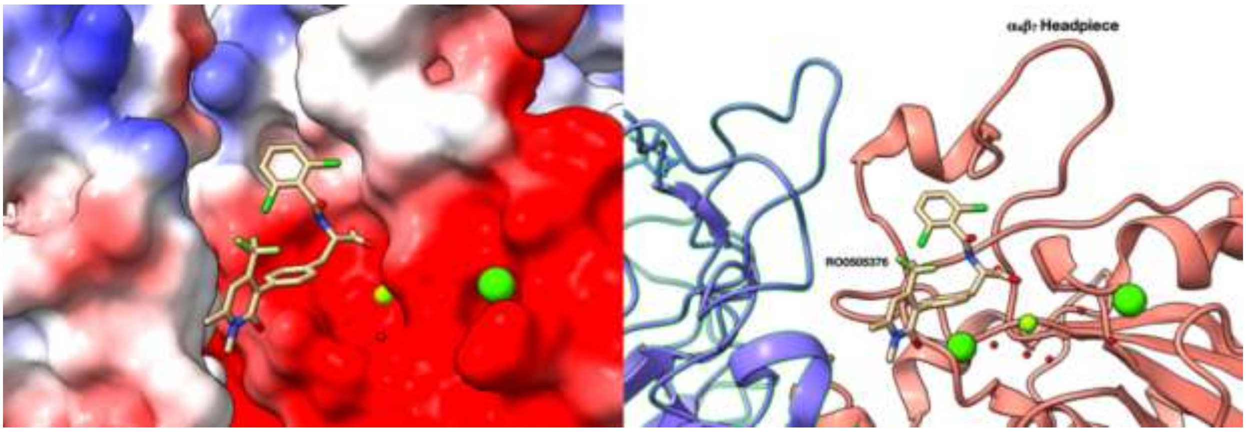

Figure S14. PDB 3V4V, crystal structure of $\alpha_4\beta_7$ headpiece complexed with RO0505376; left, the integrin is represented as molecular solvent-excluded surfaces (SES) and colored using Coulombic electrostatic potential (ESP). RO0505376 is rendered in sticks, while metal ions were rendered as green spheres.

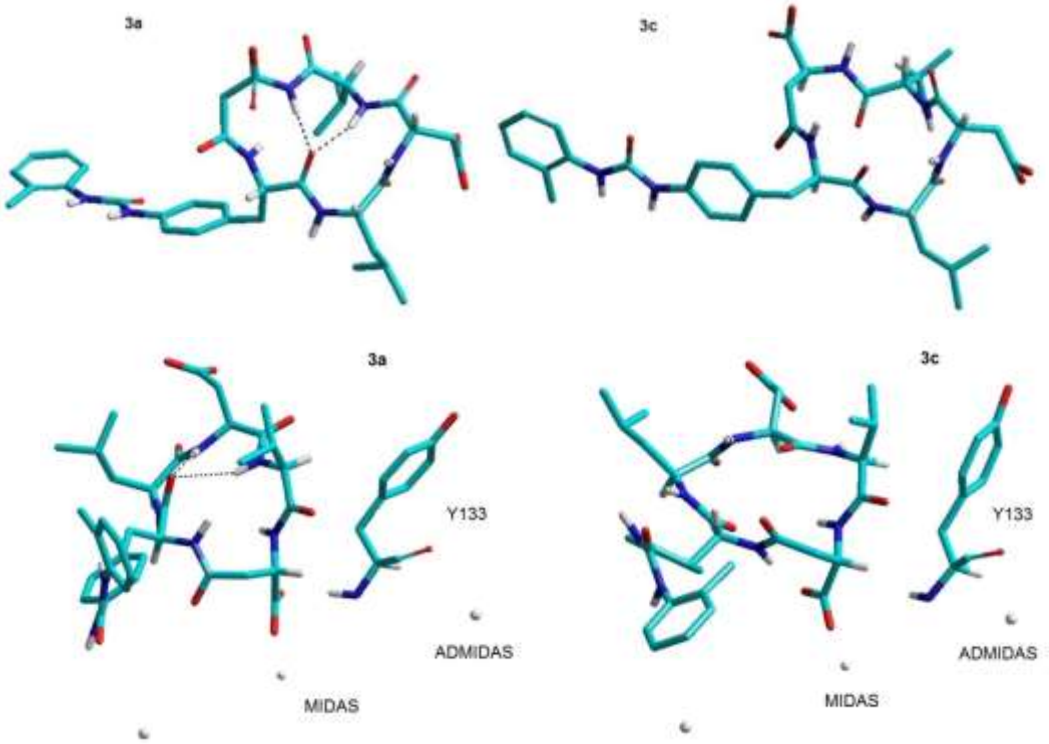

Figure S15. In-solution (top) and receptor-bound (bottom) structures of **3a**, **3c** showing hydrogen-bonded secondary structure elements (dotted lines). The structures differ only by the inversion of stereochemistry at Phu[1]. β_1:Tyr^{133}and cations of the adhesion sites are shown for better comparison.

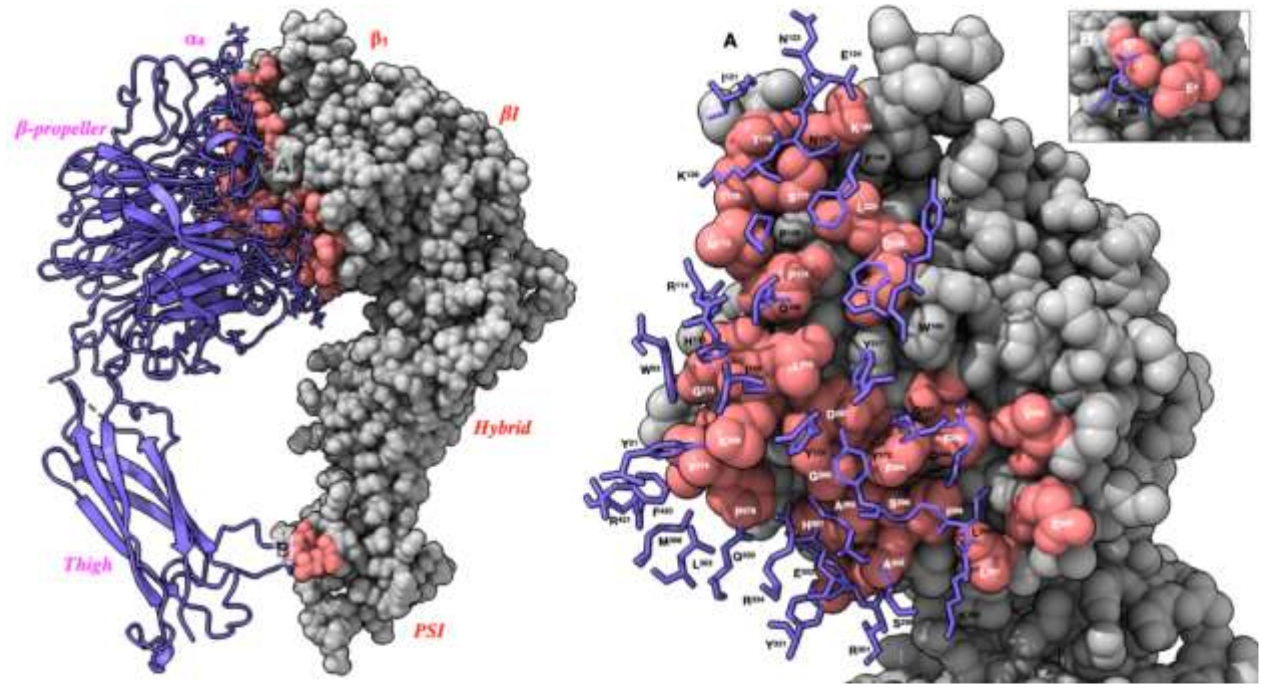

Figure S16. Left. Rendering of $\alpha_4\beta_1$ integrin model used in this work, with evidenced the β-propeller (α_4), Thigh (α_4), βI (β_1), Hybrid (β_1) and PSI (plexin–semaphorin–integrin, β_1) domains. The α_4 subunit is colored in blue and represented as solid ribbon, with key residues at the α_4/β_1 interface in stick. The β_1 subunit is represented as gray CPK, with key residues at the α_4/β_1 interface in pink. (A) Close look of the interface between the β-propeller (α_4) and βI (β_1) domains. The α_4 residues are shown in stick representation and colored in blue (black labels), while the β_1 residues are represented in CPK, with key residues colored in pink (white labels). (B) Particular of the interface between Thigh (α_4)

and PSI (β_1) domains. Again, the α_4 residues are represented as blue sticks (black labels), while the β_1 residues are represented in CPK, with key residues colored in pink (white labels). Molecular graphics and analyses performed with UCSF ChimeraX.

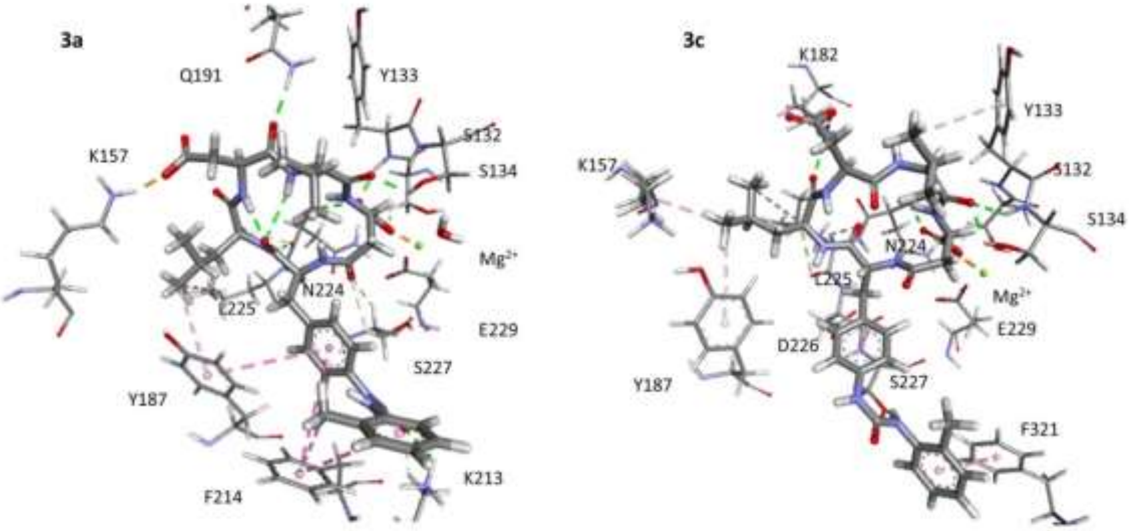

Figure S17. The agonist **3a**, c[(*S*)-Phu-LDV-(*S*)-isoAsp], makes many interactions with α_4 subunit. Phu^1MePh group interacts with α_4:F^{214} (π-π stacking), Phu^1Ph interacts with α_4:F^{214} (π-π T-shaped interaction), and with α_4:Y^{187} (π-π stacking). UreaC=O makes a hydrogen bond with α_4:K^{213} (2.31 Å). Phu^1C=O establishes intramolecular hydrogen bonds with Asp^3NH (1.72 Å) and Val^4NH (2.30 Å). The hydrophobic side chain of Leu^2 is included within a cavity delimited by α_4:K^{157}, β_1:L^{225}, and α_4:Y^{187}, while Leu^2NH group makes a hydrogen bond with β_1:$N^{224}O$ (1.84 Å). Asp^3COO^- makes a salt bridge with α_4:$K^{157}N\zeta^+$ (1.63 Å), while Asp^3C=O gives rise to an hydrogen bond with β_1:$Q^{191}CONH_2$ (1.78 Å). Val^4C=O is hydrogen bonded to β_1:$S^{134}OH$ (1.97 Å). The isopropyl of Val^4 is pseudo axial, therefore it does get in touch with any residues of β_1 subunit. Finally, $isoAsp^5COO^-$ is coordinated to Mg^{2+} in MIDAS, with Mg^{2+} that exhibits a slightly distorted coordination geometry (RMSD 0.12). Two hydrogen bonds are formed with water (W) W1 (2.86 Å) and W3 (2.67 Å), an hydrogen bond with β_1:$Y^{133}NH$ (1.97 Å) and an hydrogen bond with β_1:$N^{224}NH$ (2.75 Å).

The antagonist **3c**, c[(*R*)-Phu-LDV-(*S*)-isoAsp], differs from the all-S configured **3a** for the inverted stereochemistry at Phu^1. The most noticeable effect is represented by a greater interaction with β_1 subunit. Phu^1MePh makes a π-π stacking interaction with β_1:F^{321}. UreaNH makes a hydrogen bond with β_1:$S^{227}OG$ (2.11 Å), and Phu^1Ph interacts with the peptide bond between β_1:D^{226} and β_1:S^{227} (amide-π stacking). The side chain of Leu^2 is included within the cavity formed by α_4:K^{157}, α_4:Y^{187}, β_1:L^{225}. Leu^2C=O makes an hydrogen bond with β_1:$K^{182}NH\zeta^+$ (1.77 Å), which in turn interacts with Asp^3COO^- by a salt bridge (1.62 Å). Val^4C=O makes two hydrogen bonds with β_1:$S^{134}OH$ (1.97 Å) and β_1:$S^{134}NH$ (1.52 Å). In contrast to **3a**, the isopropyl of Val^4 is in contact with β_1:Tyr^{133} (π-alkyl, 4.76 Å). Finally, $isoAsp^5COO^-$ is coordinated with Mg^{2+}. In this complex, the cation show a distorted octahedral coordination geometry (RMSD 0.17); $isoAsp^5COO^-$ makes also other interacts: with β_1:S^{132} (O-HC, 2.46 Å), two hydrogen bonds with W^1 (2.60 Å) and W^3 (2.66 Å), hydrogen bond with β_1:$N^{224}NH$ (1.92 Å).

Molecular graphics and detailed analysis of the interactions between ligands and integrin $\alpha_4\beta_1$ receptor model. The following figures show the best binding conformation of the ligands within $\alpha_4\beta_1$ integrin binding site, represented as molecular solvent-excluded surfaces (partially transparent), colored using Coulombic electrostatic potential (ESP), with default coloring ranging from red for negative potential through white to blue for positive potential. Ligands are rendered in sticks, while metal ions belonging to MIDAS and ADMIDAS are rendered as green colored spheres. Key receptor residues in thick sticks. Molecular graphics and analyses were performed with Biovia Discovery Studio visualizer.

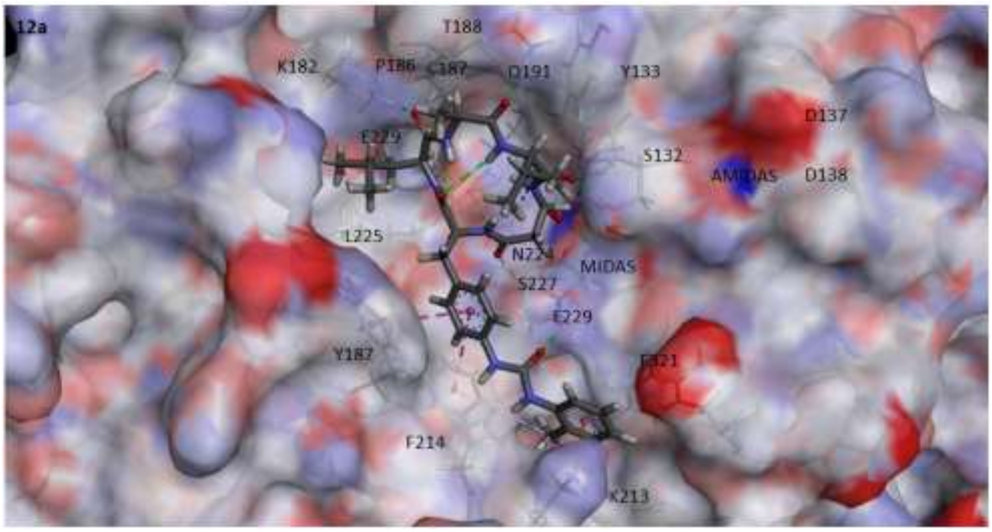

Figure S18. The CPPs **12a**, *c*[(*S*)-Phu-LAV-(*S*)-isoAsp], maintains the same stereochemistry as **3a**, but Asp^3 is replaced by Ala^3. Phu^1MePh group is in contact with β_1:F^{321} through a π-π stacking interaction and α_4:K^{213}NHζ$^+$ through a π-cation interaction (2.12 Å). UreaC=O makes a conventional hydrogen bond with β_1:S^{227}OH (2.21 Å). Phu^1Ph interacts with α_4:F^{214} through π-π T-shaped interaction and with α_4:Y^{187} through a stacked π-π interaction. Phu^1C=O makes two intramolecular hydrogen bonds with Ala^3NH (1.78 Å) and Val^4NH (2.91 Å). Leu^2NH give rise to a hydrogen bond with β_1:N^{224}O (1.76 Å), while Leu^2C=O makes a hydrogen with β_1:K^{182}NHζ$^+$ (2.01 Å). In addition to the intramolecular bond with Phu^1C=O, Ala^3 shows an additional hydrogen bond between Ala^3C=O and β_1:N^{191}Hε (1.89 Å). The methyl of Val^3 nicely packs against methyl of β_1:T^{188}. Val^4C=O makes two hydrogen bonds, with β_1:S^{134}NH (2.95 Å) and β_1:S^{134}OH (1.69 Å). $isoAsp^5$ residue coordinates Mg^{2+} (RMSD 0.11). Furthermore, $isoAsp^5COO^-$ is involved in a hydrogen bonds network with W^1 (2.83 Å), W^3 (2.72 Å), β1:Y^{133}NH (2.45 Å), β_1:N^{224}NH (2.08 Å) and β_1:N^{224}NHδ21 (2.55 Å).

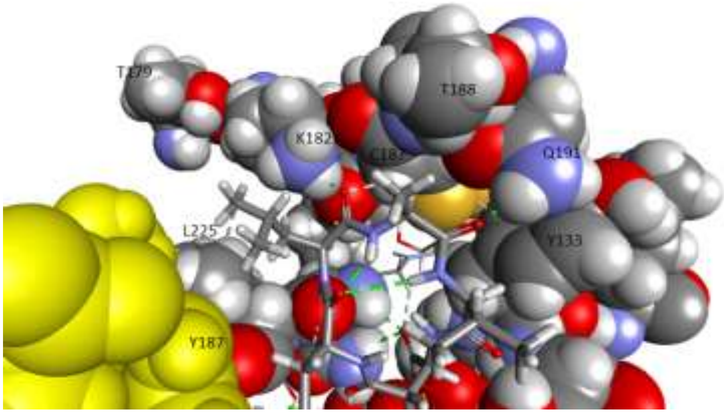

Figure S19. Detail of the upper region of the binding site of $\alpha_4\beta_1$ integrin hosting **12a**, *c*[(*S*)-Phu-LAV-(*S*)-isoAsp]. Receptor residues in contact with the cyclopeptide are rendered as CPK models; residues of α_1 subunit are highlighted in yellow. The sequence A^{181}-K-L-R-N-P-C-T^{188} of the β_1 subunit is replaced by S^{191}-K-L-R-H-P-C-P^{198} in β_7 subunit. In the $\alpha_4\beta_1$-**12a** complex, the side chain of Thr^{188} of β_1 subunit invades the space of Val^3, so that the methyl of the latter nicely packs against the methyl of Thr^{188}. In the β_7 subunits, this residue is mutated for Pro^{198}.

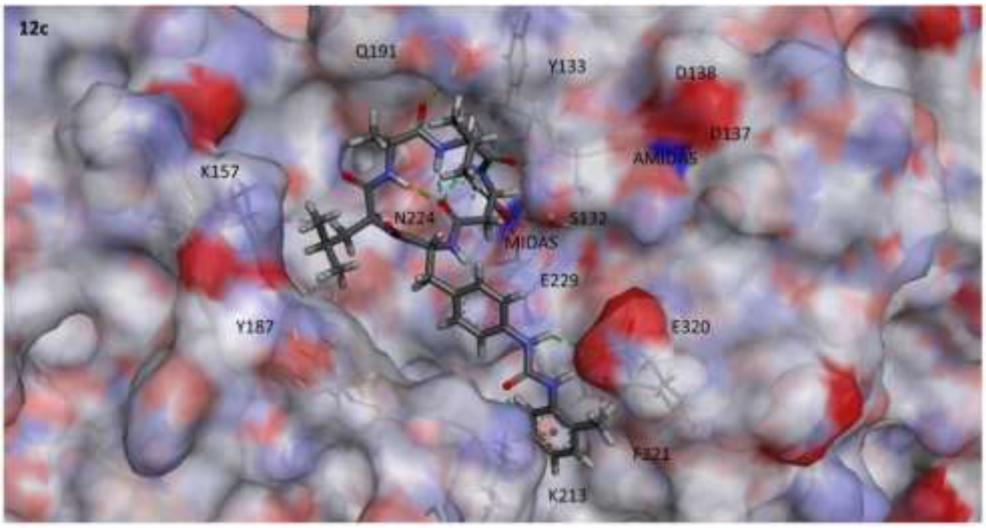

Figure S20. The CPPs **12c**, *c*[(*R*)-Phu-LAV-(*S*)-isoAsp], is an analogue of **3c**, sharing the same stereochemistry array, in which Asp^3 is replaced by Ala^3. This cyclopeptide shows interactions with both α_4 and β_1 subunits. MePh group of $Phu^1$is in contact with α_4:K^{213} (π-cation interaction with NHζ$^+$, 3.76 Å, plus π-alkyl, 3.76 Å) and with β_1:F^{321} (π-alkyl, 4.37 Å). UreaC=O is hydrogen bonded to α_4:K^{213}NHζ$^+$ (1.84 Å), while both ureaNHs are hydrogen bonded to β_1:$Glu^{320}COO^-$ (1.94 Å, 2.90 Å). The side chain of Leu^2 is inserted within the cavity delimited by β_1:L^{225}, α_4:K^{157}, α_4:Y^{187}, making hydrophobic interactions. Ala^3NH is involved in a intramolecular hydrogen bond with isoAspC=O (2.03 Å), Ala^3C=O makes a hydrogen bond with β_1:$N^{191}CONH_2$ (2.19 Å), while Ala^3methyl is close to β_1:T^{188}methyl (alkyl interaction). Val^4NH is involved in a intramolecular hydrogen bond with isoAspC=O (1.81 Å), Val^4C=O is hydrogen bonded to β_1:S^{134}NH (2.79 Å), but Val^4isopropyl is directed perpendicularly above the molecular plane, therefore making very little contacts with any residues of β_1 subunit. Finally, $isoAsp^5COO^-$ is coordinated to Mg^{2+} in MIDAS.

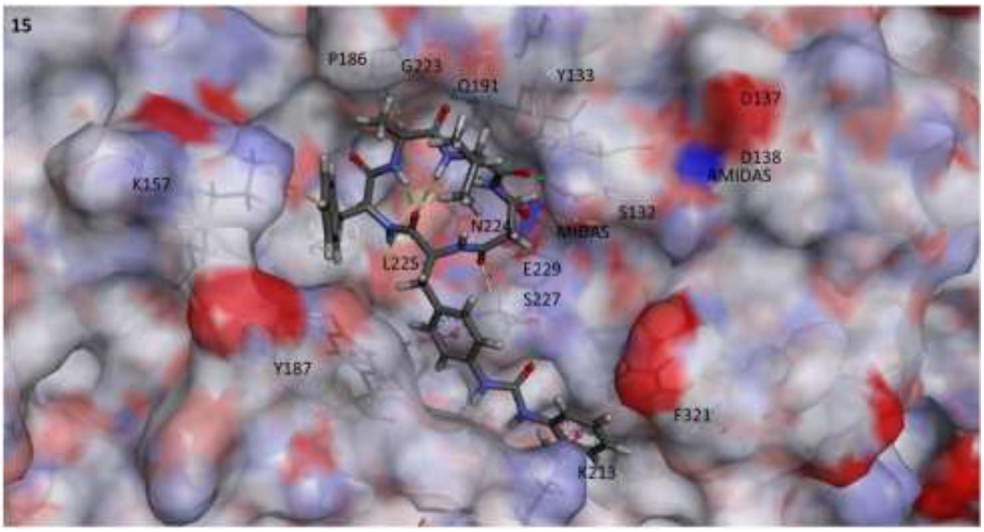

Figure S21. The CPP **15** was obtained from **3a** by replacing Leu^2 with Phe^2 and Asp^3 with Ala^3. The Phu^1MePh group is located between the residues β_1:F^{321} and α_4:K^{213}, with which it interacts giving rise to a π-π stacking interaction and a π-cation interaction (3.19 Å). UreaC=O makes a conventional hydrogen bond with β_1:S^{227}OH (2.05 Å), while Phu^1PH makes a π-π stacking interactions with α_4:Y^{187}. Phu^1C=O makes two intramolecular hydrogen bonds with Ala^3NH (1.72 Å) and Val^4NH (2.15 Å). Phe^2NH makes two hydrogen bonds with β_1:N^{224}O (2.87 Å) and with β_1:L^{225}O (2.14 Å), while Phe^2Ph is hold in position by a π-cation interaction with β_1:K^{182}Nζ$^+$ (4, 01 Å). In addition to the intramolecular hydrogen bonds with Phu^1, Ala^3 residue makes an hydrogen bond between C=O and β_1:Gln^{191}Hε22 (1.89 Å), while the Val^4C=O forms an hydrogen bond with β_1:S^{134}OH (1.85 Å). The carboxyl group of the $isoAsp^5$ residue makes two water-hydrogen bonds with W^1 (2.84 Å) and W^3 (2.70 Å), an hydrogen bond with β1:Y^{133}NH (2.12 Å) and an hydrogen bond with β_1:N^{224}NH (2.30 Å). $isoAsp^5COO^-$ coordinates the magnesium ion in MIDAS (RMSD 0.15).

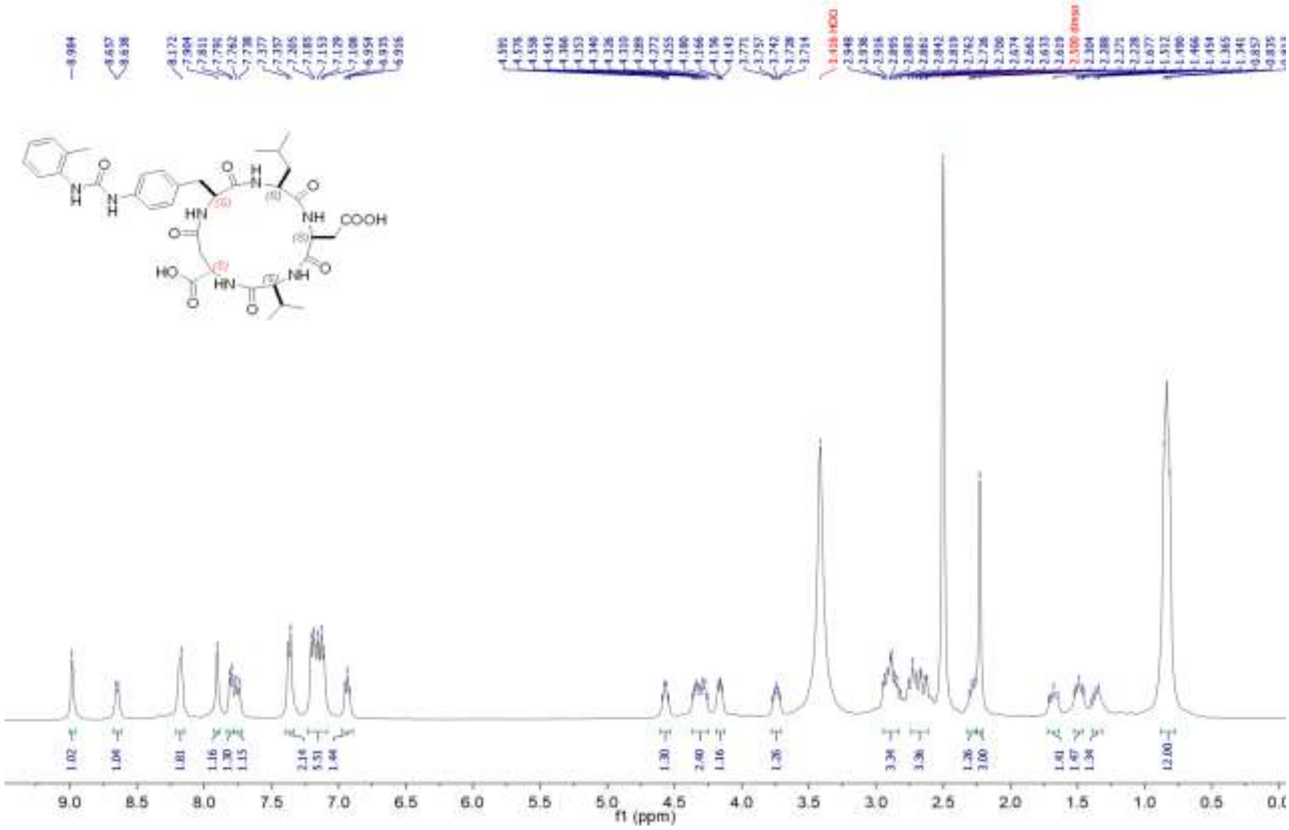

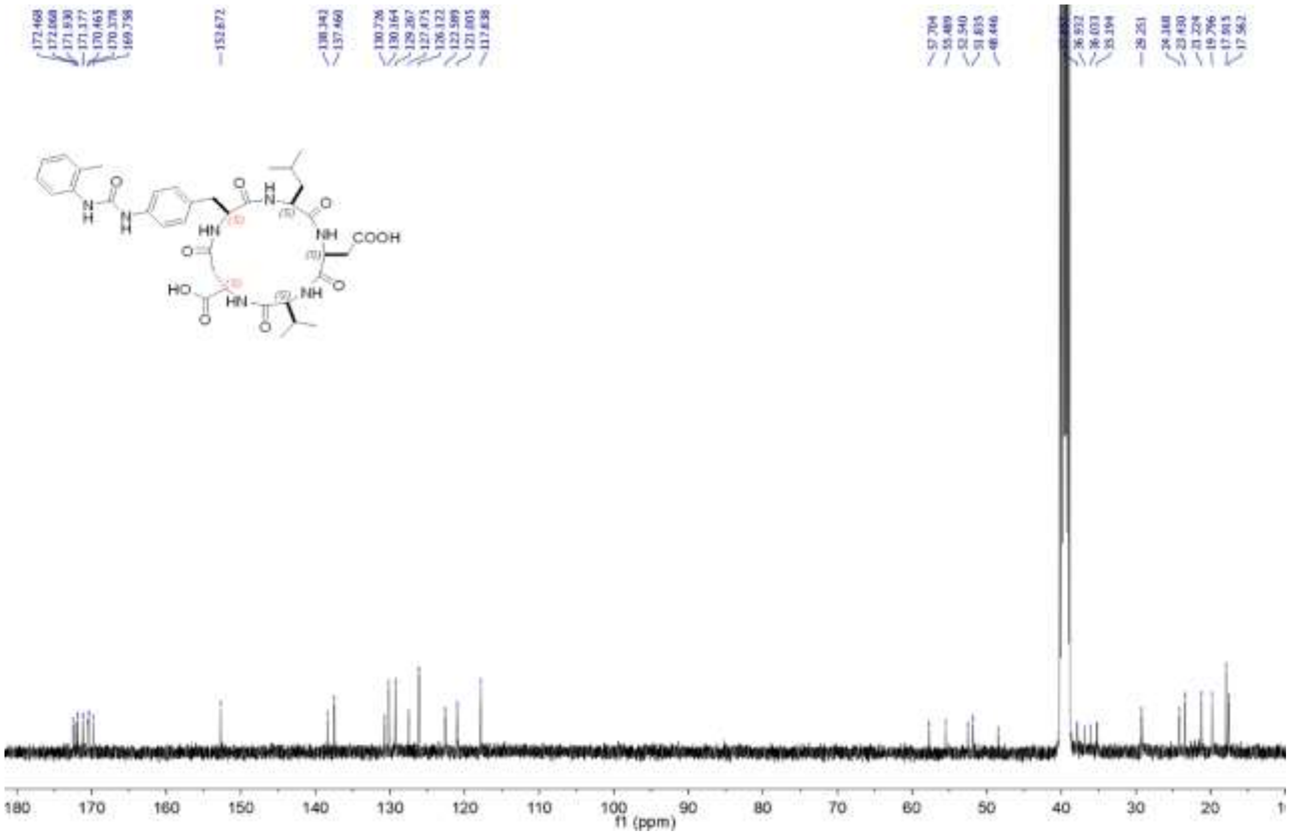

Figure S22. ^{1}H NMR (8:2 $DMSOd_6/H_2O$ at 400 MHz) and ^{13}C NMR ($DMSOd_6$, 100 MHz) of **3a**.

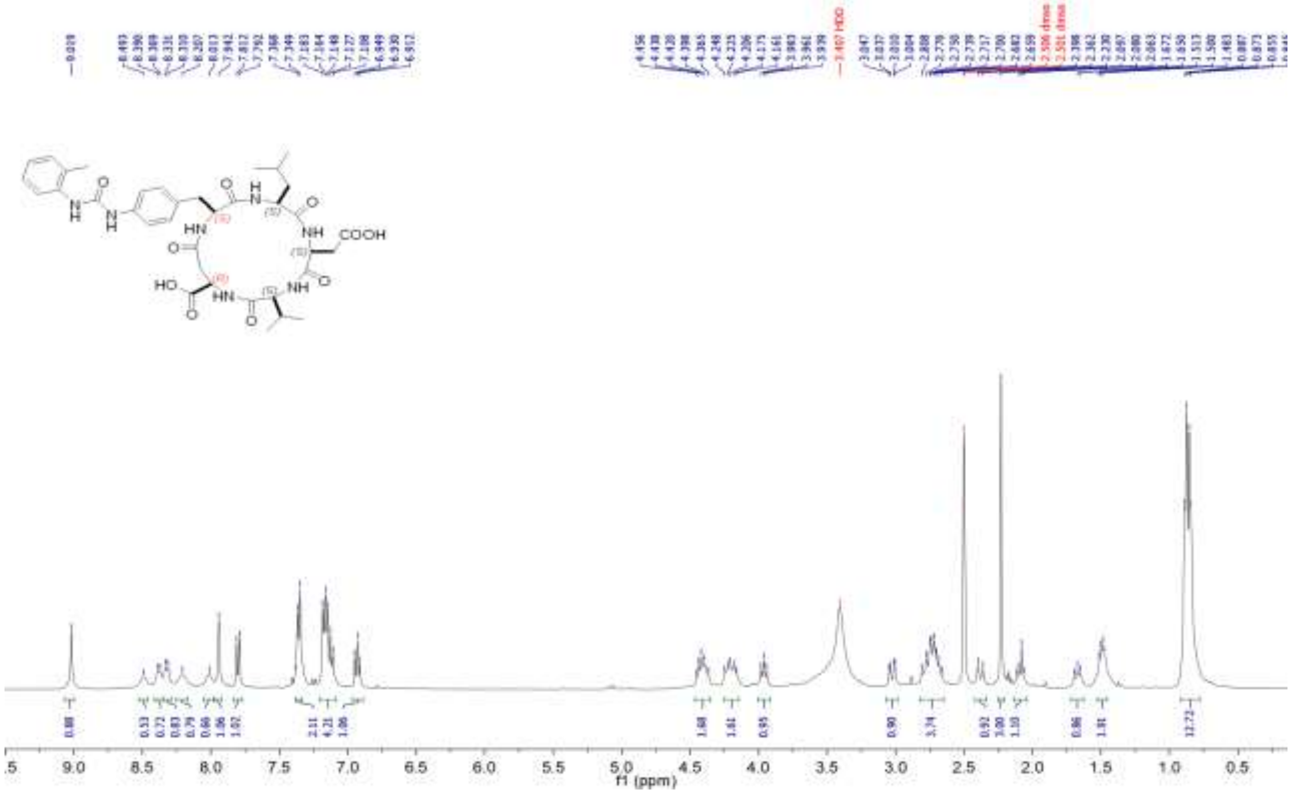

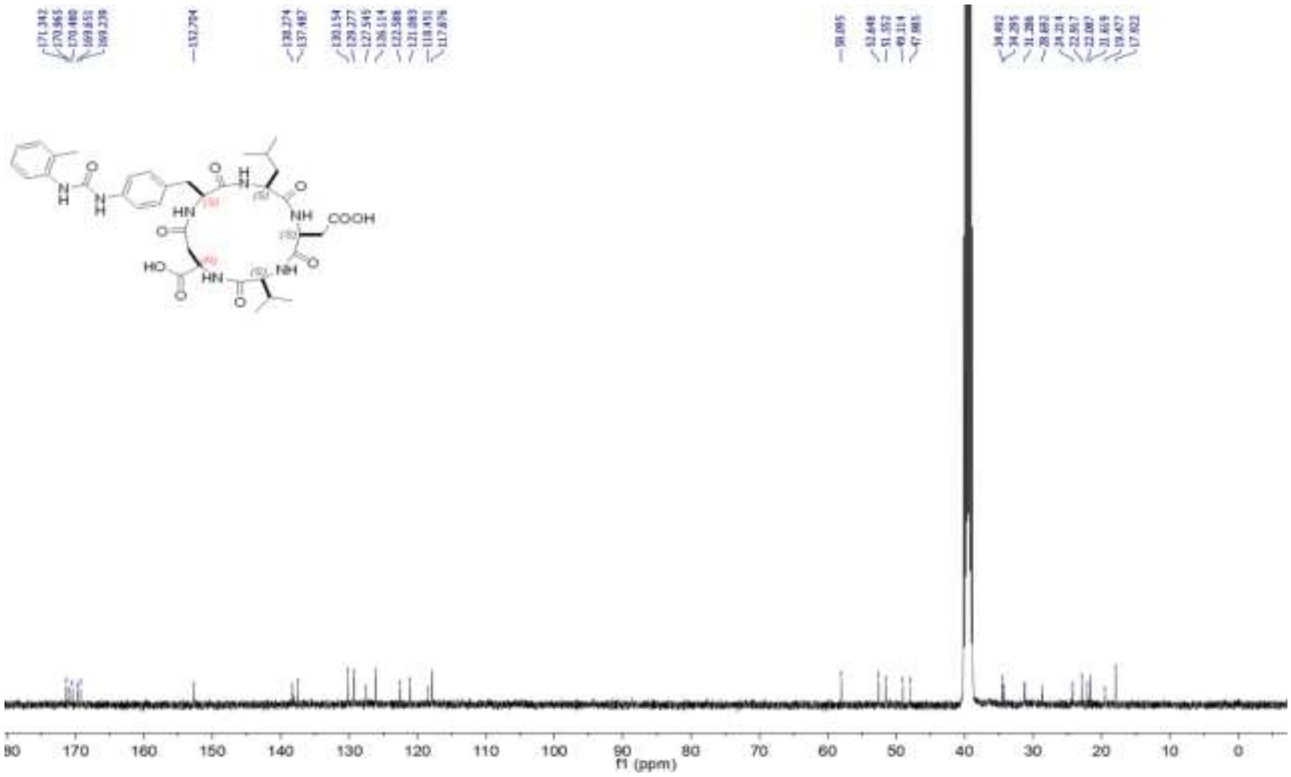

Figure S23. ^{1}H-NMR (8:2 DMSOd$_6$/H_2O at 400 MHz) and ^{13}C-NMR (DMSOd$_6$, 100 MHz) of **3b**.

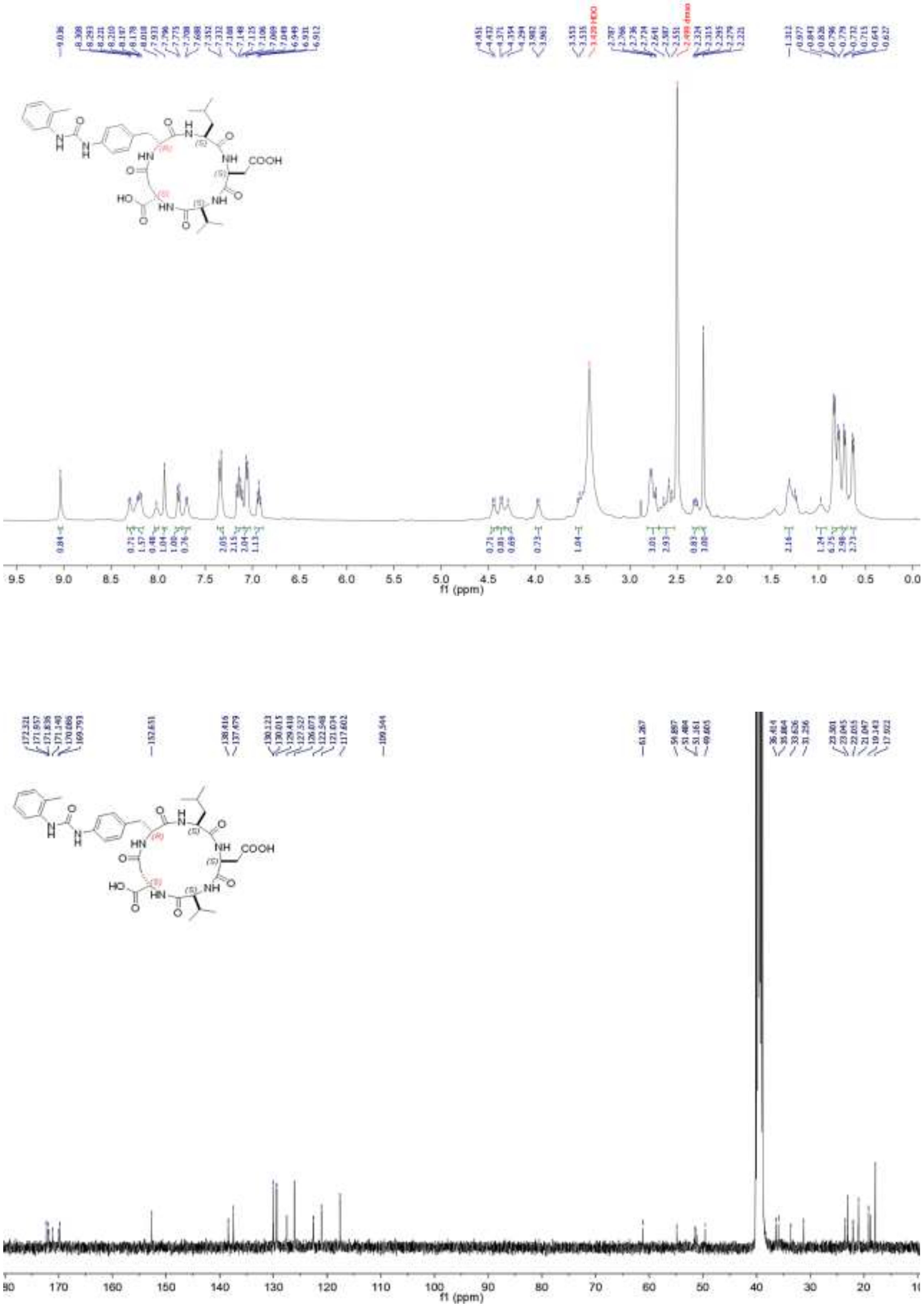

Figure S24. ^{1}H-NMR (8:2 DMSOd$_6$/H_2O at 400 MHz) and ^{13}C-NMR (DMSOd$_6$, 100 MHz) of **3c**.

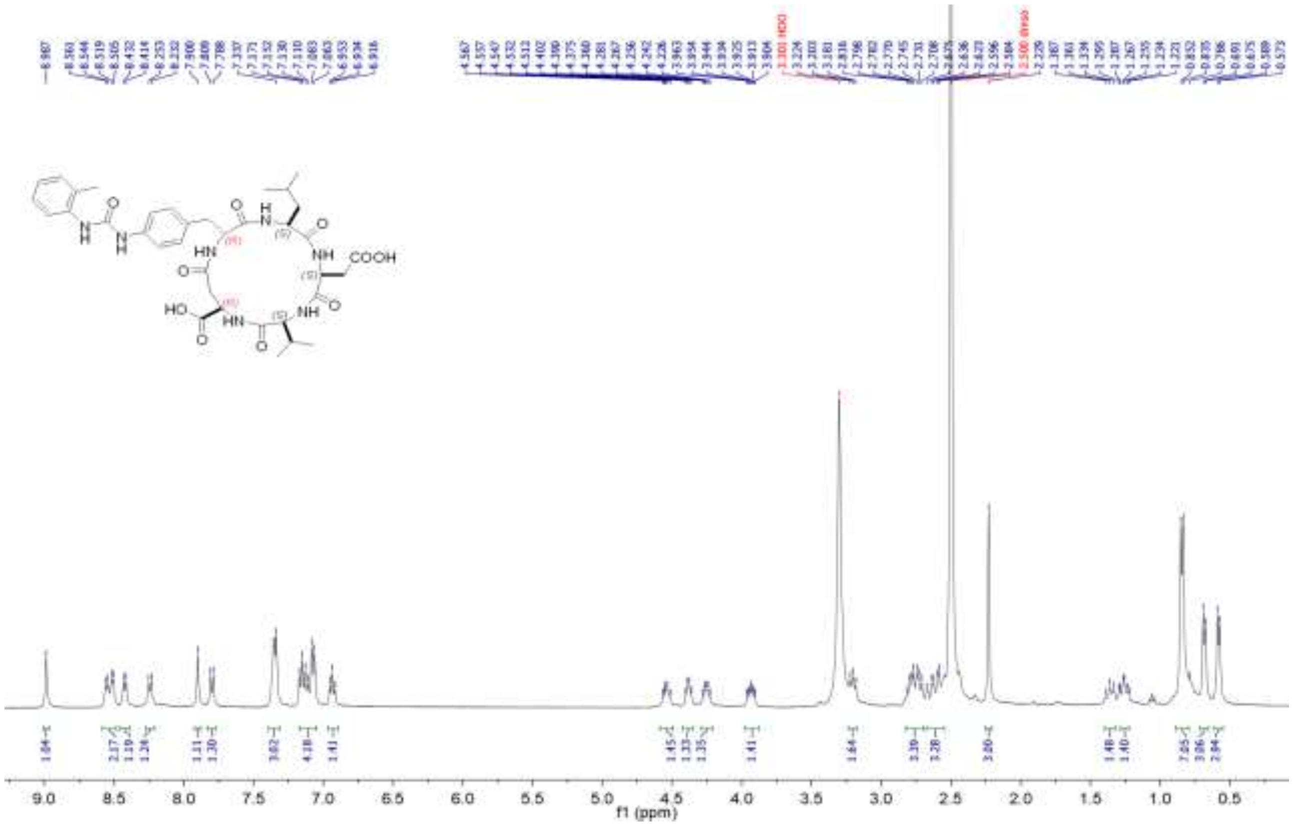

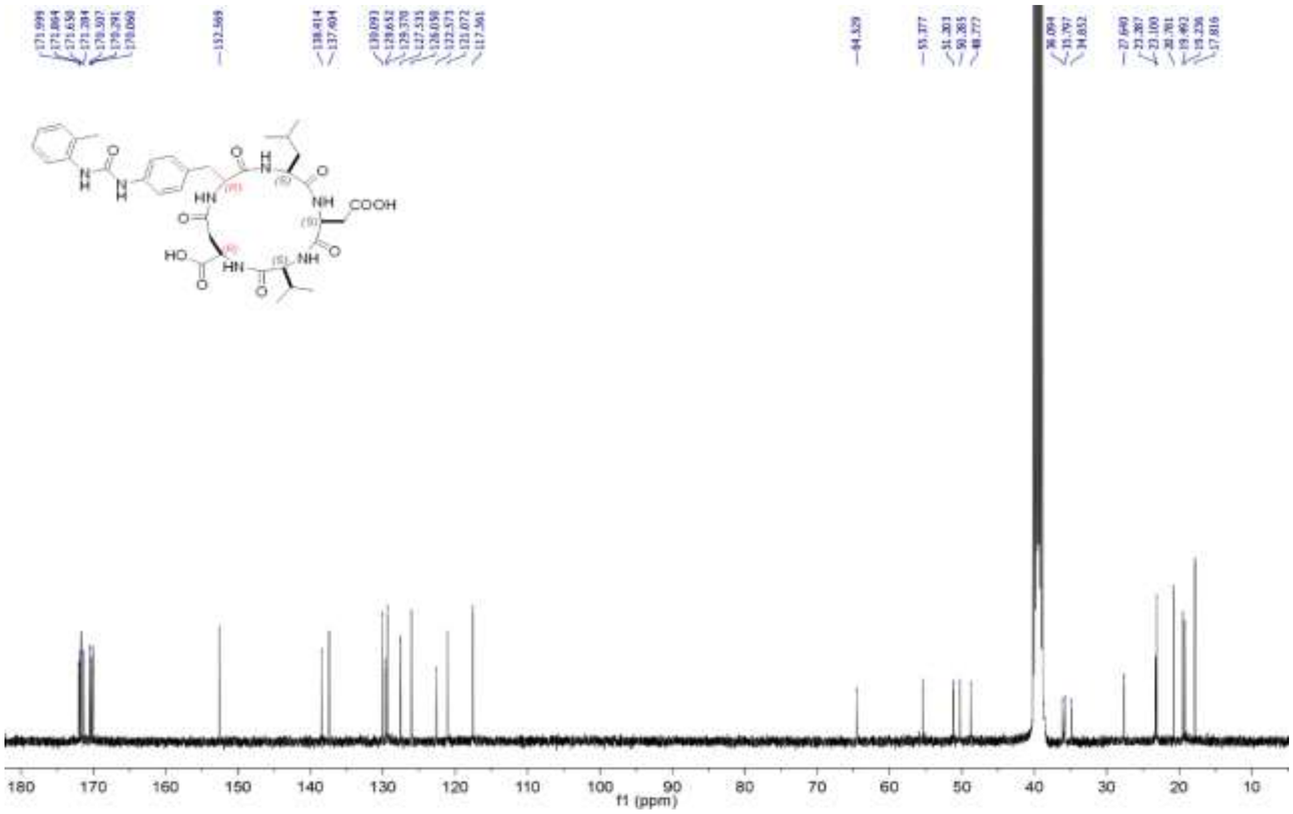

Figure S25. ^{1}H-NMR (8:2 DMSOd$_6$/H_2O at 400 MHz) and ^{13}C-NMR (DMSOd$_6$, 100 MHz) of **3d**.

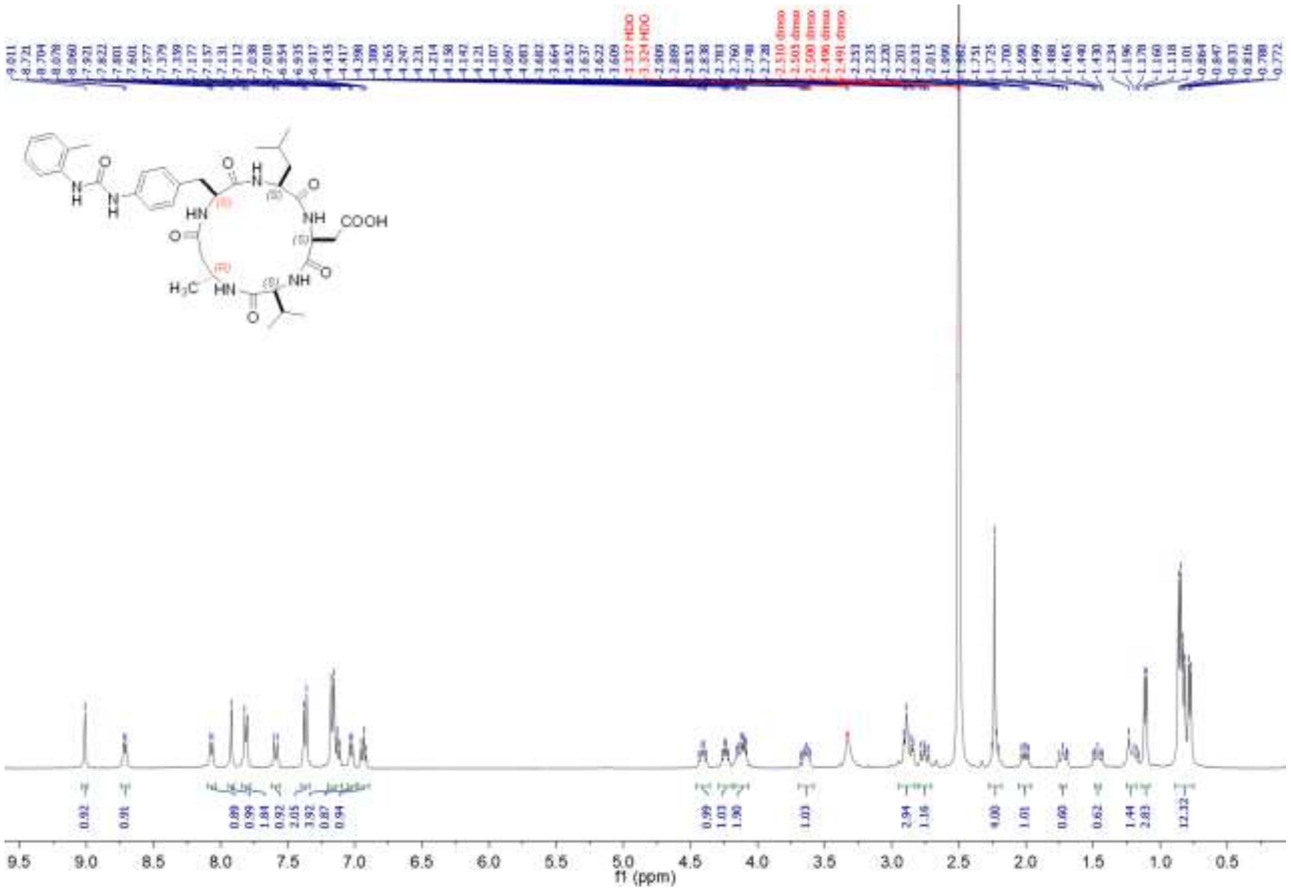

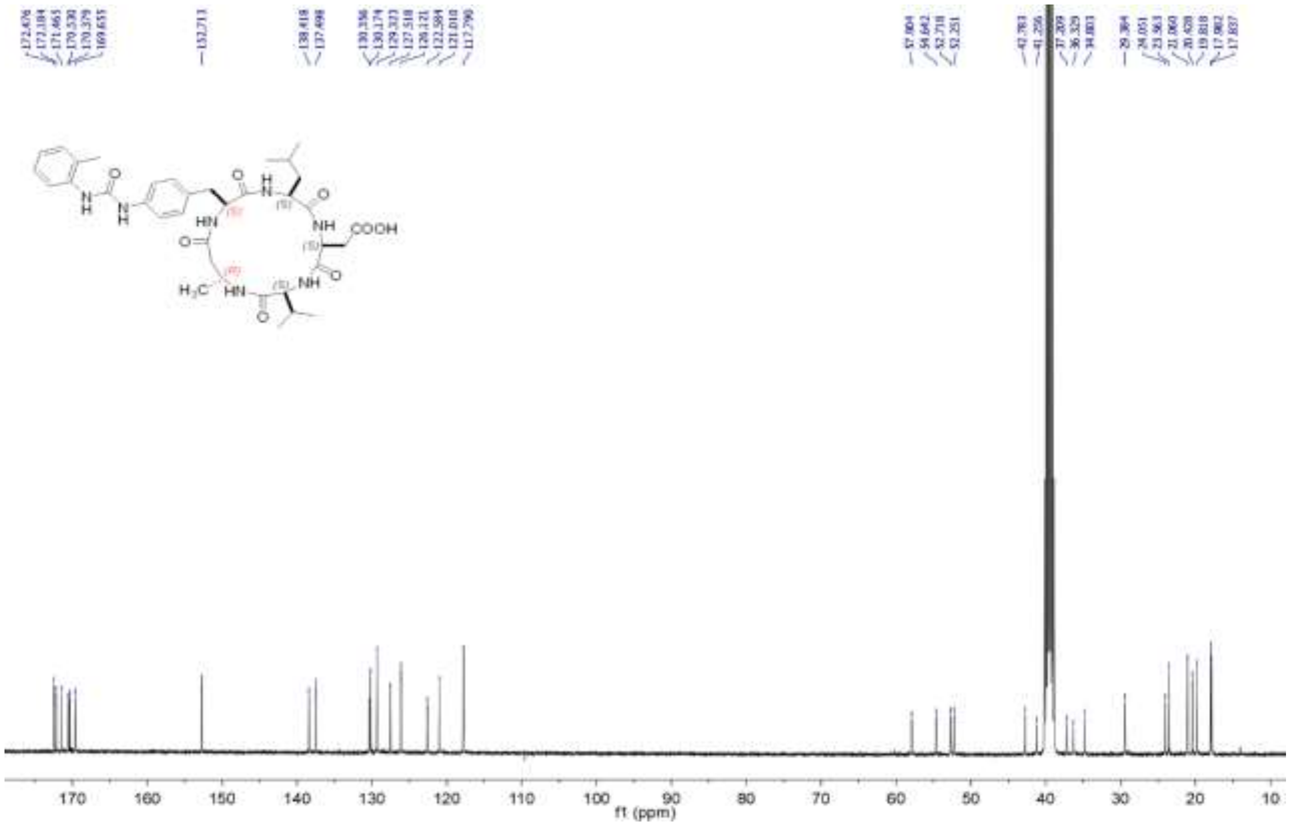

Figure S26. ^{1}H-NMR (8:2 DMSOd$_6$/H_2O at 400 MHz) and ^{13}C-NMR (DMSOd$_6$, 100 MHz) of **11a**.

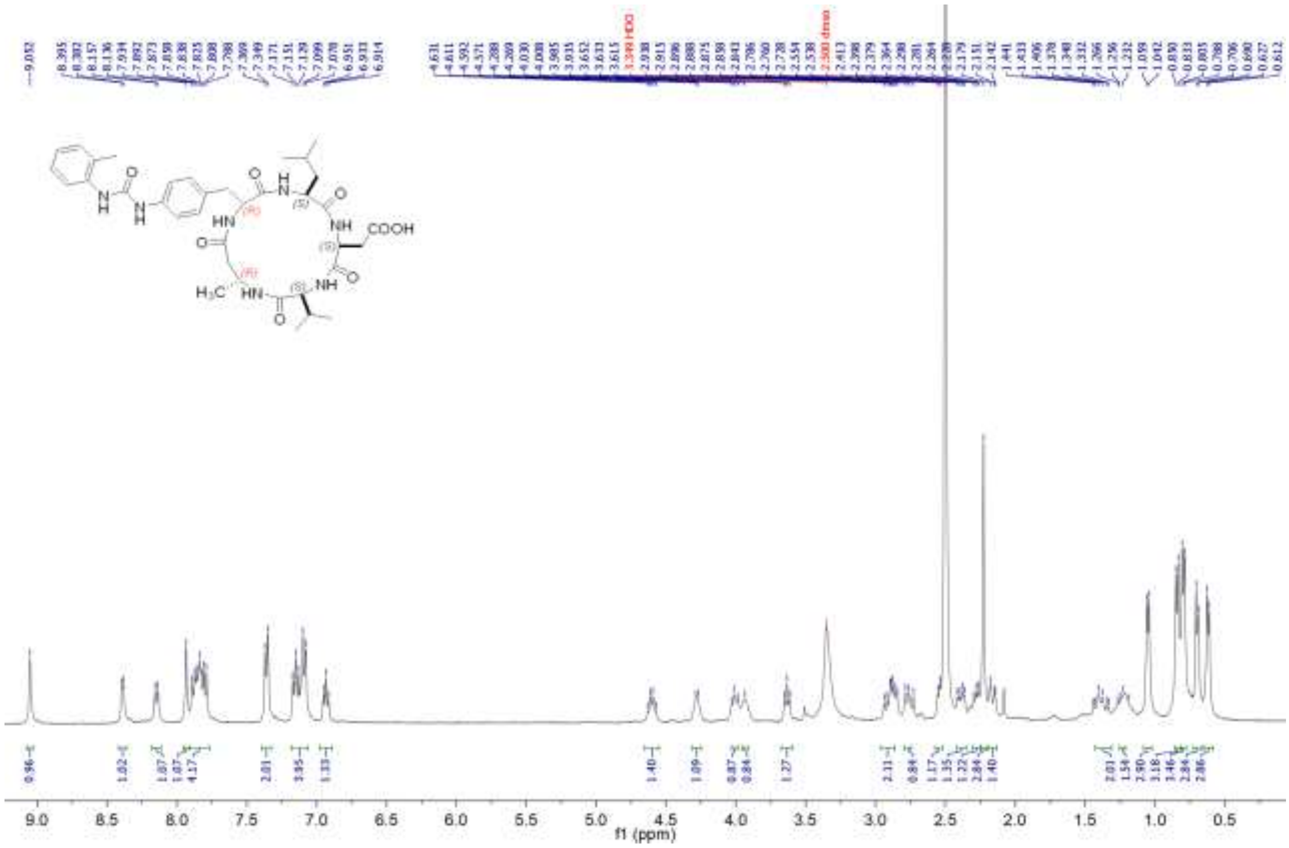

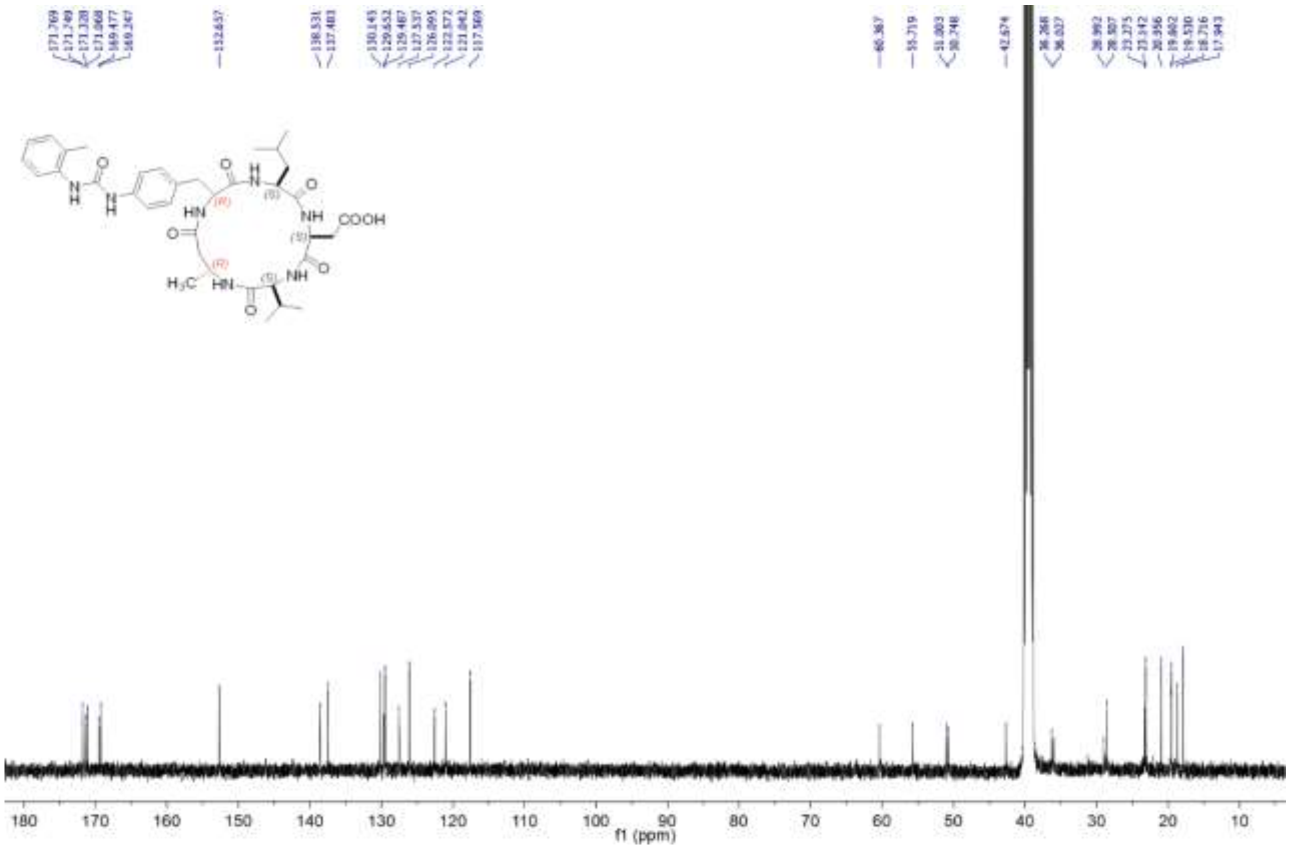

Figure S27. ^{1}H-NMR (8:2 $DMSOd_6/H_2O$ at 400 MHz) and ^{13}C-NMR ($DMSOd_6$, 100 MHz) of **11c**.

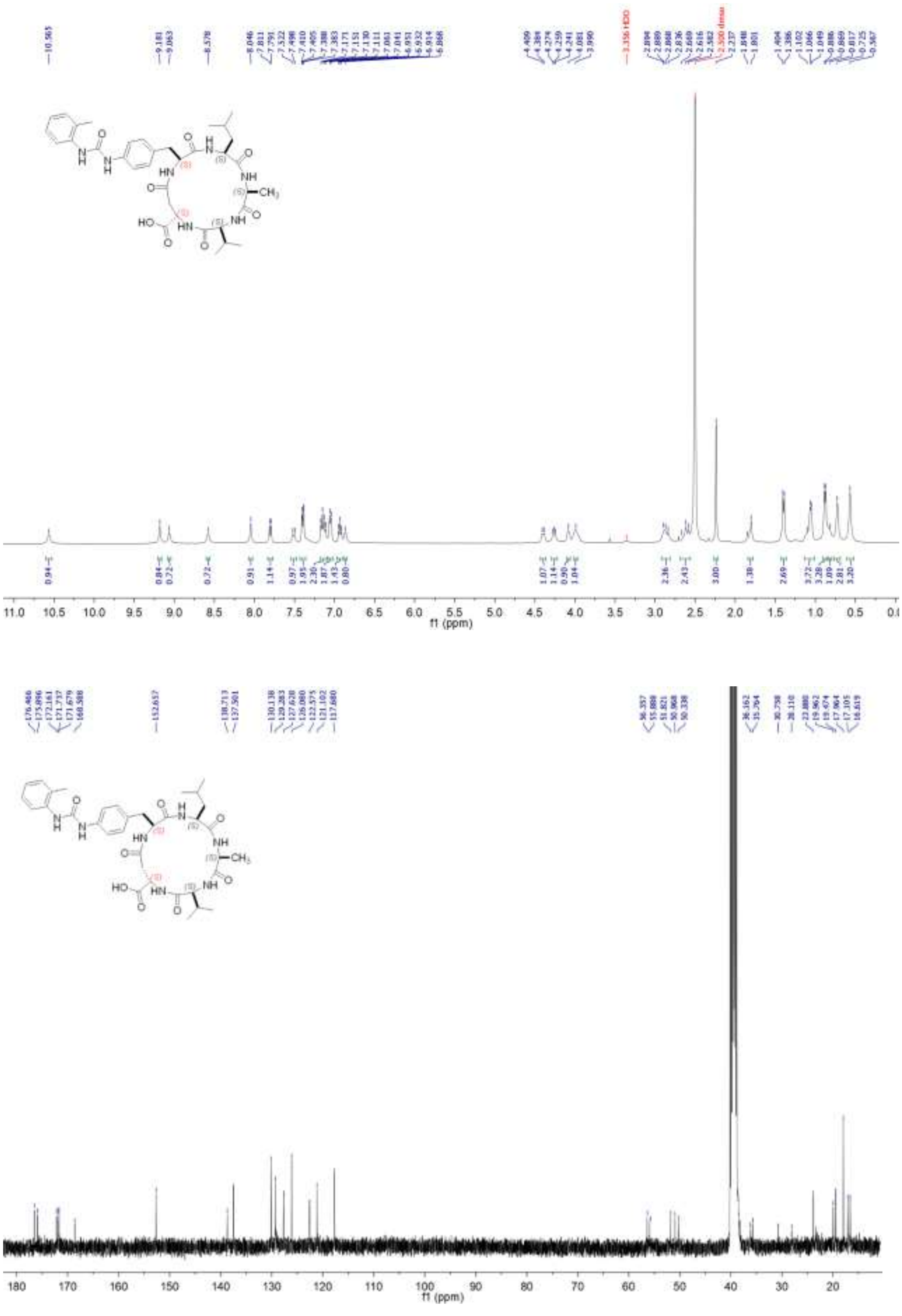

Figure S28. ^{1}H-NMR (8:2 DMSOd$_6$/H_2O at 400 MHz) and ^{13}C-NMR (DMSOd$_6$, 100 MHz) of **12a**.

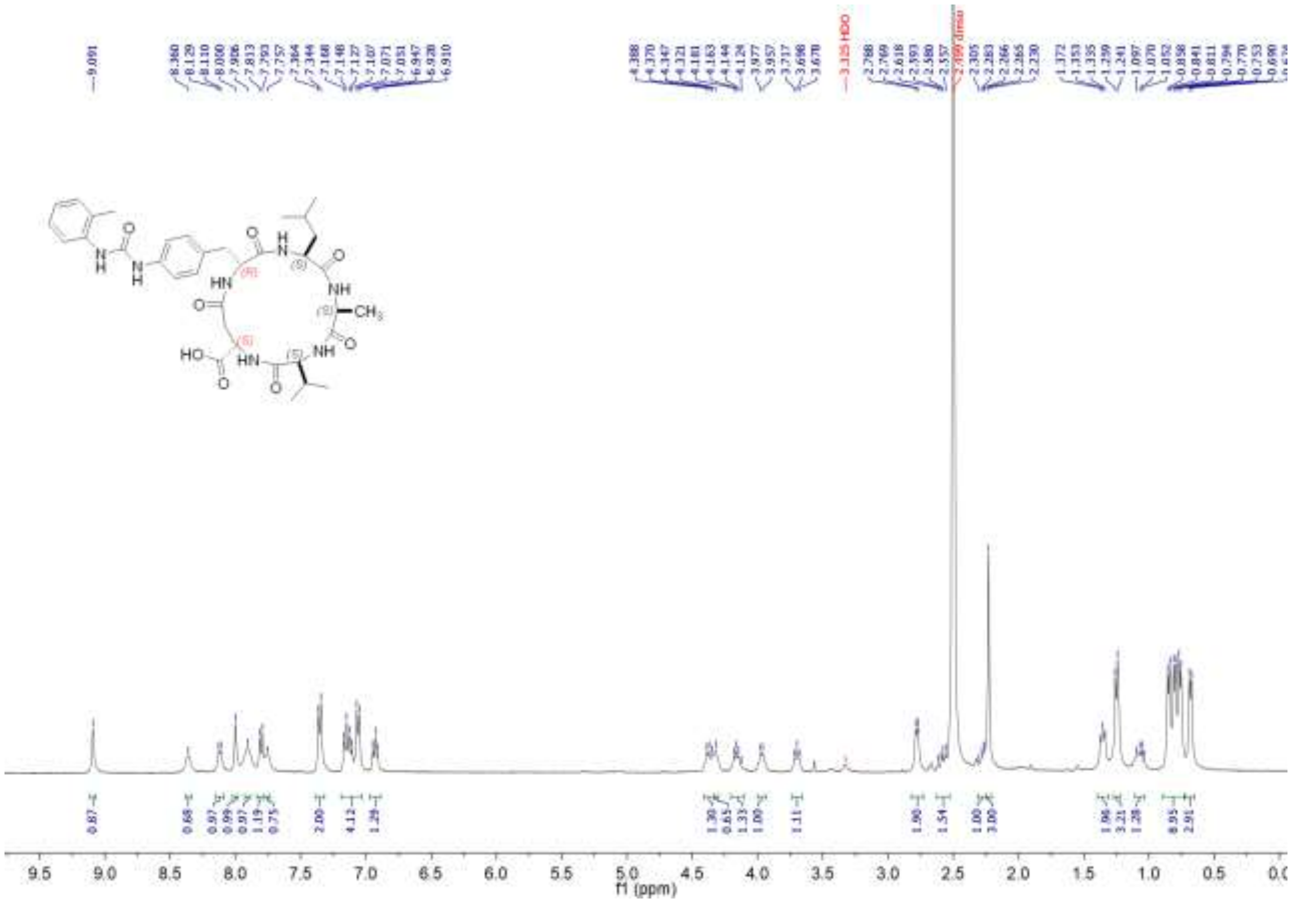

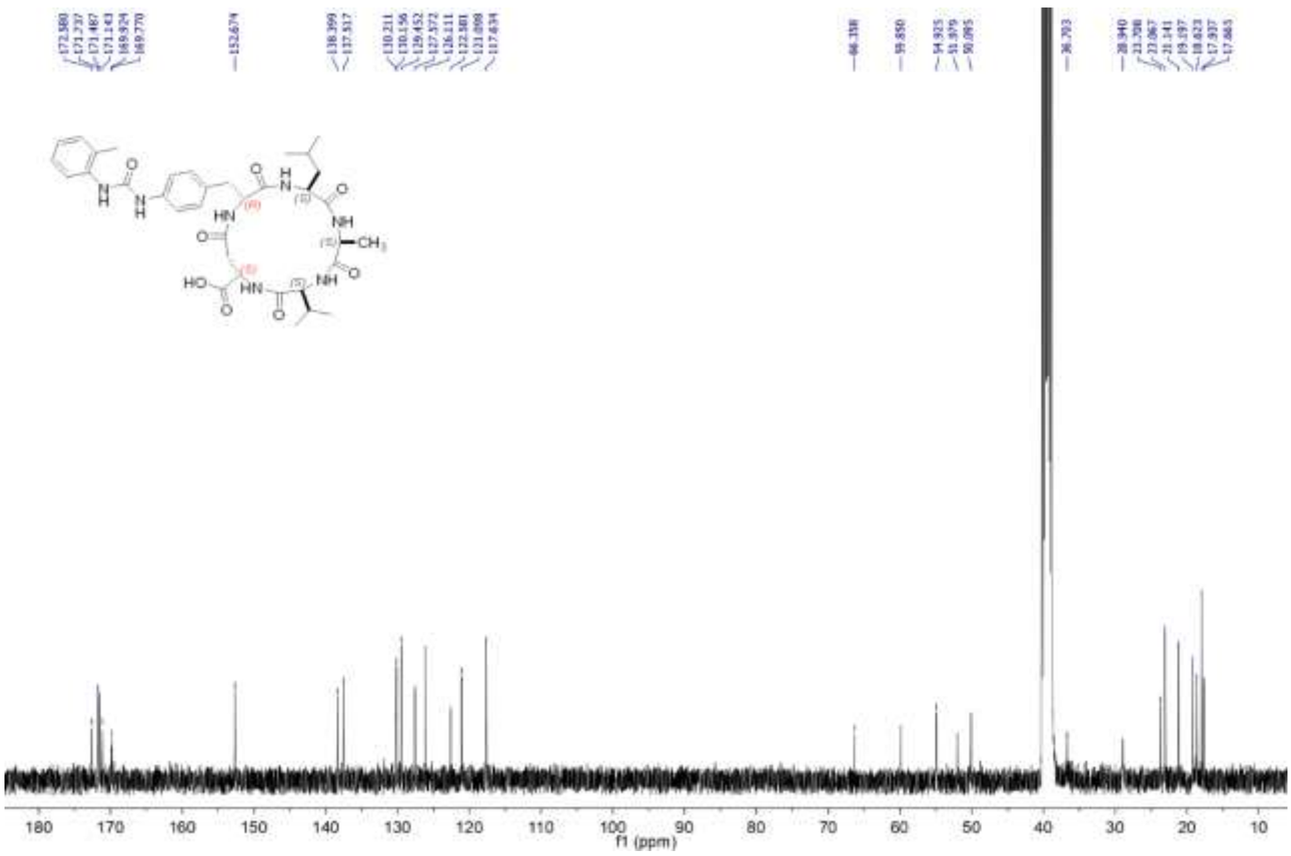

Figure S29. ^{1}H-NMR (8:2 DMSOd$_6$/H_2O at 400 MHz) and ^{13}C-NMR (DMSOd$_6$, 100 MHz) of **12c**.

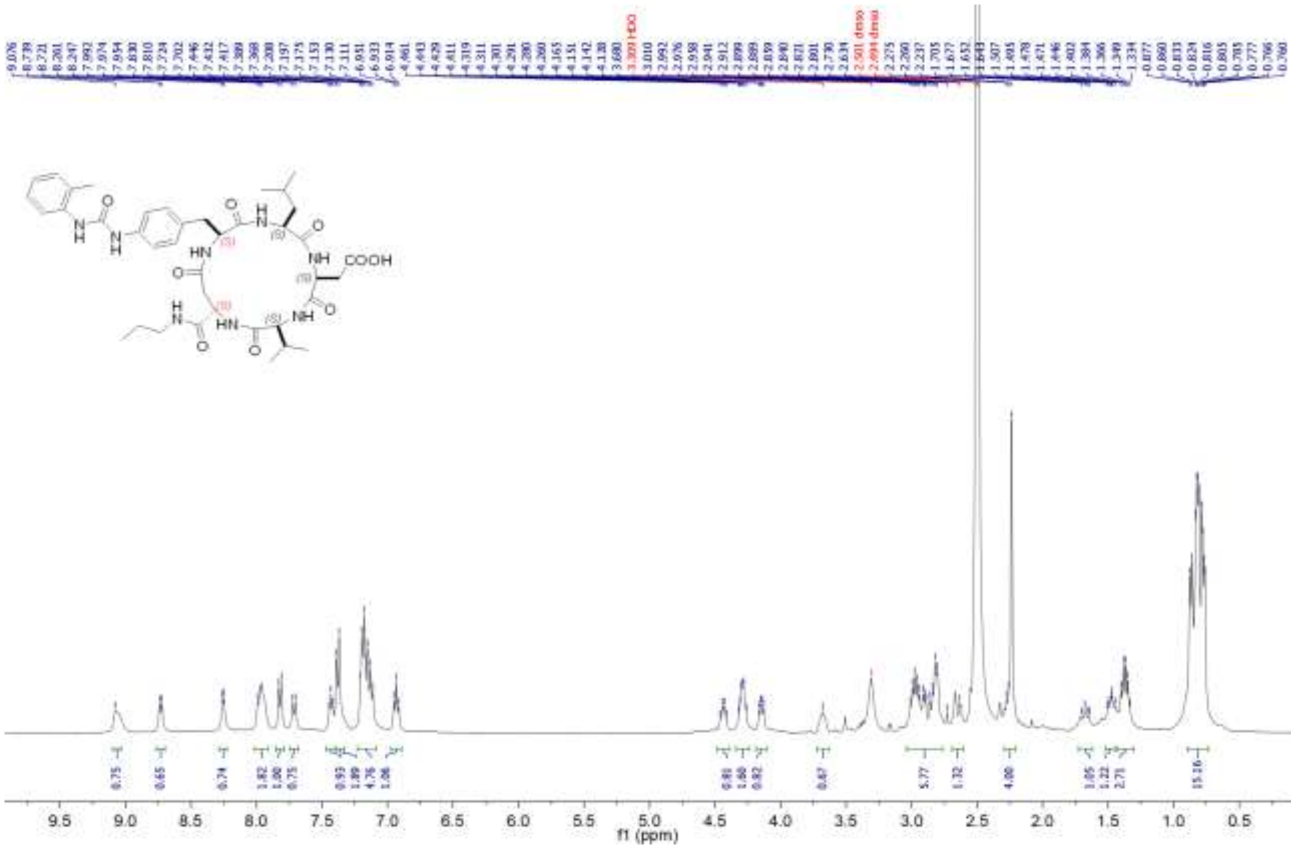

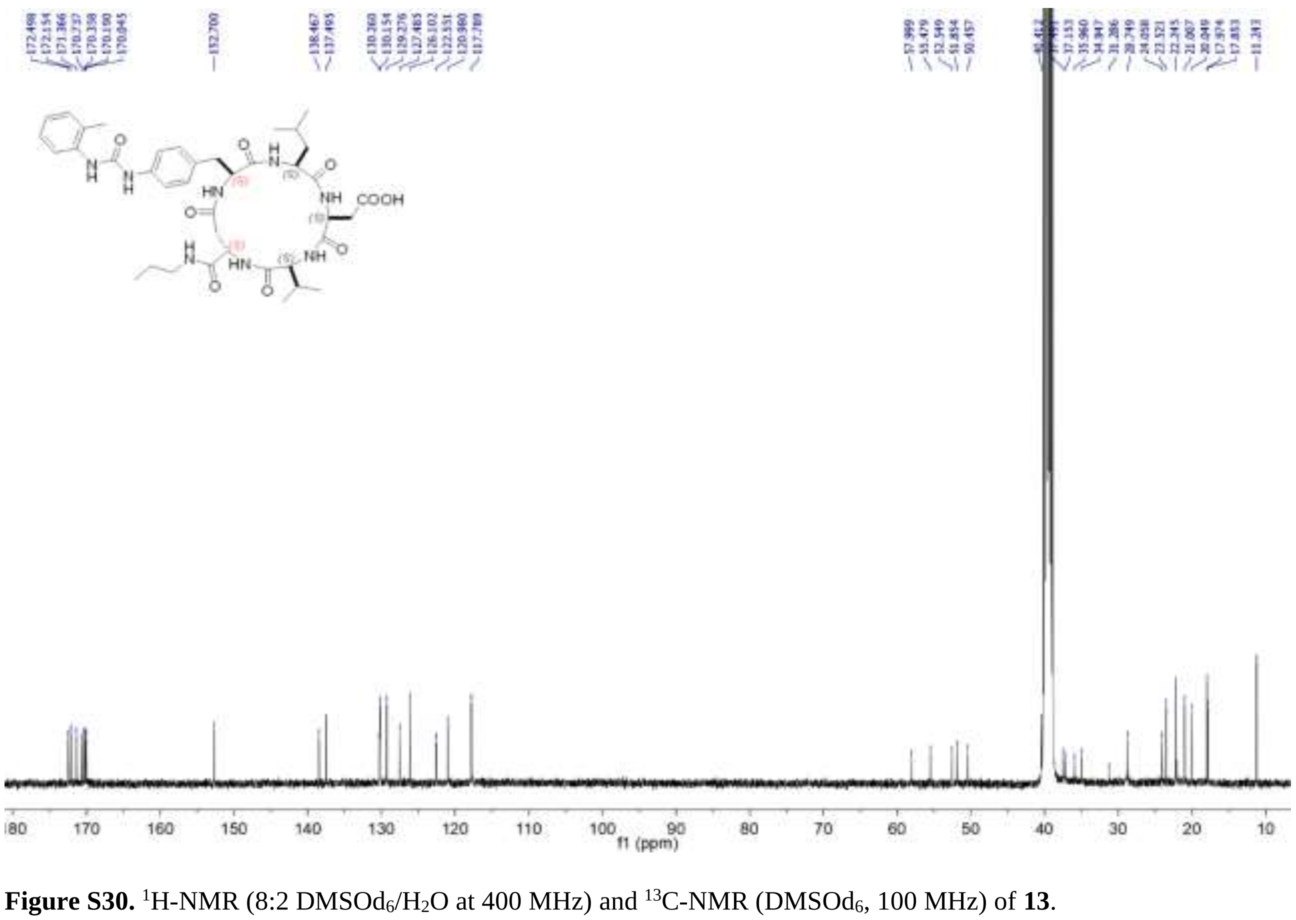

Figure S30. ^{1}H-NMR (8:2 $DMSOd_6/H_2O$ at 400 MHz) and ^{13}C-NMR ($DMSOd_6$, 100 MHz) of **13**.

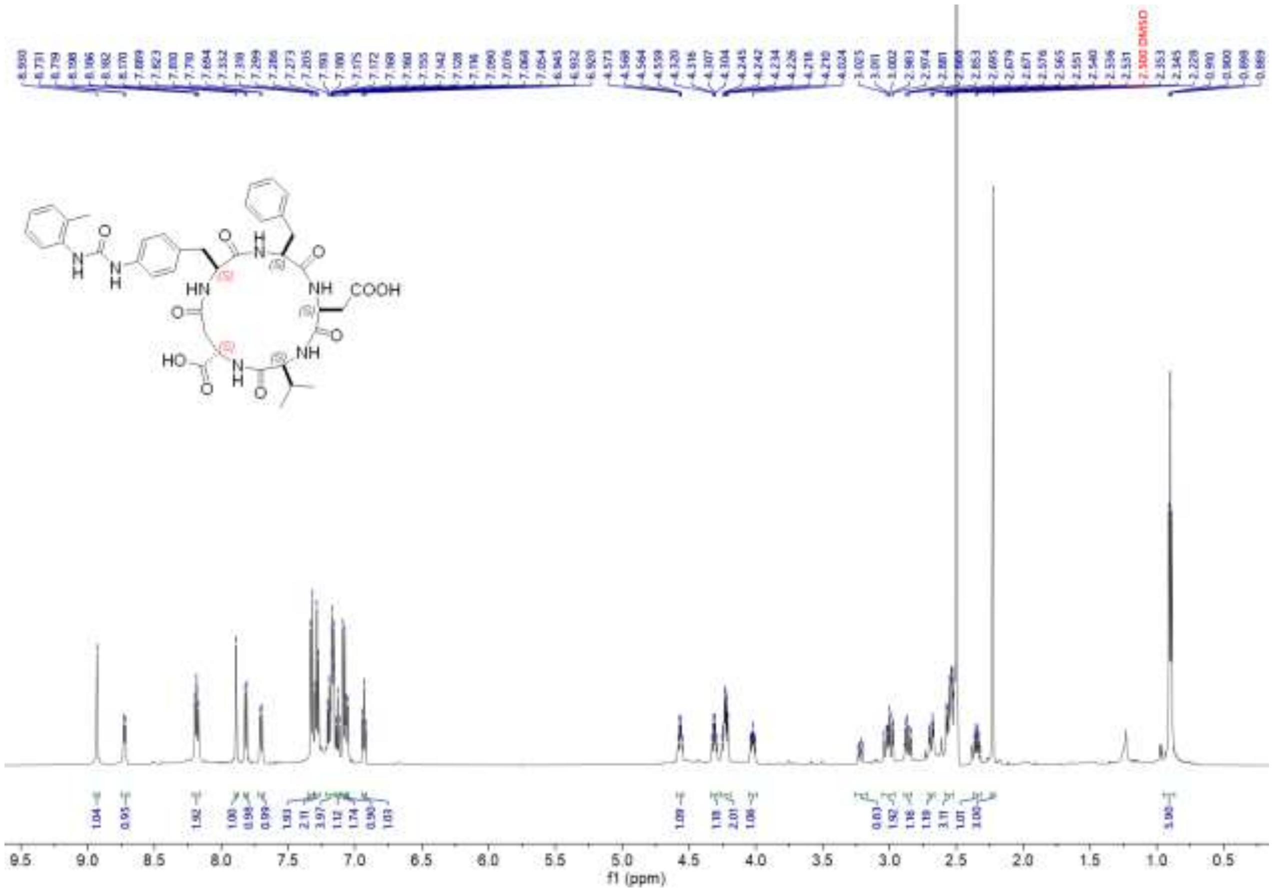

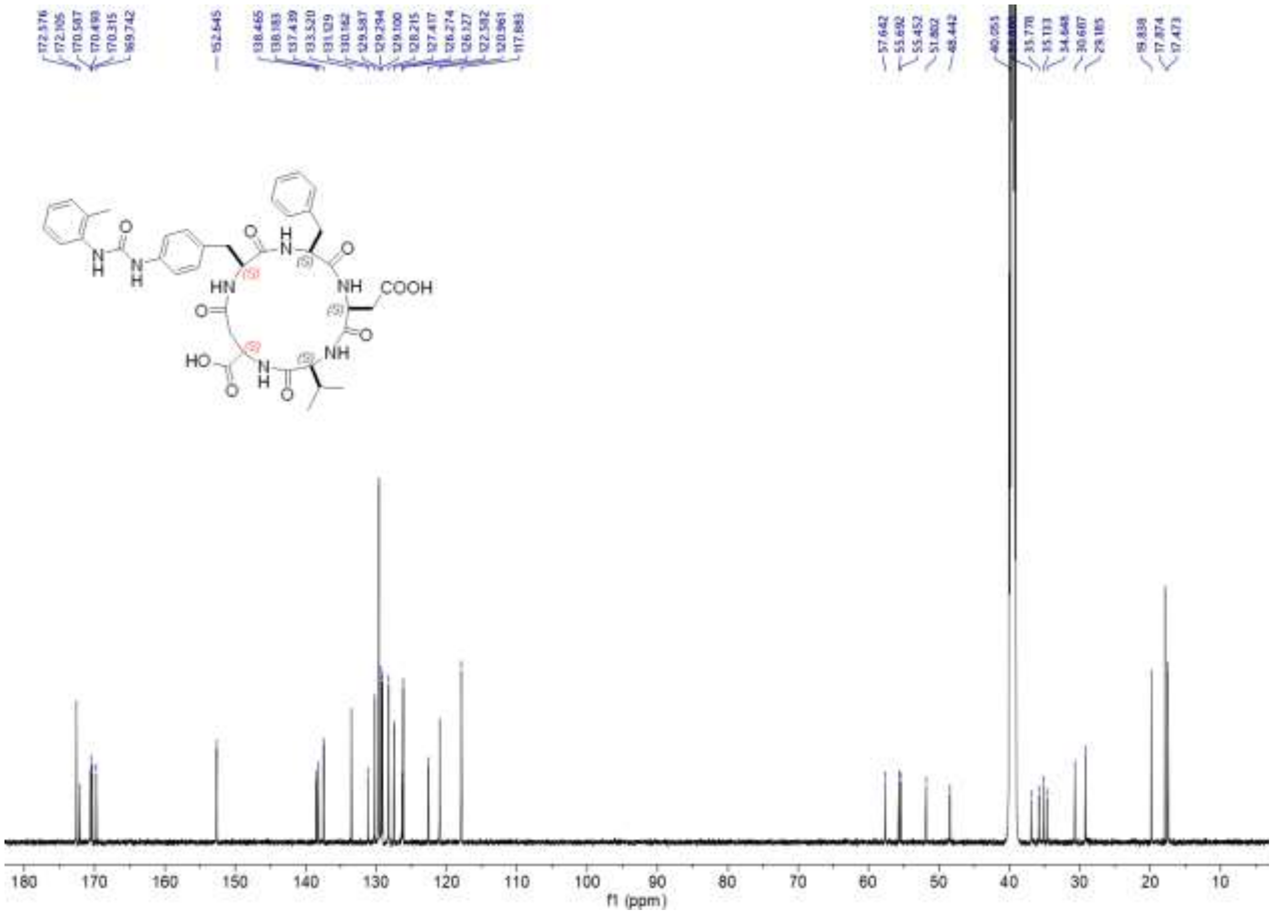

Figure S31. ^{1}H-NMR (8:2 DMSOd$_6$/H_2O at 600 MHz) and ^{13}C-NMR (DMSOd$_6$, 150 MHz) of **14**.

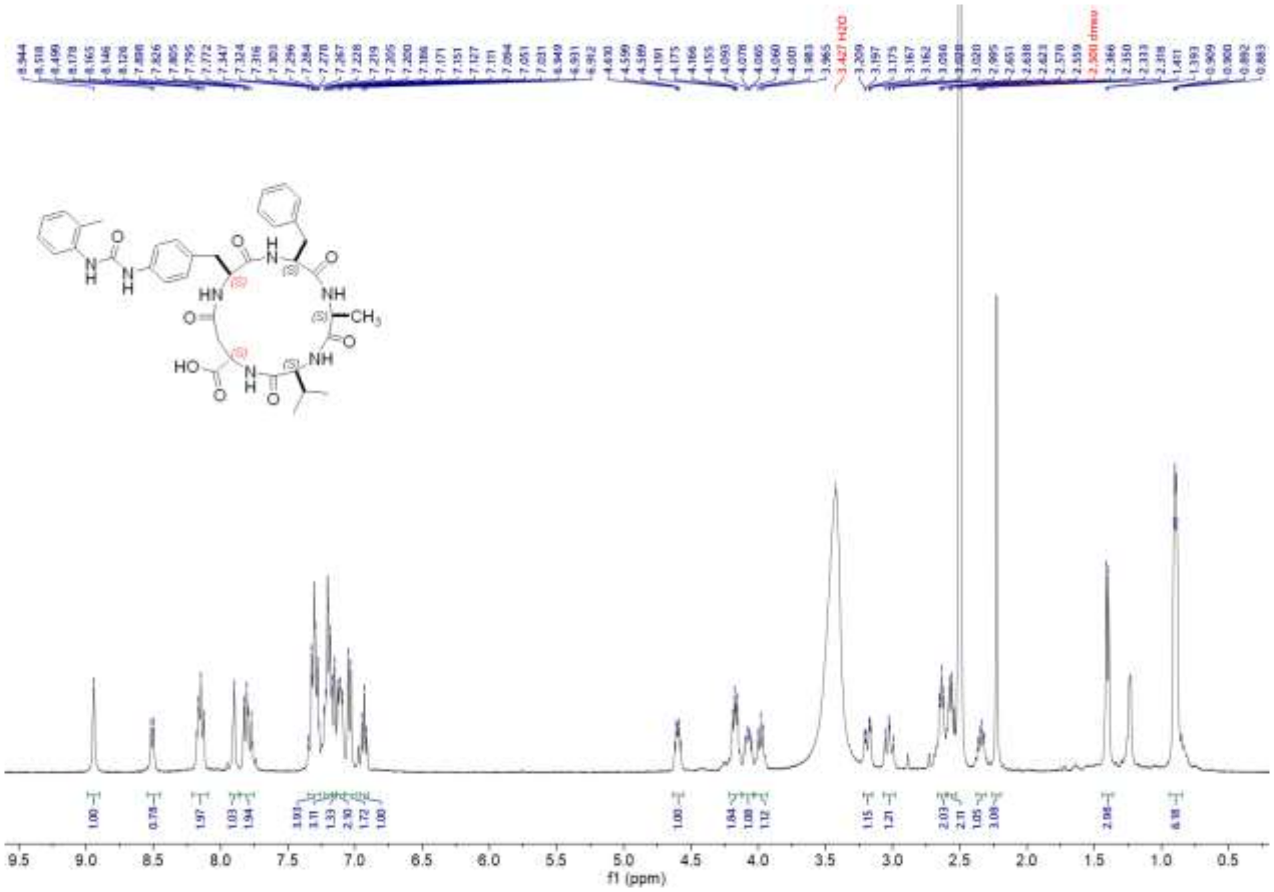

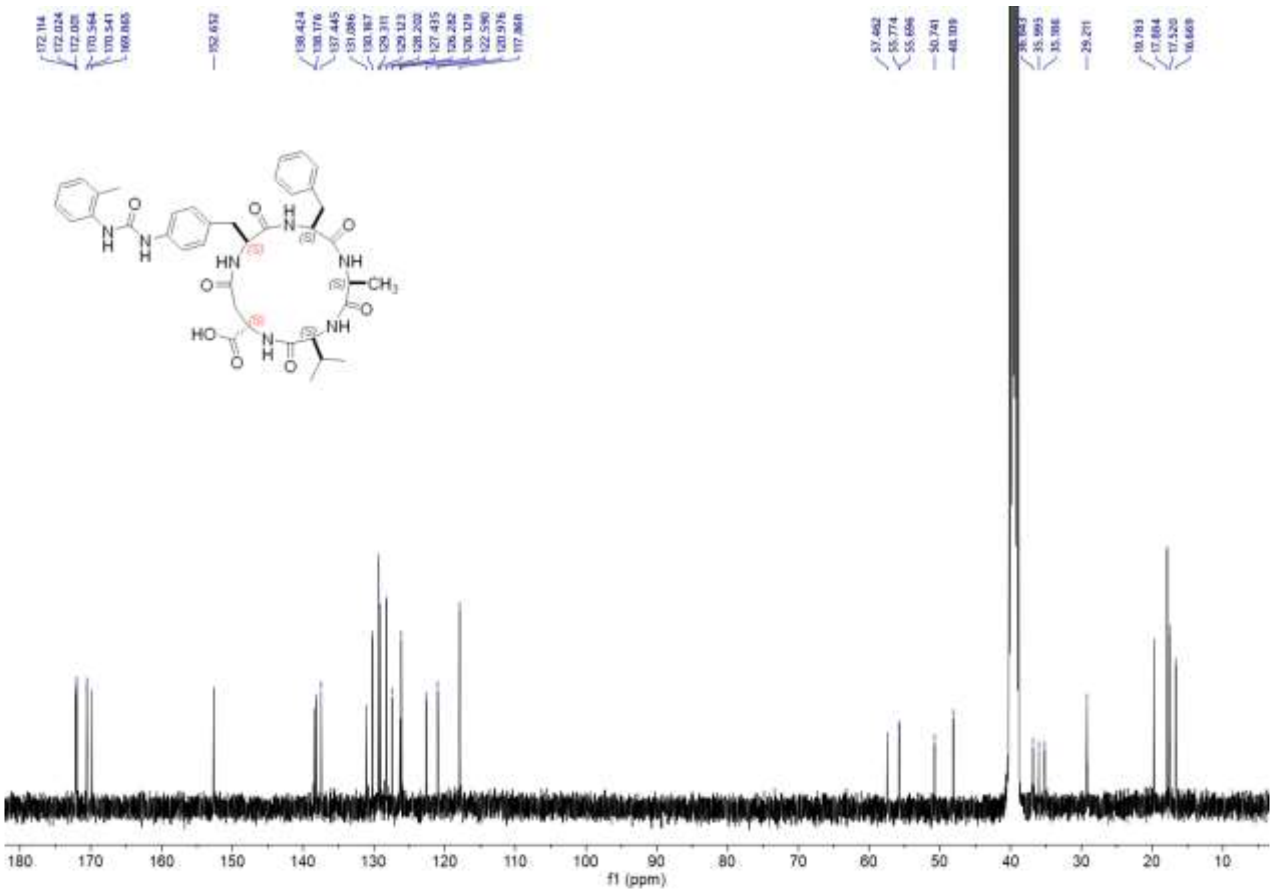

Figure S32. ^{1}H-NMR (8:2 DMSOd$_6$/H_2O at 400 MHz) and ^{13}C-NMR (DMSOd$_6$, 100 MHz) of **15**.

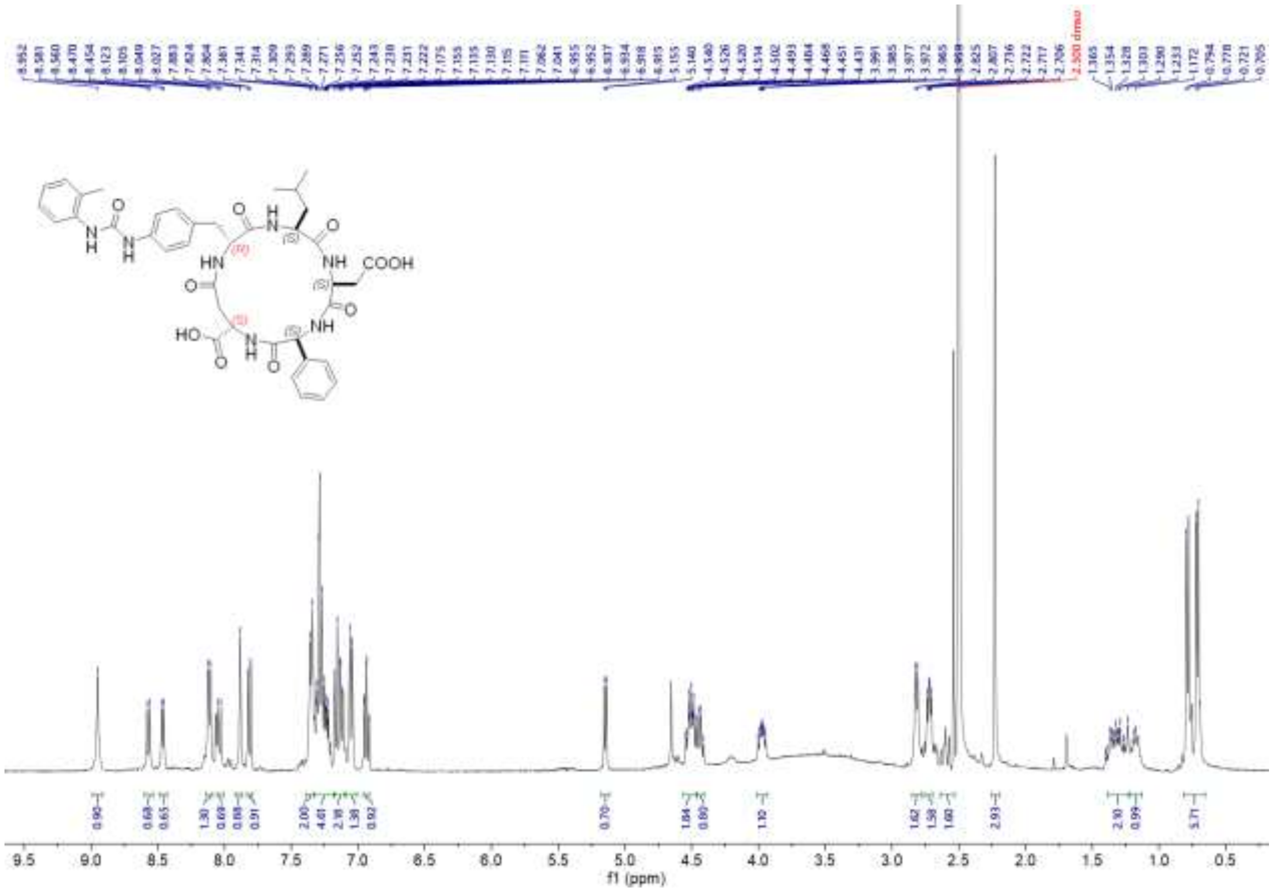

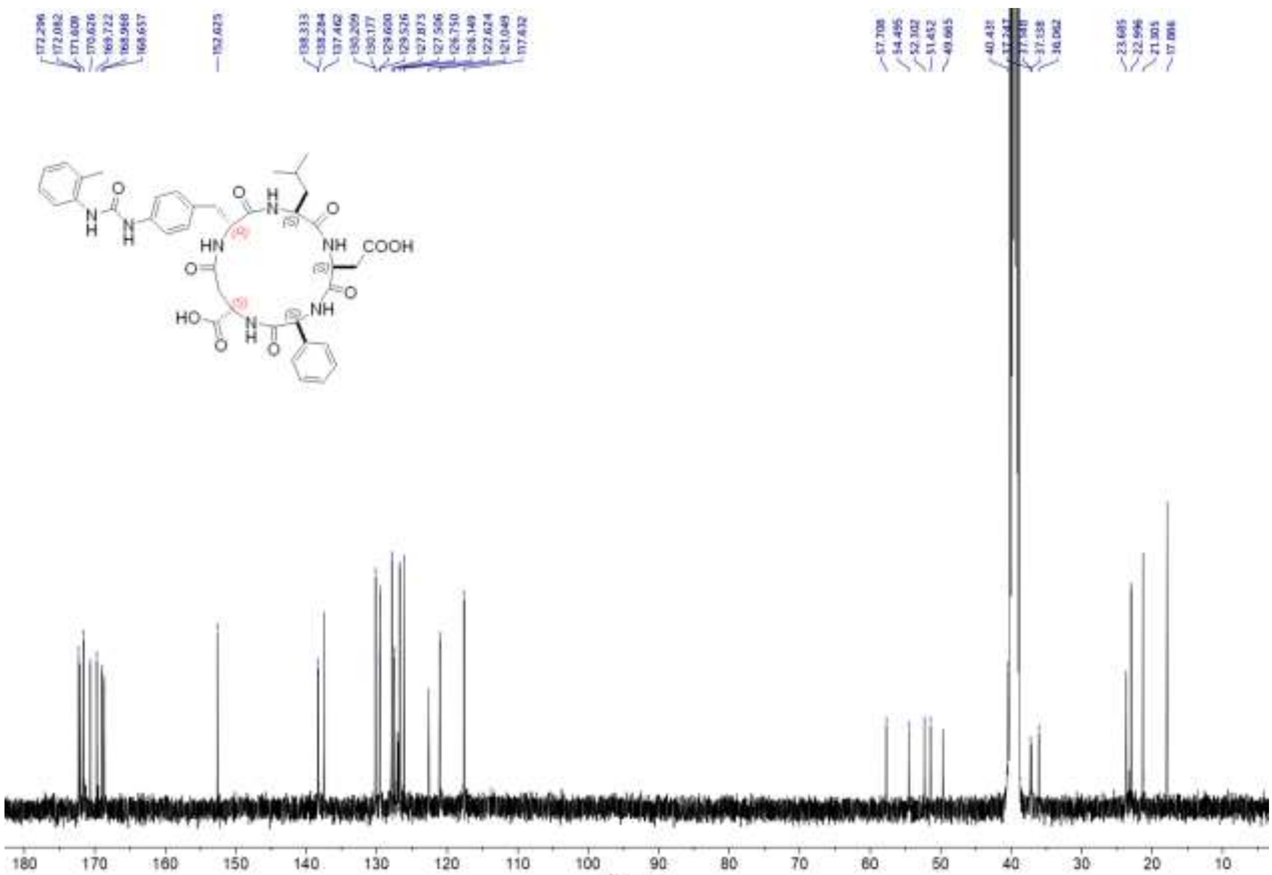

Figure S33. ^{1}H-NMR (8:2 DMSOd$_6$/H$_2$O at 400 MHz) and ^{13}C-NMR (DMSOd$_6$, 100 MHz) of **16**.

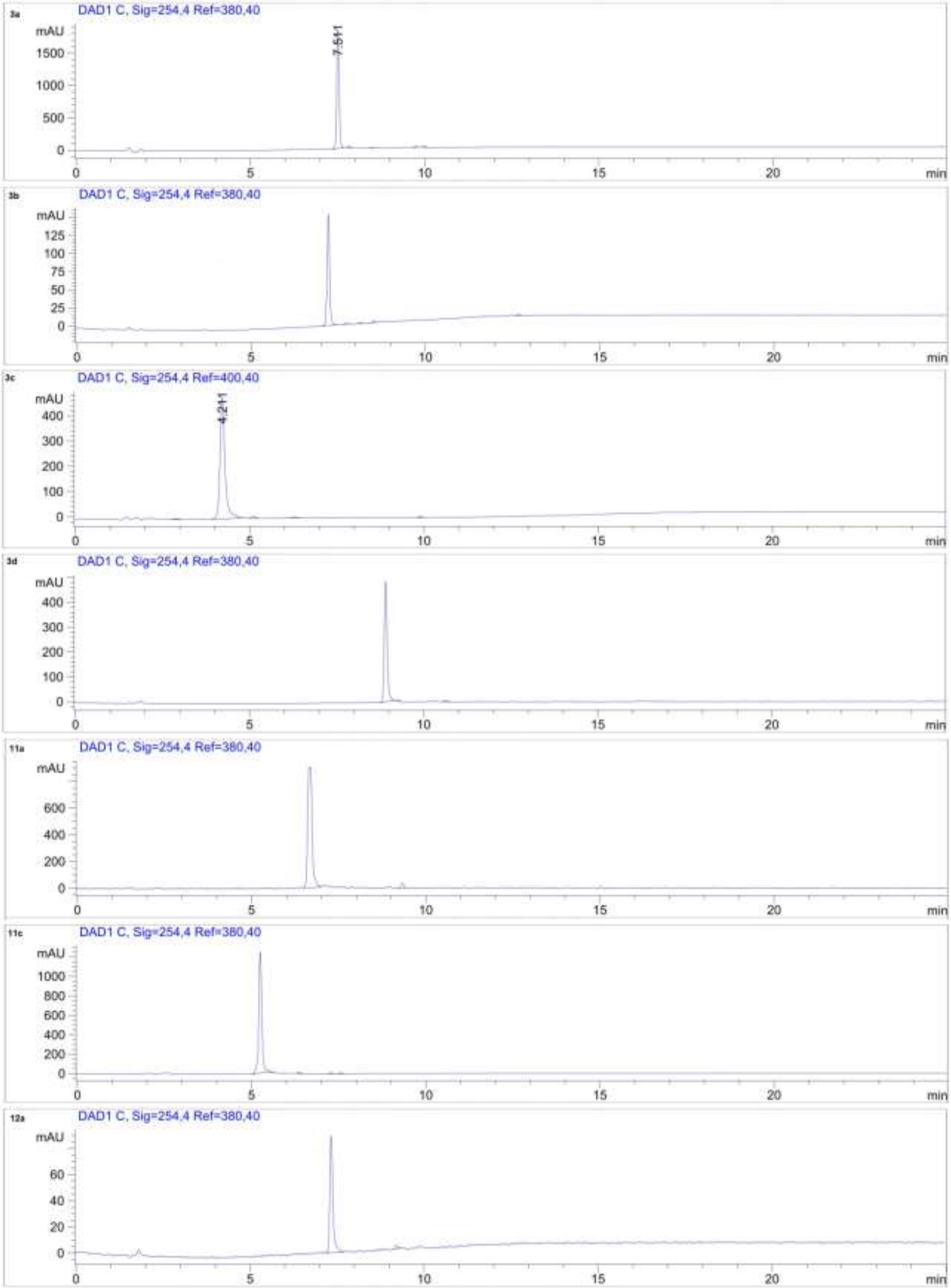

Figure S34. RP HPLC analyses performed on a column Phenomenex mod. Gemini 3 µm C_{18} 110 Å 100 × 3.0 mm; mobile phase from 9:1 H_2O/CH_3CN/0.1% HCOOH to 2:8 H_2O/CH_3CN/0.1% HCOOH in 20 min, flow rate of 1.0 mL min^{-1}. DAD 254 nm, unless otherwise specified. For peptide **14**, the analytical flow rate is 0.5 mL min^{-1}.

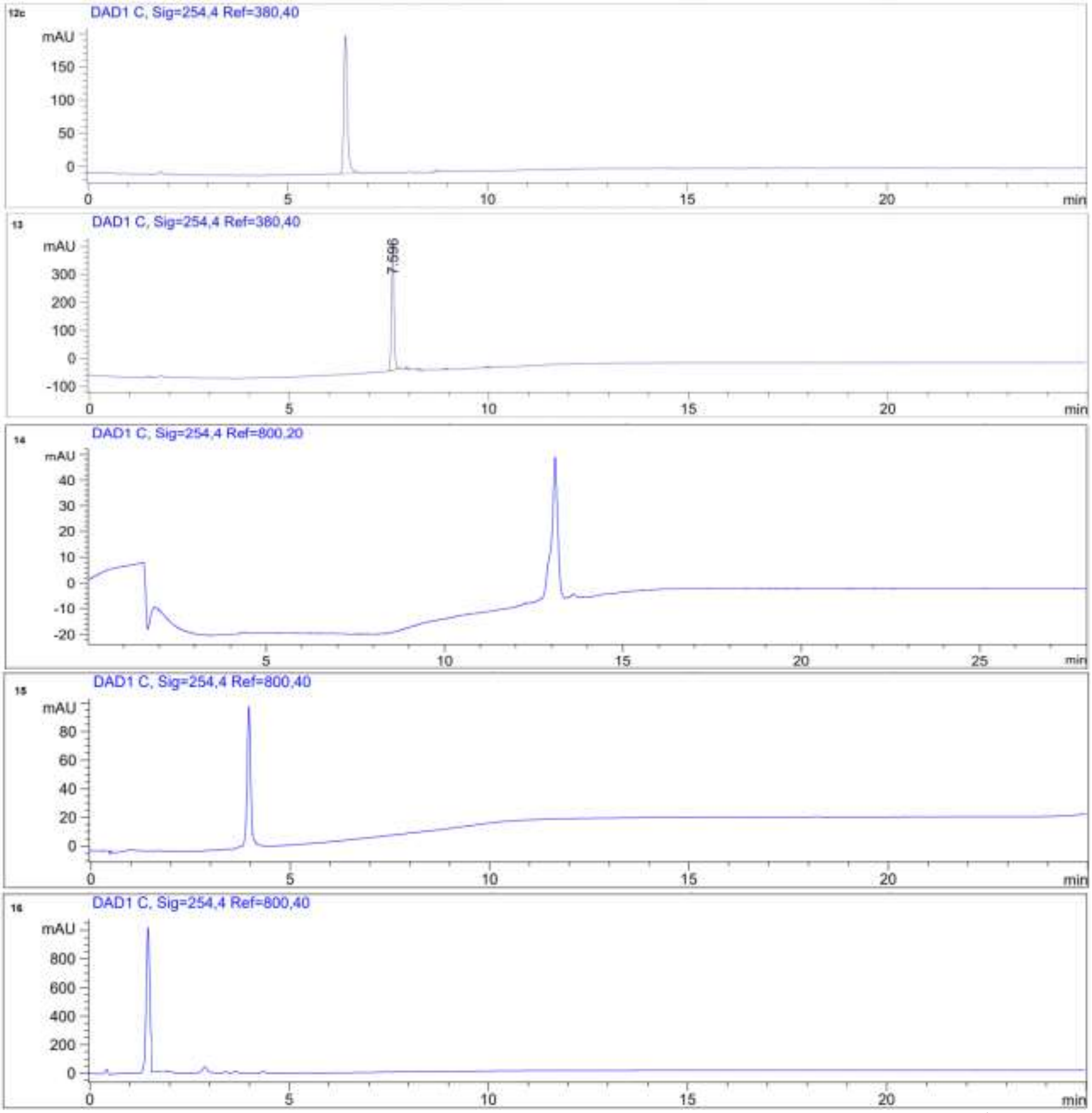

Figure S34-follows**.** RP HPLC analyses performed on a column Phenomenex mod. Gemini 3 µm C_{18} 110 Å 100 × 3.0 mm; mobile phase from 9:1 H_2O/CH_3CN/0.1% HCOOH to 2:8 H_2O/CH_3CN/0.1% HCOOH in 20 min, flow rate of 1.0 mL min^{-1}. DAD 254 nm, unless otherwise specified. For peptide **14**, the analytical flow rate is 0.5 mL min^{-1}.
